# Supplementary material for: Structure-guided design of a selective inhibitor of the methyltransferase KMT9 with cellular activity
Source: Nat Commun. 2024 Jan 2;15:43. doi: 10.1038/s41467-023-44243-6 (PMC10762027; doi:10.1038/s41467-023-44243-6)
Supplement: Supplementary file 2 — Description of Additional Supplementary Files [file 41467_2023_44243_MOESM2_ESM.pdf]

Description of Additional Supplementary Files:

Supplementary Data 1: Mass spectrometry data of the final compounds.
