# Supplementary material for: Structure-guided design of a selective inhibitor of the methyltransferase KMT9 with cellular activity
Source: Nat Commun. 2024 Jan 2;15:43. doi: 10.1038/s41467-023-44243-6 (PMC10762027; doi:10.1038/s41467-023-44243-6)

Supplemental data: Mass spectrometry

Compound 1a

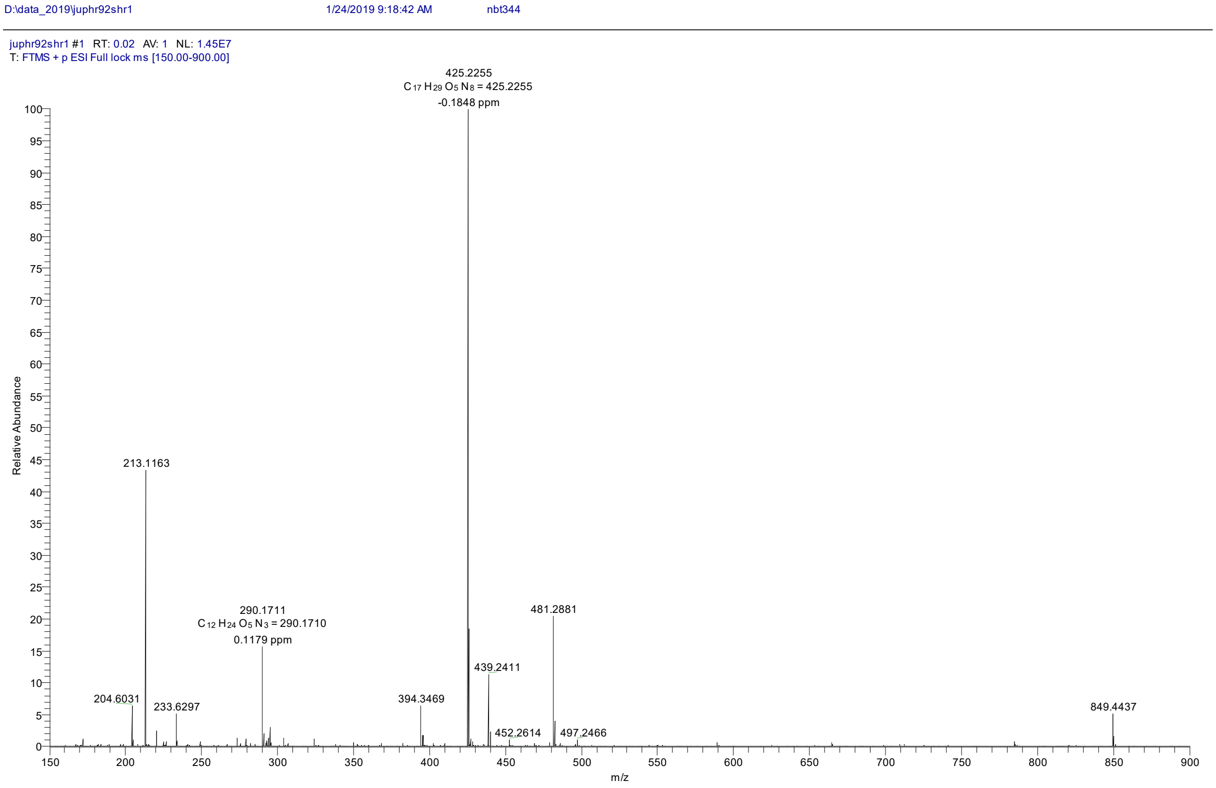

Compound 2b

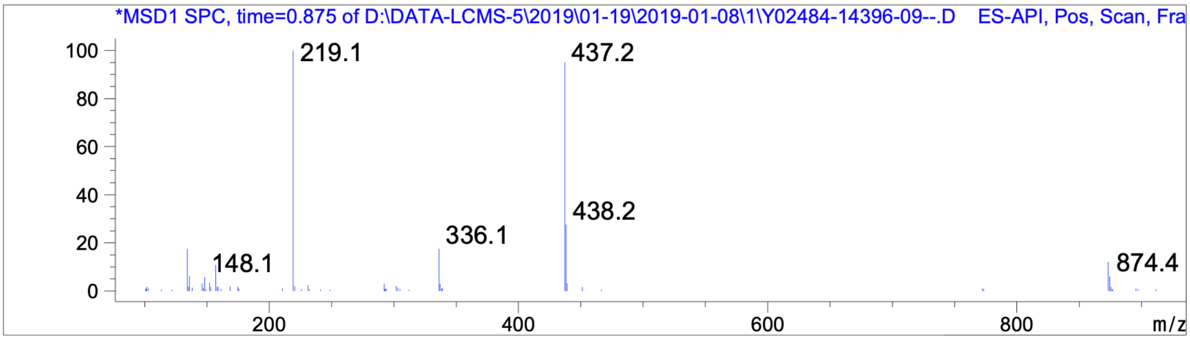

Compound 1c

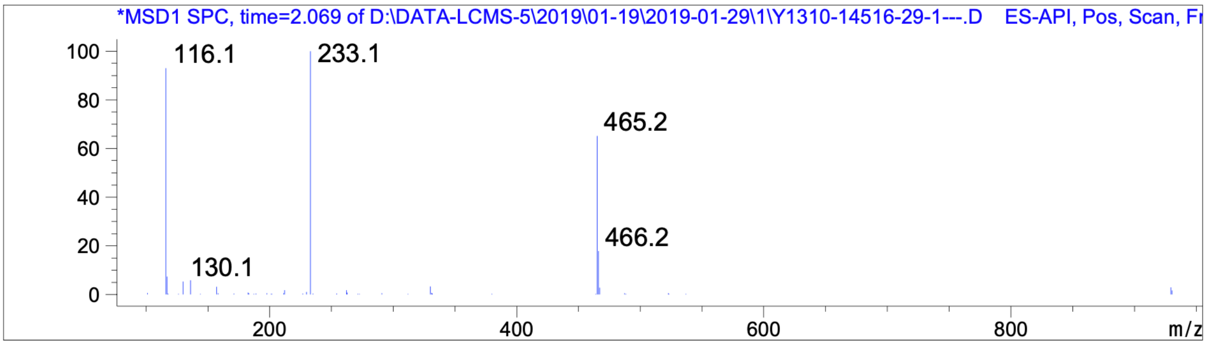

Compound 2a

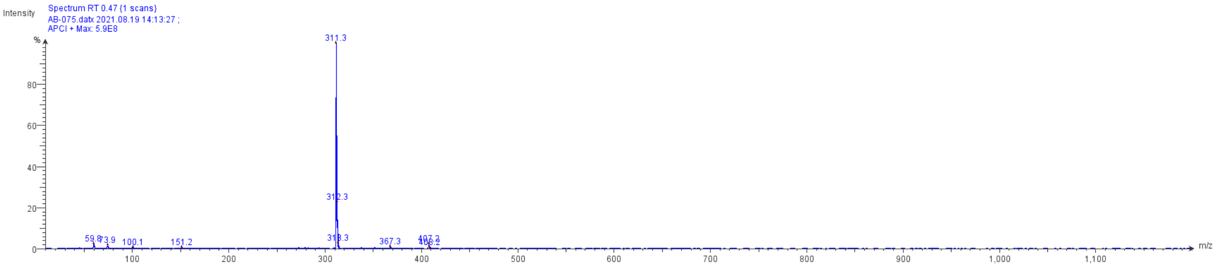

Compound 2b

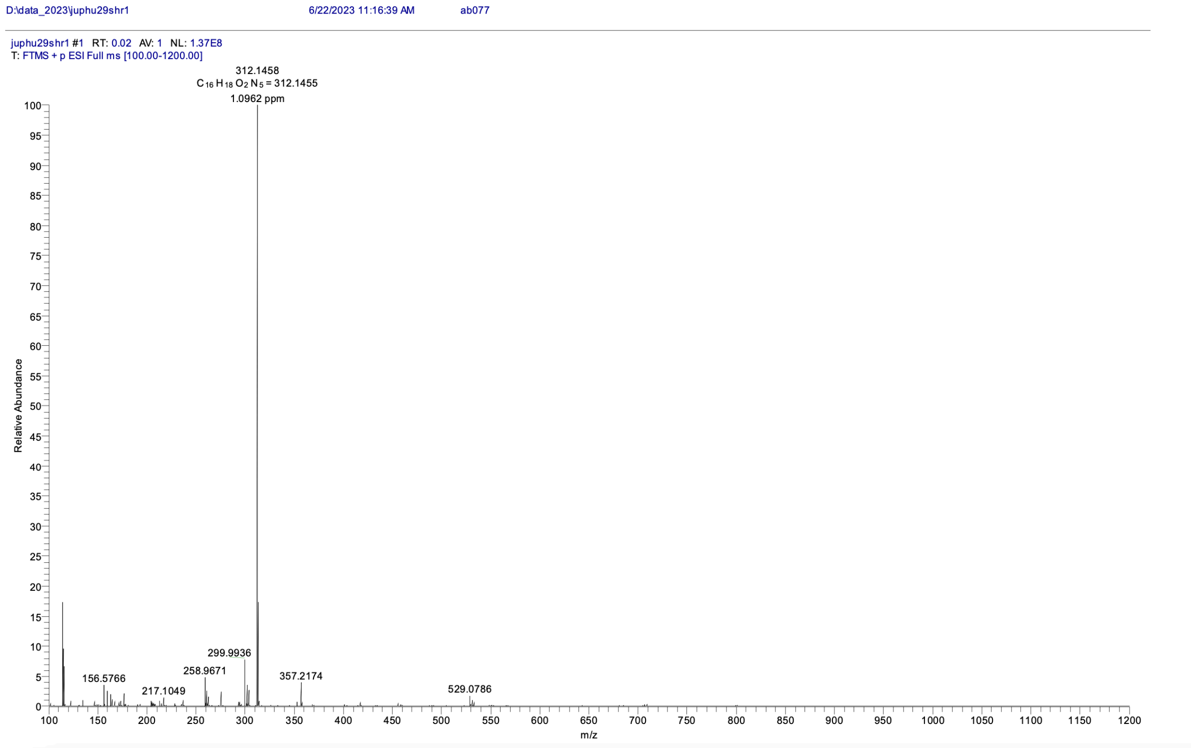

Compound 2c

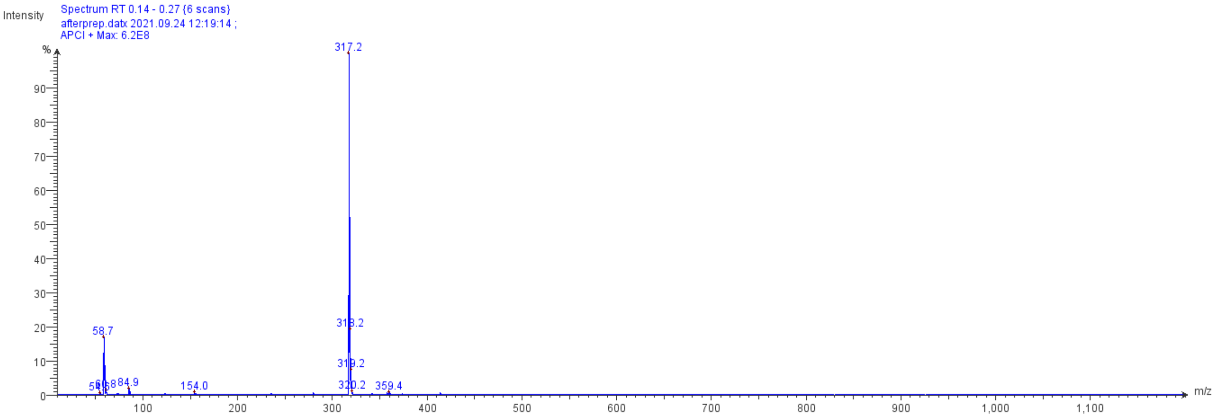

Compound 2d

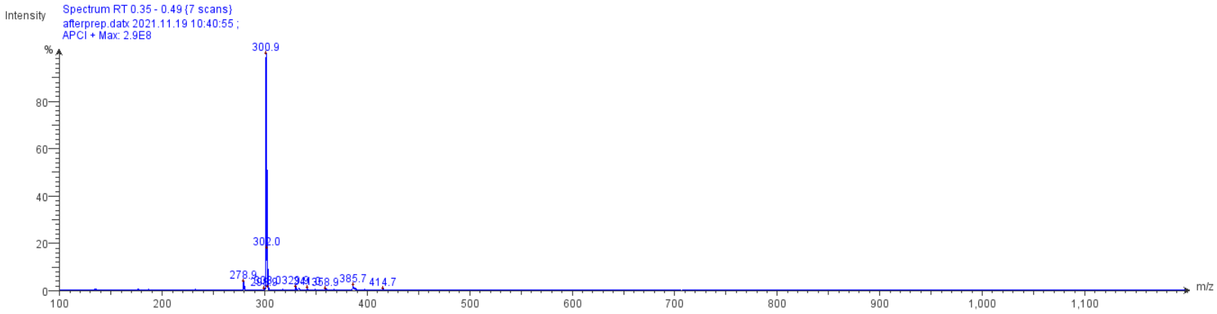

Compound 2e

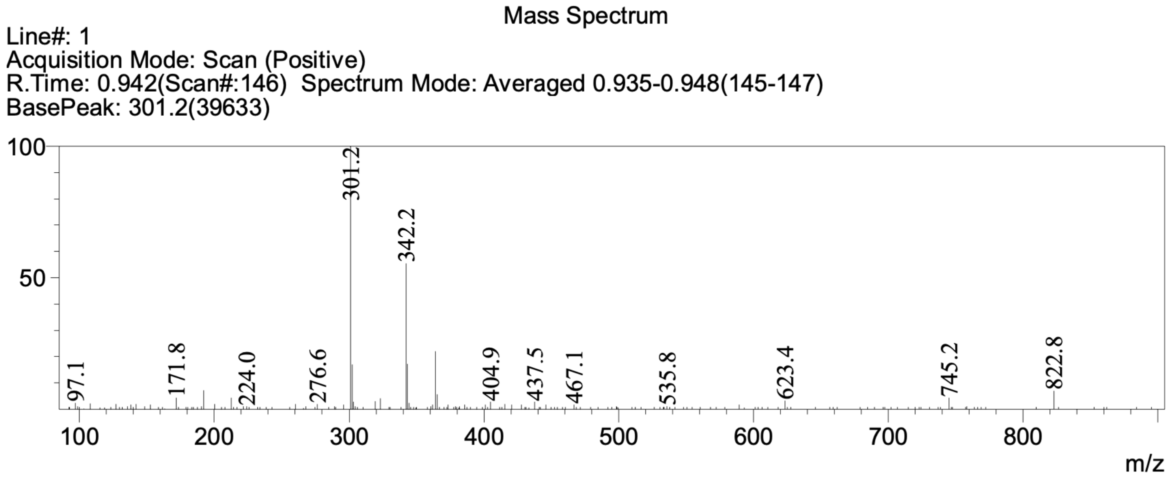

Compound 2f

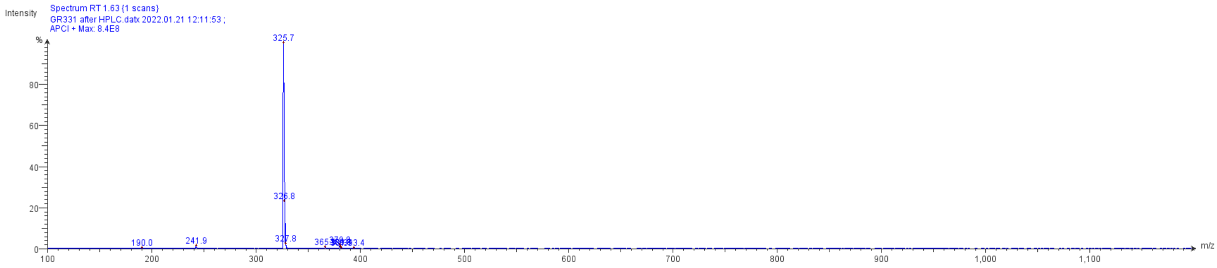

Compound 2g

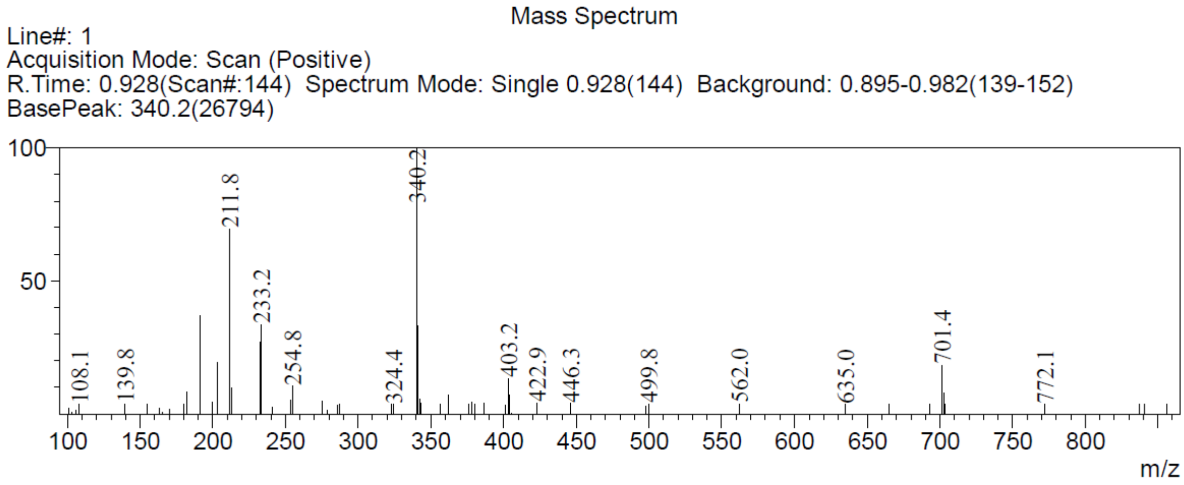

Compound 2h

Mass Spectrum

Line#: 1
Acquisition Mode: Scan (Positive)
R.Time: 0.988(Scan#:153) Spectrum Mode: Averaged 0.982-0.995(152-154)
BasePeak: 354.2(299579)

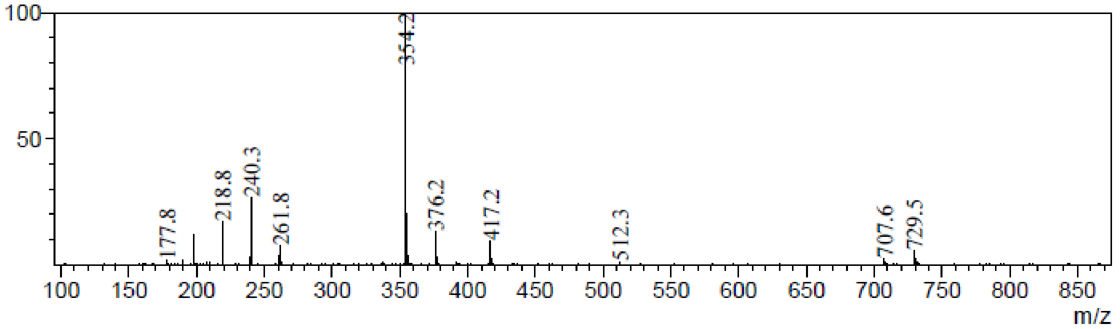

Compound 3a

Mass Spectrum

Line#: 1
Acquisition Mode: Scan (Positive)
R.Time: 0.682(Scan#:107) Spectrum Mode: Averaged 0.675-0.688(106-108)
BasePeak: 374.2(109697)

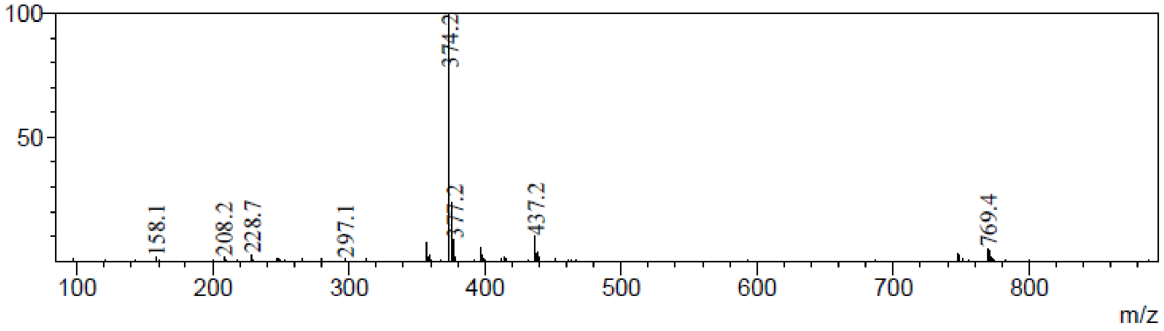

Compound 3b

Mass Spectrum

Line#: 1
Acquisition Mode: Scan (Positive)
R.Time: 2.048(Scan#:312) Spectrum Mode: Averaged 2.042-2.055(311-313)
BasePeak: 414.2(494777)

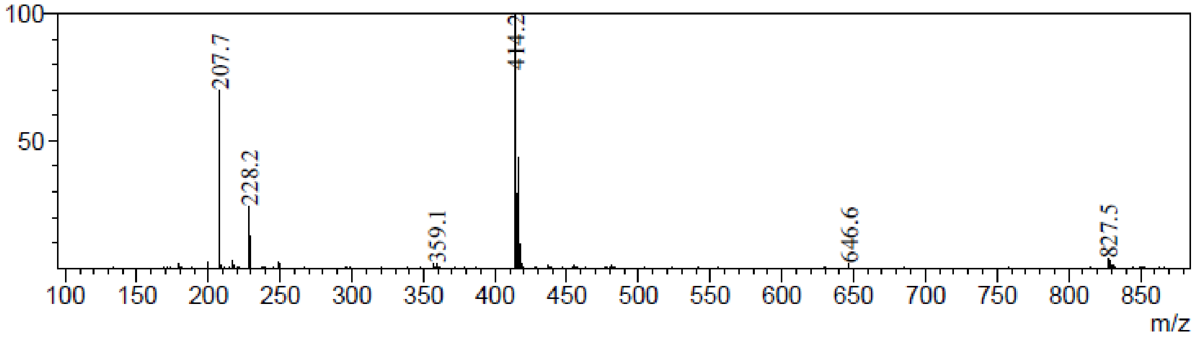

Compound 4/KMI169

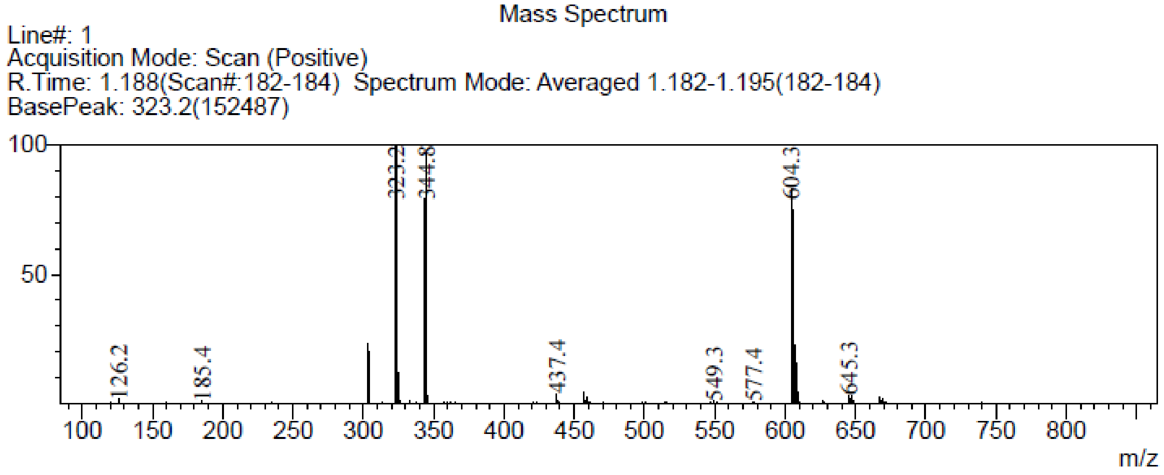

Compound KMI169Ctrl

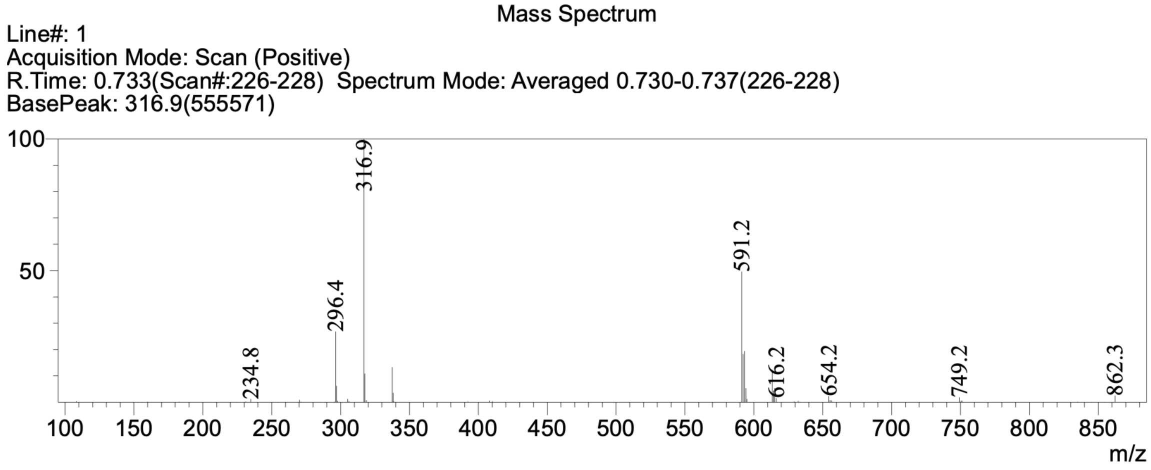

Supplement: Supplementary file 3 — Supplementary Dataset 1 [file 41467_2023_44243_MOESM3_ESM.pdf]
