# Supplementary material for: Structure-guided design of a selective inhibitor of the methyltransferase KMT9 with cellular activity
Source: Nat Commun. 2024 Jan 2;15:43. doi: 10.1038/s41467-023-44243-6 (PMC10762027; doi:10.1038/s41467-023-44243-6)
Supplement: Supplementary file 4 — Reporting Summary [file 41467_2023_44243_MOESM4_ESM.pdf]

nature portfolio

Corresponding author(s): Prof. Roland Schüle
Dr. Eric Metzger

Last updated by author(s): Oct 25, 2023

Reporting Summary

Nature Portfolio wishes to improve the reproducibility of the work that we publish. This form provides structure for consistency and transparency in reporting. For further information on Nature Portfolio policies, see our Editorial Policies and the Editorial Policy Checklist.

Statistics

For all statistical analyses, confirm that the following items are present in the figure legend, table legend, main text, or Methods section.

n/a	Confirmed	
☐	☒	The exact sample size (*n*) for each experimental group/condition, given as a discrete number and unit of measurement
☐	☒	A statement on whether measurements were taken from distinct samples or whether the same sample was measured repeatedly
☐	☒	The statistical test(s) used AND whether they are one- or two-sided<br>*Only common tests should be described solely by name; describe more complex techniques in the Methods section.*
☒	☐	A description of all covariates tested
☐	☒	A description of any assumptions or corrections, such as tests of normality and adjustment for multiple comparisons
☐	☒	A full description of the statistical parameters including central tendency (e.g. means) or other basic estimates (e.g. regression coefficient) AND variation (e.g. standard deviation) or associated estimates of uncertainty (e.g. confidence intervals)
☐	☒	For null hypothesis testing, the test statistic (e.g. *F*, *t*, *r*) with confidence intervals, effect sizes, degrees of freedom and *P* value noted<br>*Give P values as exact values whenever suitable.*
☒	☐	For Bayesian analysis, information on the choice of priors and Markov chain Monte Carlo settings
☒	☐	For hierarchical and complex designs, identification of the appropriate level for tests and full reporting of outcomes
☒	☐	Estimates of effect sizes (e.g. Cohen's *d*, Pearson's *r*), indicating how they were calculated

Our web collection on statistics for biologists contains articles on many of the points above.

Software and code

Policy information about availability of computer code

Data collection	Software of Illumina Hisiq200 pipeline was used to convert raw Illumina output to fastq format<br>NMR spectra: Bruker Avance 400 spectrometer with TopSpin<br>Monolith NT.115 instrument was used for MST data collection.<br>TriCarb 2910 TR (Perkin Elmer) scintillation counter was used for IC50 data collection.<br>CFX96 Touch Real-Time PCR Detection System (Bio-Rad Laboratories Inc.) was used for FTSA data collection.<br>DA+ data acquisition software at Swiss Light Source (SLS) PX3 beamline was used for crystallographic data collection.

Data analysis	Annotation: Refseq hg19 , Homer 4.11<br>Mapping: STAR version 2.7.10b<br>Heatmap: Morpheus (https://software.broadinstitute.org/morpheus/)<br>Statistics: Excel 15.30, GraphPad Prism 6.0 or 7.0, EdgeR 2.38.4<br>MST binding affinity analysis: MO. Affinity<br>Tm and IC50 analysis: GraphPad Prism 7.0<br>GI50 deltaTm analysis: GraphPad Prism 6.0<br>Quantification of chemoluminescent signals from Western blots: Amersham imager 600 1.2.0 software<br>crystallographic data analysis: XDS-20210323, CCP4-8.0.0005, coot-0.9.8.4<br>NMR spectra anaylsis: MestreNova-11.0.4-18998<br>structure modeling: Schrodinger suite 2019-1 (Schrodinger, NY)

For manuscripts utilizing custom algorithms or software that are central to the research but not yet described in published literature, software must be made available to editors and reviewers. We strongly encourage code deposition in a community repository (e.g. GitHub). See the Nature Portfolio guidelines for submitting code & software for further information.

Data

Policy information about availability of data

All manuscripts must include a data availability statement. This statement should provide the following information, where applicable:

- Accession codes, unique identifiers, or web links for publicly available datasets
- A description of any restrictions on data availability
- For clinical datasets or third party data, please ensure that the statement adheres to our policy

The authors declare that the data supporting the findings of this study are available within the article and its Supplementary Information files, or are available on request. Crystallographic data have been deposited at the Protein Data Bank under the PDB code 8CNC, 8QDG, and 8QDI. The RNAseq data are deposited under GSE235595. The accession codes of all other PDB coordinate files referenced in this study are: 6H1E and 2RFI. Source data are provided with this paper.

Human research participants

Policy information about studies involving human research participants and Sex and Gender in Research.

Reporting on sex and gender	n/a
Population characteristics	n/a
Recruitment	n/a
Ethics oversight	n/a

Note that full information on the approval of the study protocol must also be provided in the manuscript.

Field-specific reporting

Please select the one below that is the best fit for your research. If you are not sure, read the appropriate sections before making your selection.

[x] Life sciences [ ] Behavioural & social sciences [ ] Ecological, evolutionary & environmental sciences

For a reference copy of the document with all sections, see nature.com/documents/nr-reporting-summary-flat.pdf

Life sciences study design

All studies must disclose on these points even when the disclosure is negative.

Sample size	A sample size of three technical replicates per biological replicate and three or four biological replicates was used for statistical analysis
Data exclusions	No data were excluded from the analyses
Replication	To verify the reproducibility of the experimental findings, we repeated the experiments at least two times . Experiments were repeated by different investigators. All attempts at replication were successful.
Randomization	The experiments were not randomized
Blinding	Investigators were not blinded during experiments analysis and outcome assessment.

Reporting for specific materials, systems and methods

We require information from authors about some types of materials, experimental systems and methods used in many studies. Here, indicate whether each material, system or method listed is relevant to your study. If you are not sure if a list item applies to your research, read the appropriate section before selecting a response.

Materials & experimental systems

n/a	Involved in the study
☐	☒ Antibodies
☐	☒ Eukaryotic cell lines
☒	☐ Palaeontology and archaeology
☒	☐ Animals and other organisms
☒	☐ Clinical data
☒	☐ Dual use research of concern

Methods

n/a	Involved in the study
☒	☐ ChIP-seq
☒	☐ Flow cytometry
☒	☐ MRI-based neuroimaging

Antibodies

Antibodies used

The primary antibodies and dilutions used were: anti-KMT9a (#27630, lot 20062017, Schüle Lab); 1/1000
anti-GAPDH (#MAB374, lot 3688975, Millipore), 1/500
anti-symmetric dimethyl arginine motif (SDMA, #13222, lot 8, Cell Signaling); 1/1000
anti-histone H4 (#ab10158, lot GR322677-1, Abcam), 1/ 3000
anti-H4K12me1 (#27429, lot 27062017, Schüle Lab); 1/1000
anti-PRMT5 (#MBS9405987, lot 6018, MyBioSource); 1/1000
anti-Histone H3 (#ab1791,lot GR300976-1, Abcam), 1/5000
anti-H3K4me2 (#CS-35-100, lotA391-001, Diagenode), 1/750
anti-H3K9me2 (#07-441, lot 1463717, Millipore), 1/1000
anti-H4K20me1 (#39727, lot 21115004, Active Motif), 1/1000

Validation

-anti-KMT9a and anti-H4K12me1 antibody validation described in doi:10.1038/s41594-019-0219-9

The other antibodies were sourced from commercial suppliers. Additional details for validation and specificity for each antibody are available from the websites:
-anti-GAPDH: https://www.merckmillipore.com/DE/de/product/Anti-Glyceraldehyde-3-Phosphate-Dehydrogenase-Antibody-clone-6C5,MM_NF-MAB374?cid=BI-XX-BRC-A-NANT-ANTI-B096-1308#documentation
-anti-symmetric dimethyl arginine motif (SDMA): https://www.cellsignal.com/products/primary-antibodies/symmetric-di-methyl-arginine-motif-sdme-rg-multimab-rabbit-mab-mix/13222?_requestid=922437
-anti-histone H4: https://www.abcam.com/products/primary-antibodies/histone-h4-antibody-chip-grade-ab10158.html
-anti-PRMT5: https://www.mybiosource.com/polyclonal-human-antibody/prmt5/9405987
-anti-Histone H3: https://www.abcam.com/products/primary-antibodies/histone-h3-antibody-nuclear-marker-and-chip-grade-ab1791.html
-anti-H3K4me2: https://www.diagenode.com/en/p/h3k4me2-polyclonal-antibody-classic-100-ul
-anti-H3K9me2: https://www.merckmillipore.com/DE/de/product/Anti-dimethyl-Histone-H3-Lys9-Antibody,MM_NF-07-441?ReferrerURL=https%3A%2F%2Fwww.google.com%2F
-anti-H4K20me1: https://www.activemotif.com/catalog/details/39727

Eukaryotic cell lines

Policy information about cell lines and Sex and Gender in Research

Cell line source(s)

HepG2 (#HB-8065), 5637 (#HTB-9), UM-UC-3 (#CRL-1749), A549 (CRM-CCL-185), Caco-2 (HTB-37), PANC-1 (CRL-1469), DU145 (HTB-81), C4-2B (#CRL-3315), and 22Rv1 (#CRL-2505) cells were obtained from ATCC. CAL-29 (#ACC 515) cells were obtained from DSMZ. PC-3M (CVCL_5J25) and LNCaP (CVCL_5J24) cells were obtained from Caliper Life Scicence. LNCaP-abl (CVCL_4793) and LNCaP-abl-EnzaR cells were gifts from Z. Culig and have been previously described10. Cell lines were tested for mycoplasma and found to be uncontaminated. None of the used cell line is known as misidentified cell line by the International Cell Line Authentication Committee.

Authentication

None of the cell line used has been authenticated

Mycoplasma contamination

We confirm that all cell line were tested negative for mycloplasma contamination

Commonly misidentified lines
(See ICLAC register)

No cell line used is listed in the database of commonly missidentified cell lines
