# Supplementary material for: Structure-guided design of a selective inhibitor of the methyltransferase KMT9 with cellular activity
Source: Nat Commun. 2024 Jan 2;15:43. doi: 10.1038/s41467-023-44243-6 (PMC10762027; doi:10.1038/s41467-023-44243-6)
Supplement: Supplementary file 5 — Supplementary Dataset 2 [file 41467_2023_44243_MOESM5_ESM.pdf]

Structure-guided design of a selective inhibitor of the methyltransferase KMT9 with cellular activity

Supplementary Information

Table of Contents

Supplementary Figures 1-62 2-32

Supplementary Tables 1-2 33-34

Supplementary Methods 35-87

Supplementary Figures

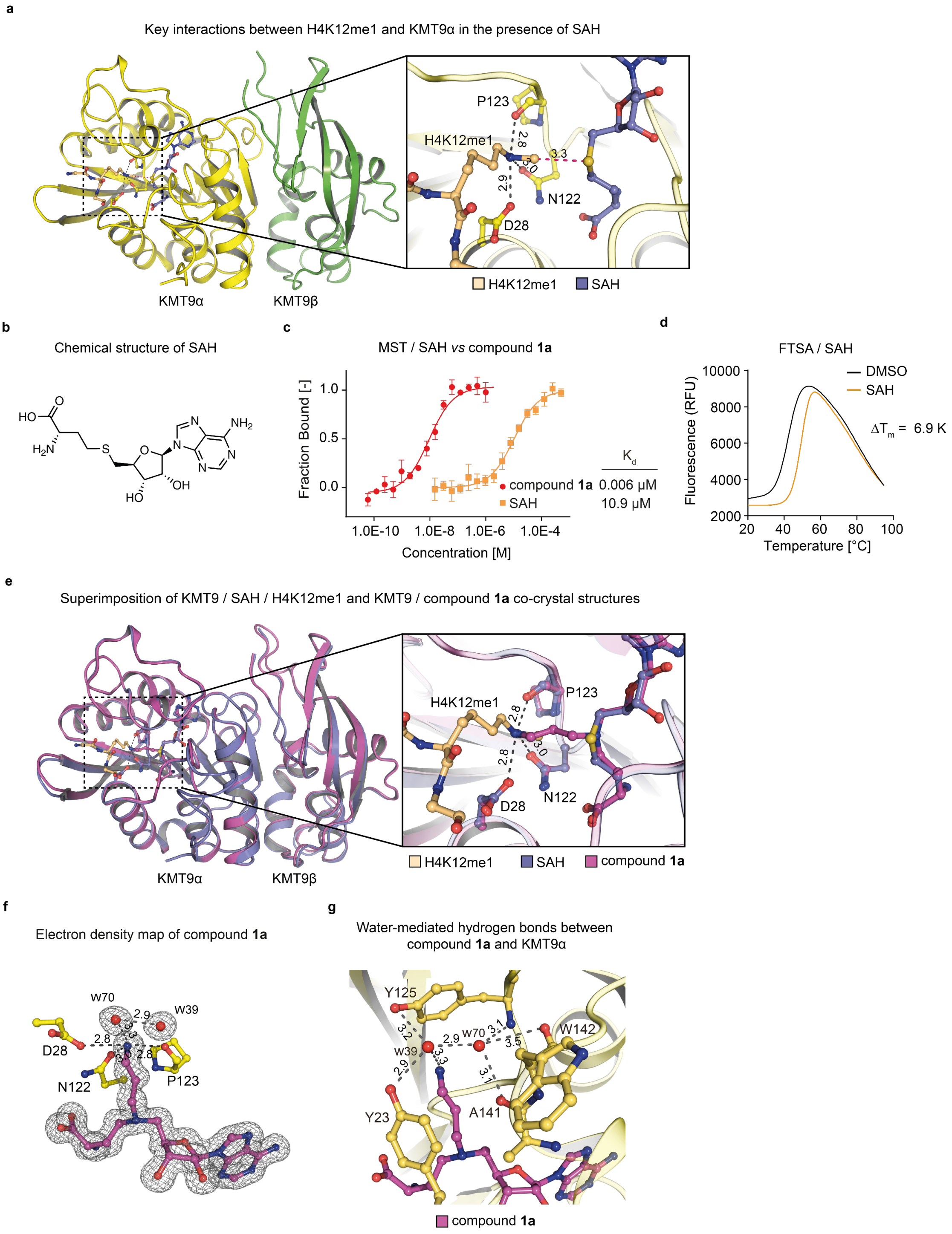

Supplementary Fig. 1. Identification of a bi-substrate inhibitor of KMT9. a, Crystal structure of the KMT9/SAH/H4K12me1 peptide complex (PDB code 6H1E). Key interactions between H4K12me1 (light orange) and KMT9α (yellow) in the presence of SAH (blue). **b**, Chemical structure of SAH. **c**, MST assay to determine the dissociation constant (K_d) of compound **1a** and SAH binding to KMT9. Data represent

means ±s.d (n=3 independent experiments). **d**, FTSA for SAH binding to KMT9. Data represent means (n=4 independent experiments). **e**, Superimposition of KMT9/SAH/H4K12me1 (grey) and KMT9/compound **1a** (magenta) crystal structures. KMT9α key residues, H4K12me1, SAH, and compound **1a** are shown as coloured sticks. **f**, *2Fo-Fc* omit electron density map contoured at 1σ for compound **1a**. **g**, Water-mediated hydrogen bonding network between the amino group of compound **1a** (magenta) and KMT9α (yellow). KMT9 protein is shown as ribbon (**a**, **e**, **g**). H4K12me1 residues (**a**, **e**) and ligands (**a, e- g**) are represented as sticks. Water molecules (**f**, **g**) are shown as red spheres.

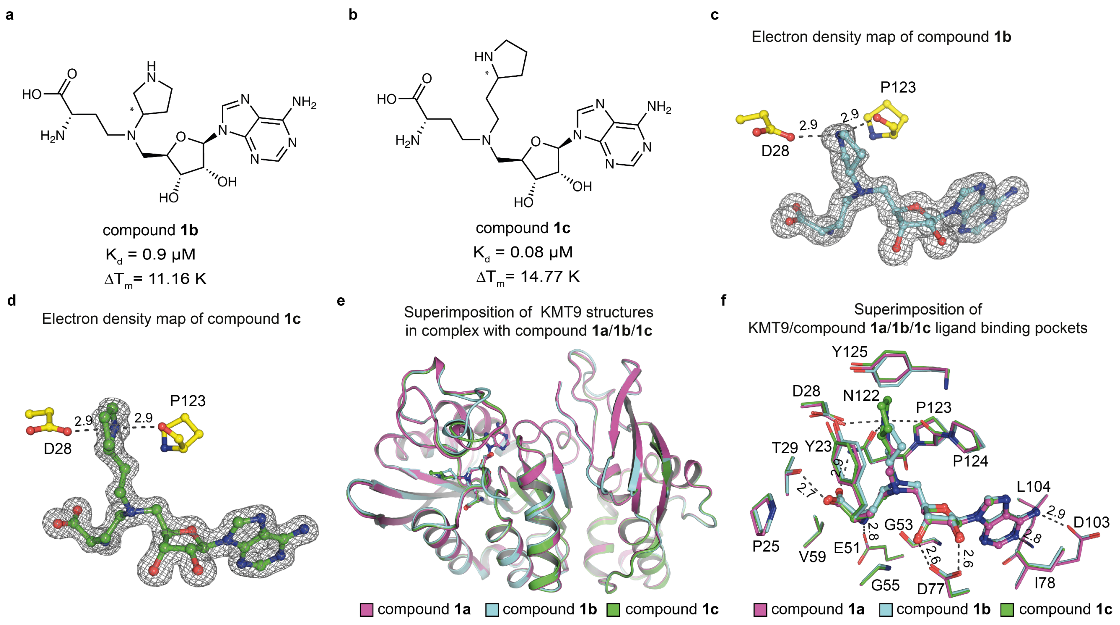

Supplementary Fig. 2. Structure of compound 1 derivatives. **a**, **b**, Chemical structures of compounds **1b** (**a**) and **1c** (**b**). K_d and ΔT_m values for each compound are listed. **c**, **d**, *2Fo-Fc* omit electron density maps contoured at 1σ for compounds **1b** (**c**, PDB code 8QDG) and **1c** (**d**, PDB code 8QDI). **e**, Superimposition of KMT9 crystal structures in complex with compound **1a** (magenta), **1b** (cyan), or **1c** (green). **f**, Superimposition of residues forming the binding pockets in KMT9 co-crystal structures of compound **1a** (magenta), **1b** (cyan), or **1c** (green). Compound **1a** (**e**, **f**, magenta), compound **1b** (**c**, **e**, **f**, cyan), compound **1c** (**d**, **e**, **f**, green) and key residues of KMT9α (**c, d**, yellow) are shown as sticks. Residues forming the KMT9 binding pockets of compounds 1a (magenta), 1b (cyan), or 1c (green) are shown as lines.

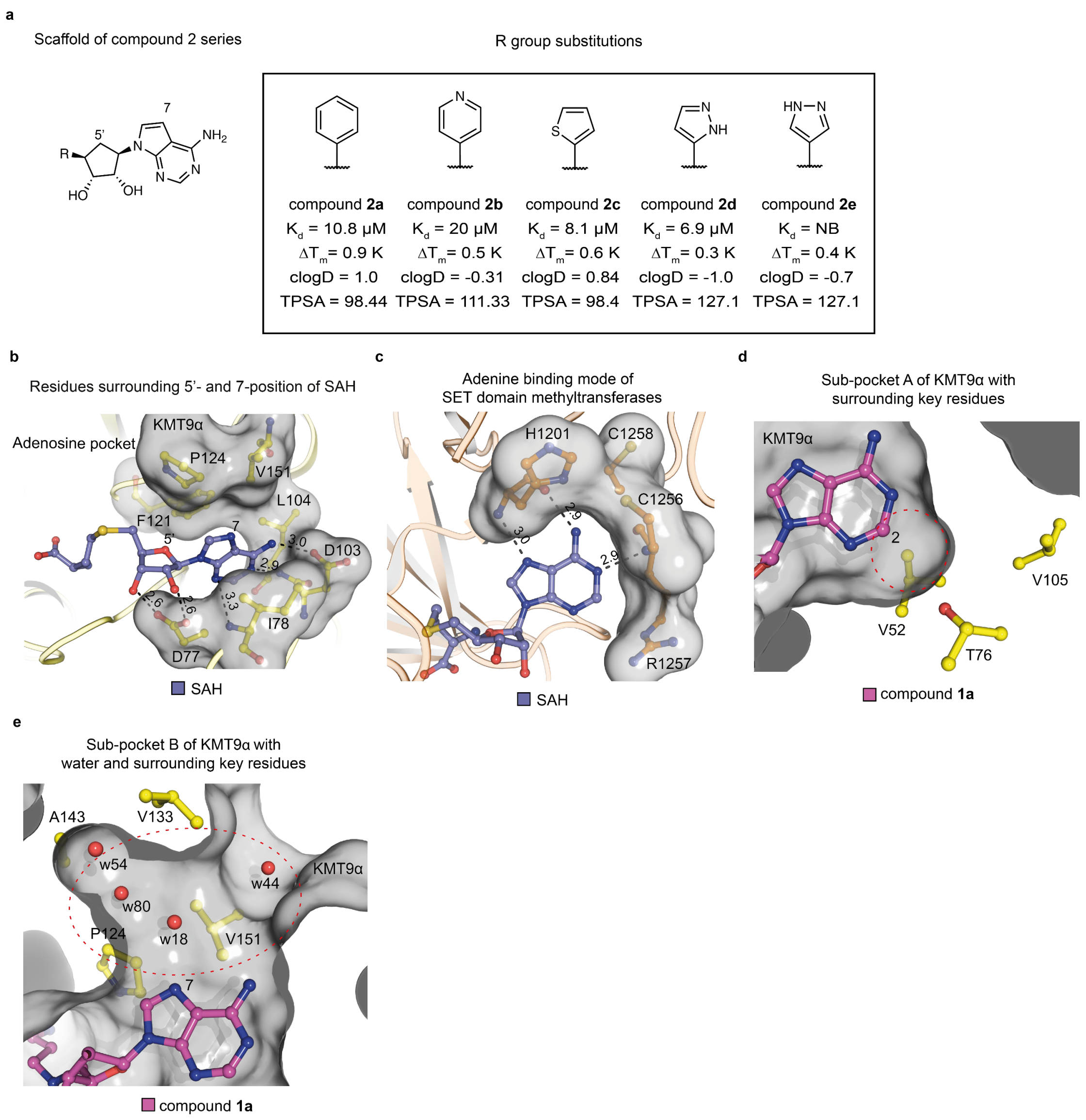

Supplementary Fig. 3. Structure-guided lead optimization of KMT9 inhibitors. **a**, Chemical structures of the compound **2** series. The scaffold structure (left) and the R group substitutions (right) for compounds **2a-e** are represented. Measured K_d and ΔT_m values and calculated clogD and TPSA values for each compound are listed. NB: no binding. **b**, Key residues in the adenosine pocket of KMT9α surrounding the 5'- and 7-position of the SAH moiety. **c**, Binding mode of the adenine moiety of SAH in SET domain containing PMTs (PDB code 2RFI). **d**, **e**, Sub-pocket A (**d**) and sub-pocket B (**e**) of KMT9α with key residues surrounding compound **1a**. SAH (**b**, **c**, light blue), compound **1a** (**d**, **e**, magenta) and key residues of KMT9α (**b**, **d**, **e**, yellow) or the SET domain (**c**, orange) are shown as sticks. The KMT9α protein pockets (**b**, **d**, **e**) and the

SET domain (**c**) are depicted as surface. Water molecules (**e**) are shown as red spheres. Hydrogen bonds (**b**, **c**) are depicted as grey dashed lines.

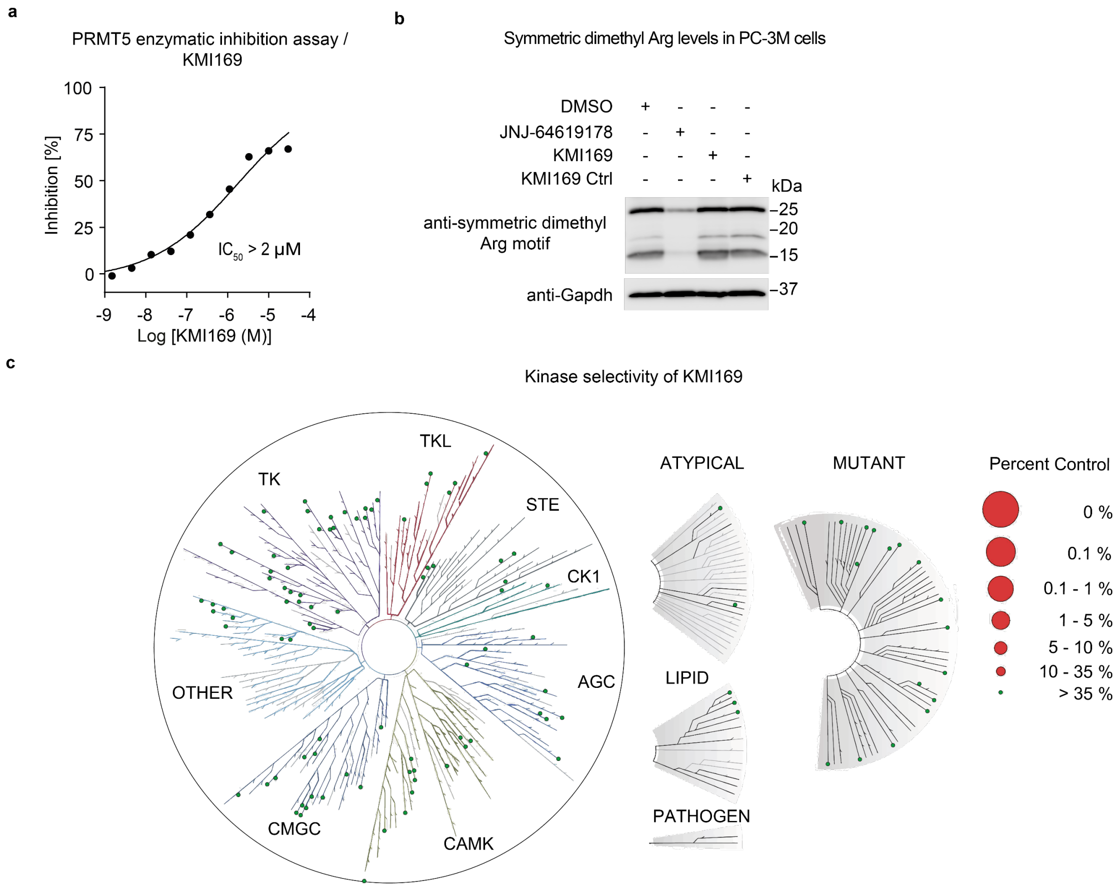

Supplementary Fig. 4. Characterization of compound 4 (KMI169). **a**, Enzymatic inhibition assay for PRMT5. At the highest KMI169 concentration (30 µM), the maximum inhibition was only about 60% allowing only to calculate an approximate IC_{50} value of >2 µM. Data represent means (n=2 independent experiments) **b**, Levels of symmetric dimethyl arginine in PC-3M cells treated with the PRMT5 inhibitor JNJ-64619178 (50 nM), KMI169 (360 nM), or KMI169Ctrl (2µM) for three days were analysed by Western blot with the indicated antibodies. GAPDH served as control. **c**, Selectivity of KMI169 against representative kinases.

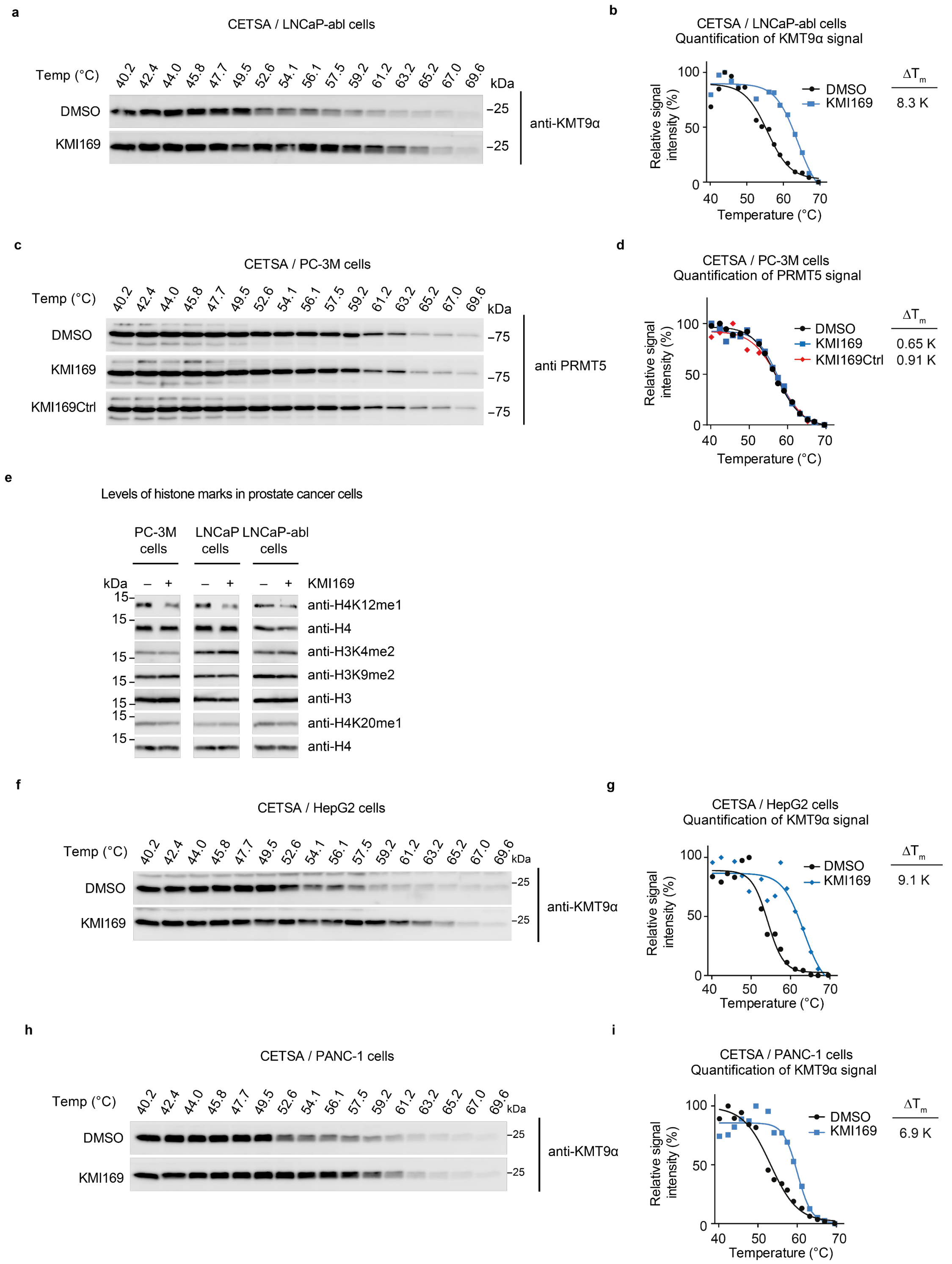

Supplementary Fig. 5. Cellular target engagement of KMI169. a, **b**, CETSA for KMT9 in LNCaP-abl cells treated with vehicle (DMSO) or 1 µM KMI169. Representative Western blots (**a**) and quantification (**b**) showing increased melting temperatures (ΔT_m) of endogenous KMT9 upon treatment with KMI169 compared to DMSO. **c, d,** CETSA for PRMT5 in PC-3M cells treated with DMSO, 1 µM KMI169,

or 1 μM KMI169Ctrl. Representative Western blots (**c**) and quantification (**d**) showing unchanged melting temperatures (ΔT_m) of endogenous PRMT5 upon treatment with KMI169 compared to KMI169Ctrl or DMSO. **e**, Levels of H4K12me1, H3K4me2, H3K9me2, and H4K20me1 in PC-3M, LNCaP, and LNCaP-abl prostate tumour cells cultured in the presence of DMSO (-) or 500 nM KMI169 (+) for three days were analysed by Western blot. Western blots were decorated with the indicated antibodies. Histones H3 and H4 served as controls. **f-i**, CETSA for HepG2 (**f**, **g**) and PANC-1 cells (**h**, **i**) treated with DMSO or 1 μM KMI169. Representative Western blots (**f**, **h**) and quantification (**g**, **i**) showing increased melting temperatures (ΔT_m) of endogenous KMT9 upon treatment with KMI169 compared to DMSO.

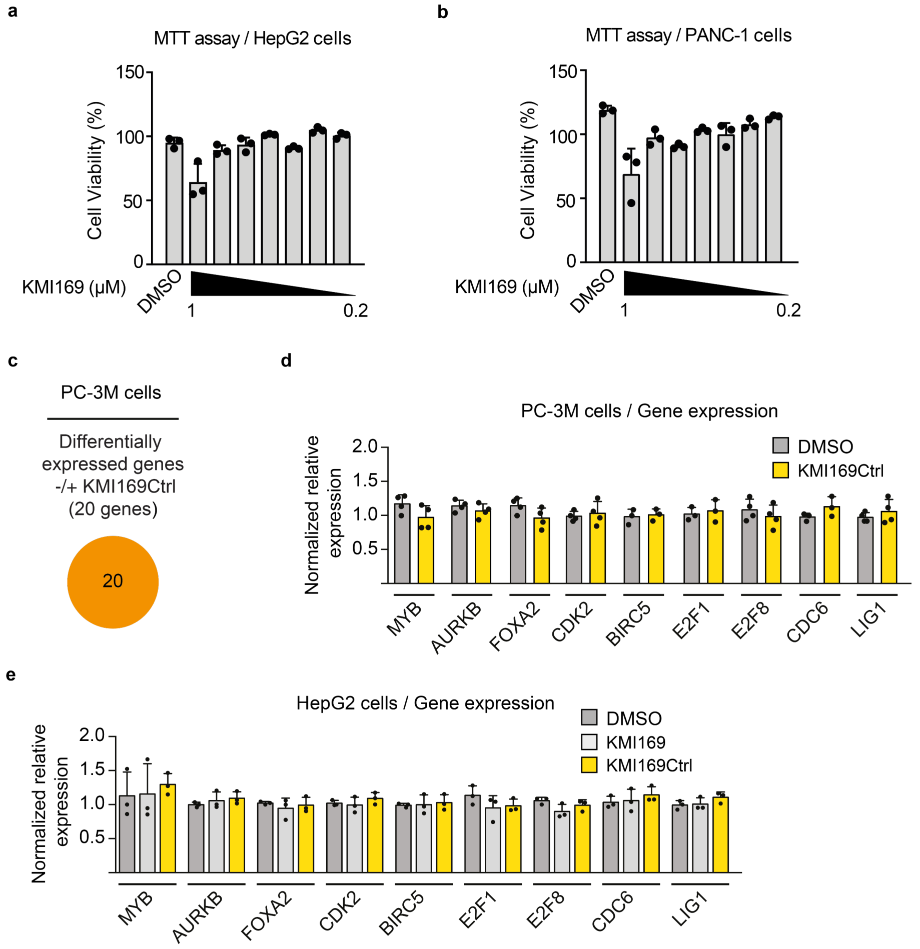

Supplementary Fig. 6. Inhibition of KMT9 impairs tumour cell proliferation.

a, **b**, Relative viability of HepG2 (**a**) and PANC-1 (**b**) cells treated with KMI169 at the indicated concentrations. **c**, Diagram showing the number of differentially expressed genes in PC-3M cells upon four days of treatment with 360 nM KMI169Ctrl. **d**, QRT-PCR analysis showing relative mRNA levels of the indicated genes in PC-3M cells cultured for four days in the presence of DMSO or 360 nM KMI169Ctrl. Data represent means +s.d (n=3 biologically independent samples). **e**, QRT-PCR analysis showing relative mRNA levels of the indicated genes in HepG2 cells cultured for four days in the presence of DMSO, KMI169 (360 nM), or KMI169Ctrl (360 nM). Data represent means +s.d (n=3 biologically independent samples).

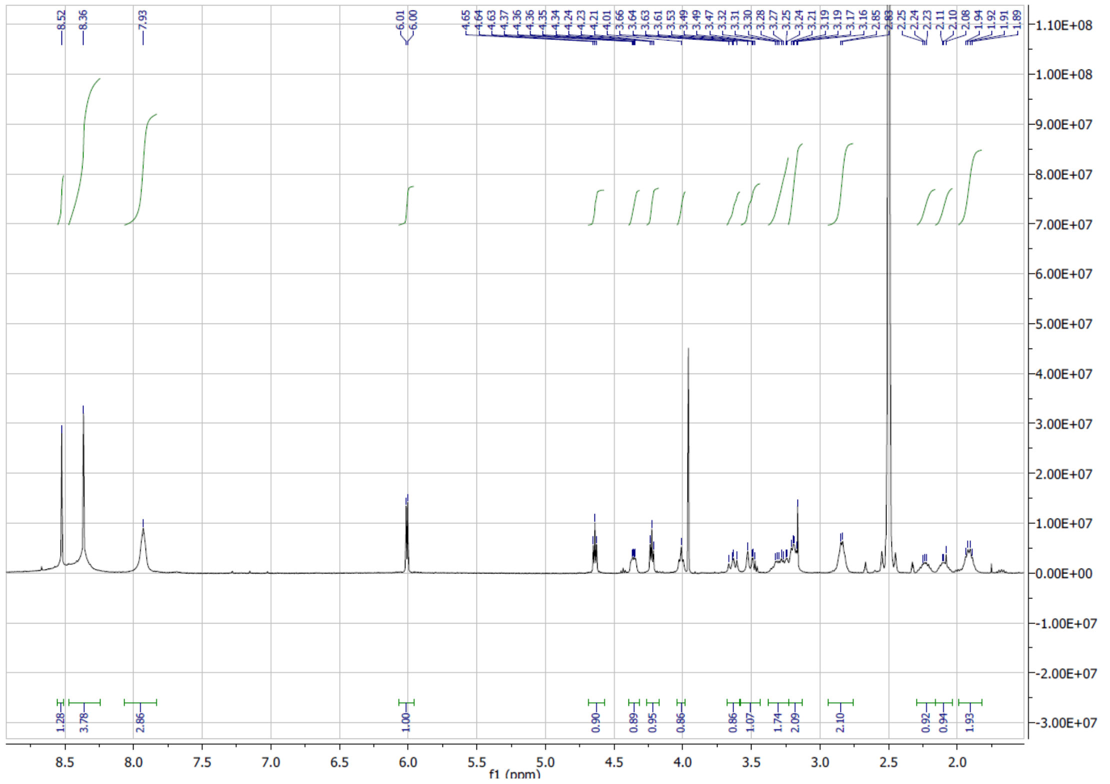

Supplementary Fig. 7. ^{1}H-NMR spectrum of compound 1a (DMSO d6, 400 MHz).

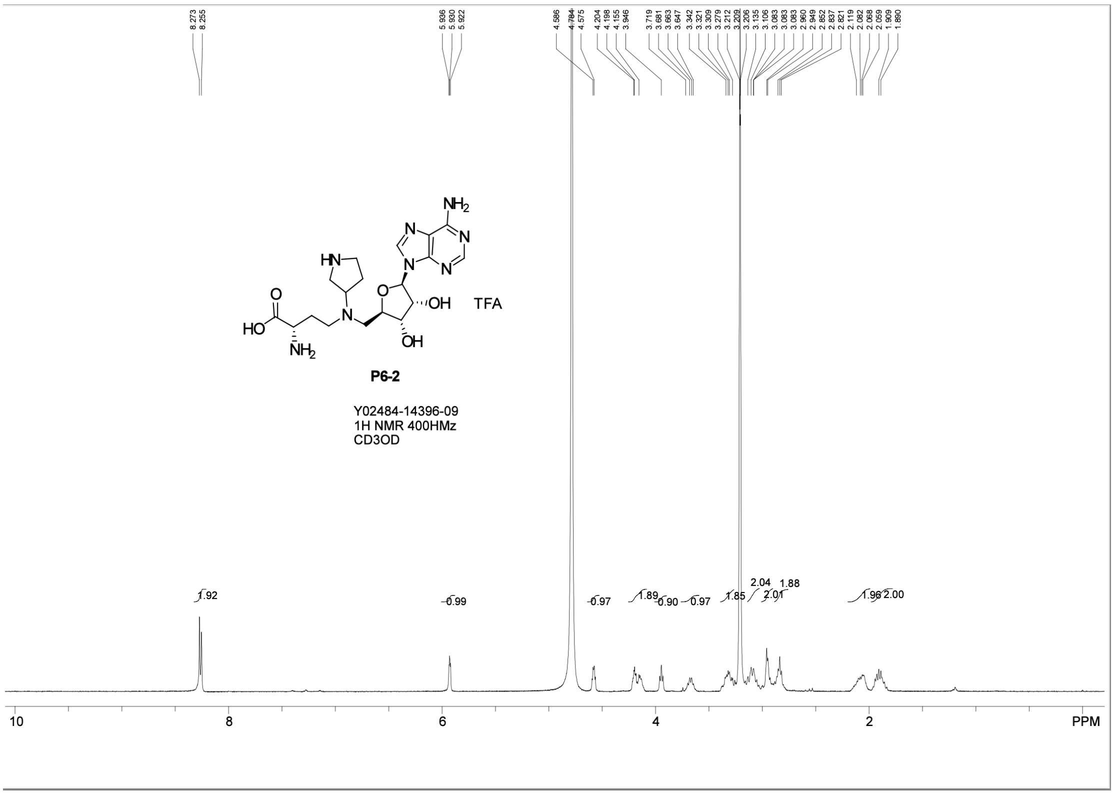

Supplementary Fig. 8. ^{1}H-NMR spectrum of compound 2b (MeOD, 400 MHz).

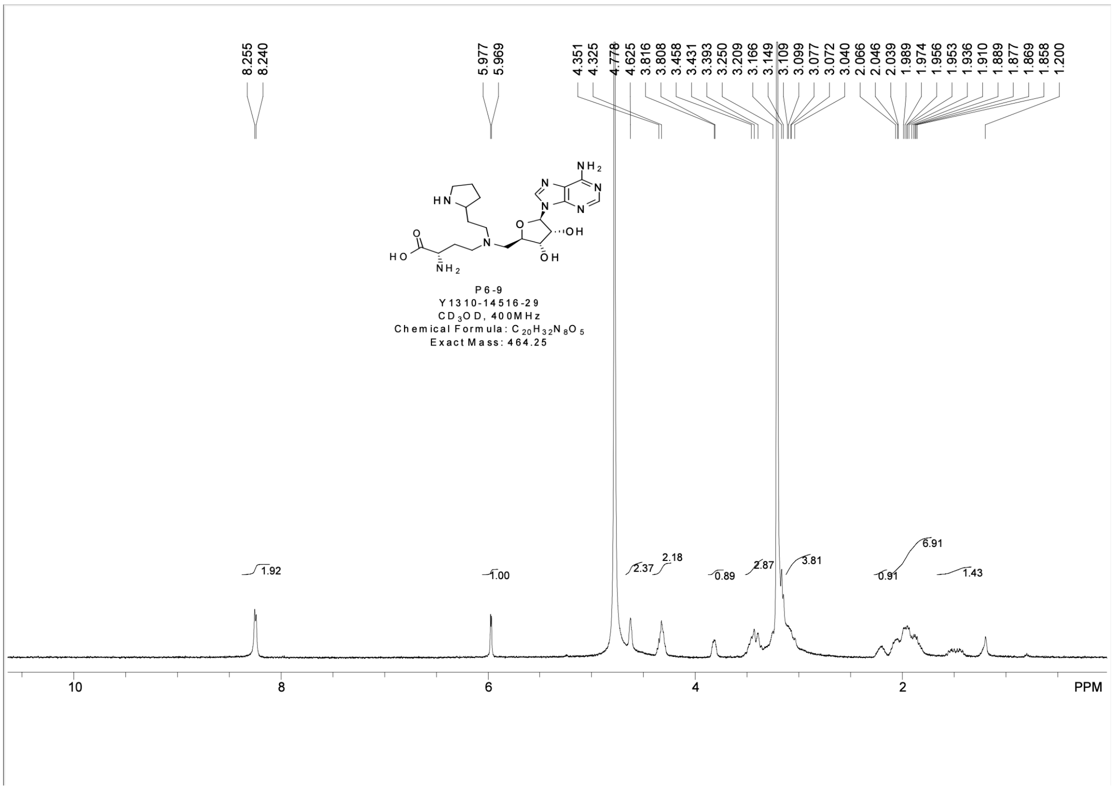

Supplementary Fig. 9. ^{1}H-NMR spectrum of compound 1c (MeOD, 400 MHz).

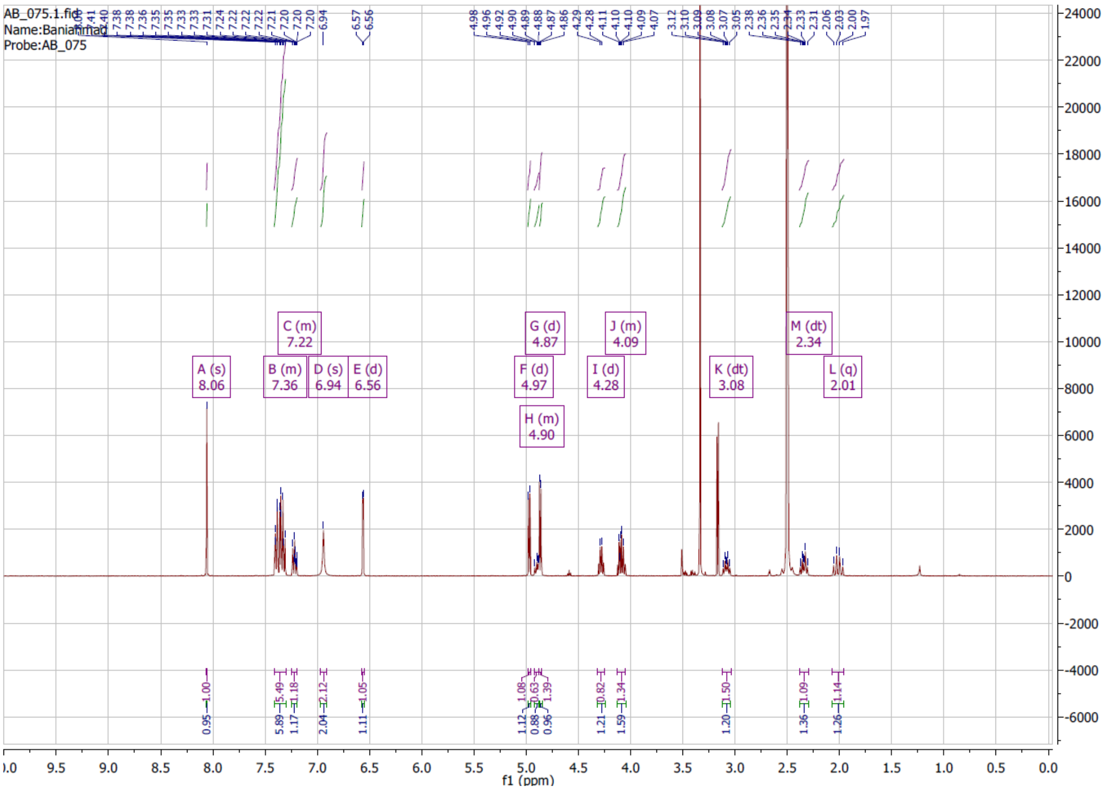

Supplementary Fig. 10. ^{1}H-NMR spectrum of compound 2a (DMSO d6, 400 MHz).

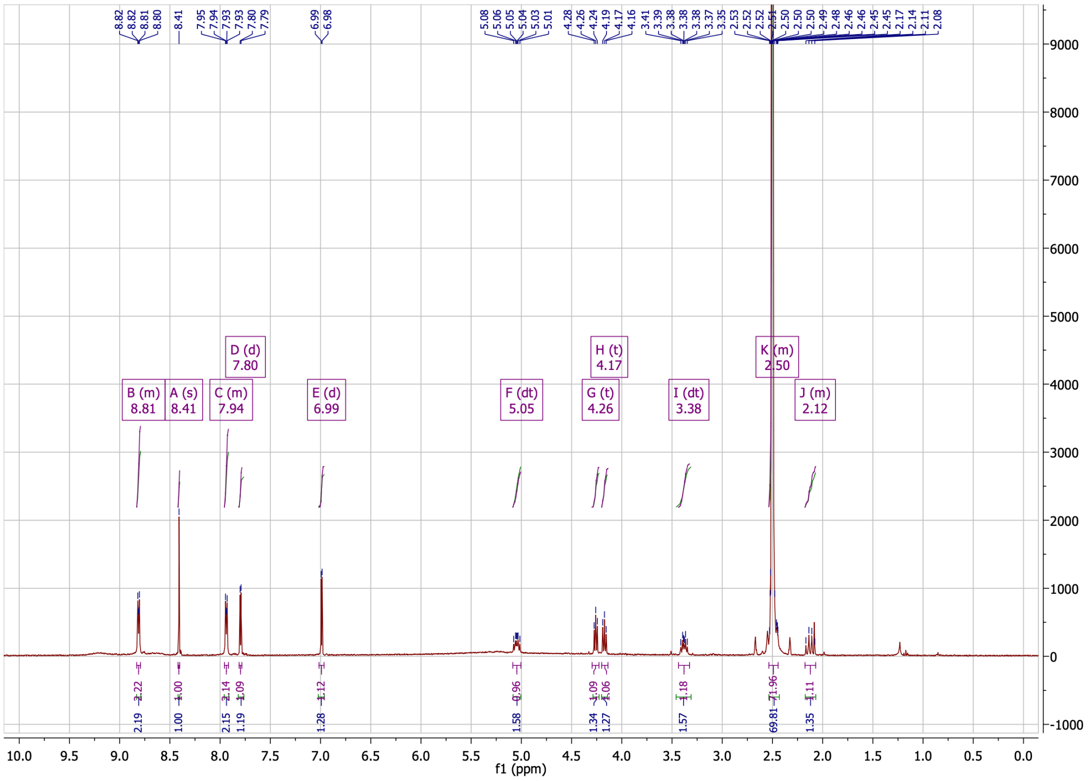

Supplementary Fig. 11. ^{1}H-NMR spectrum of compound 2b (DMSO d6, 400 MHz).

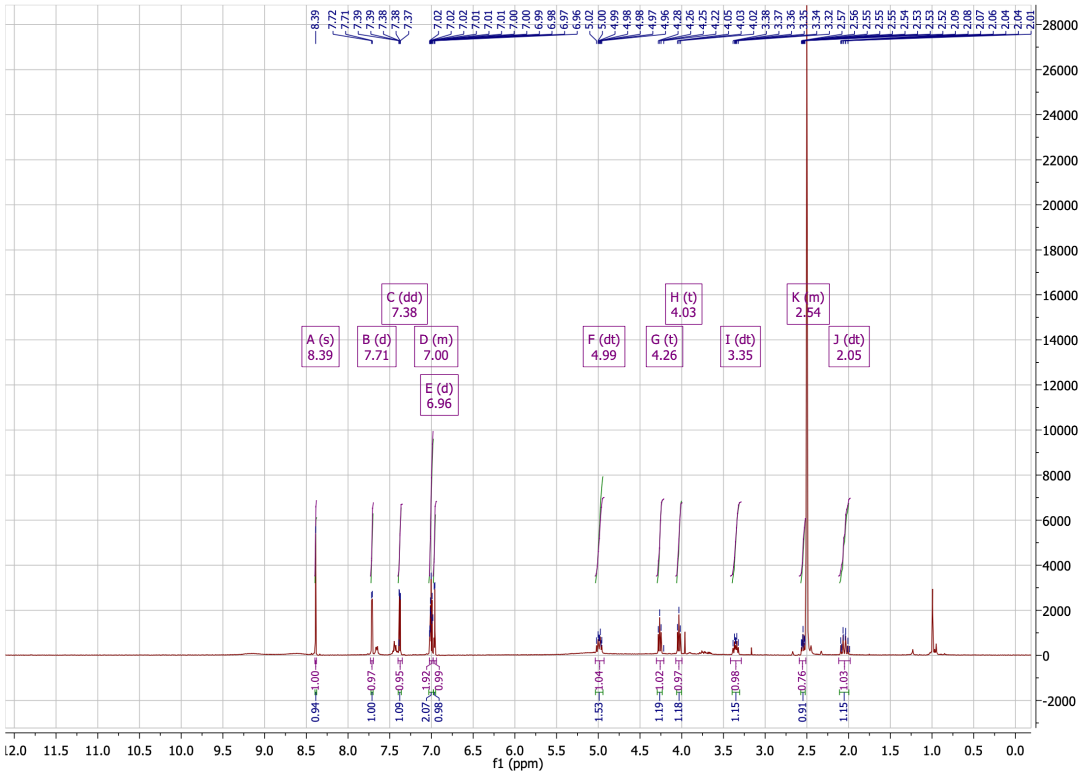

Supplementary Fig. 12. ^{1}H-NMR spectrum of compound 2c (DMSO d6, 400 MHz).

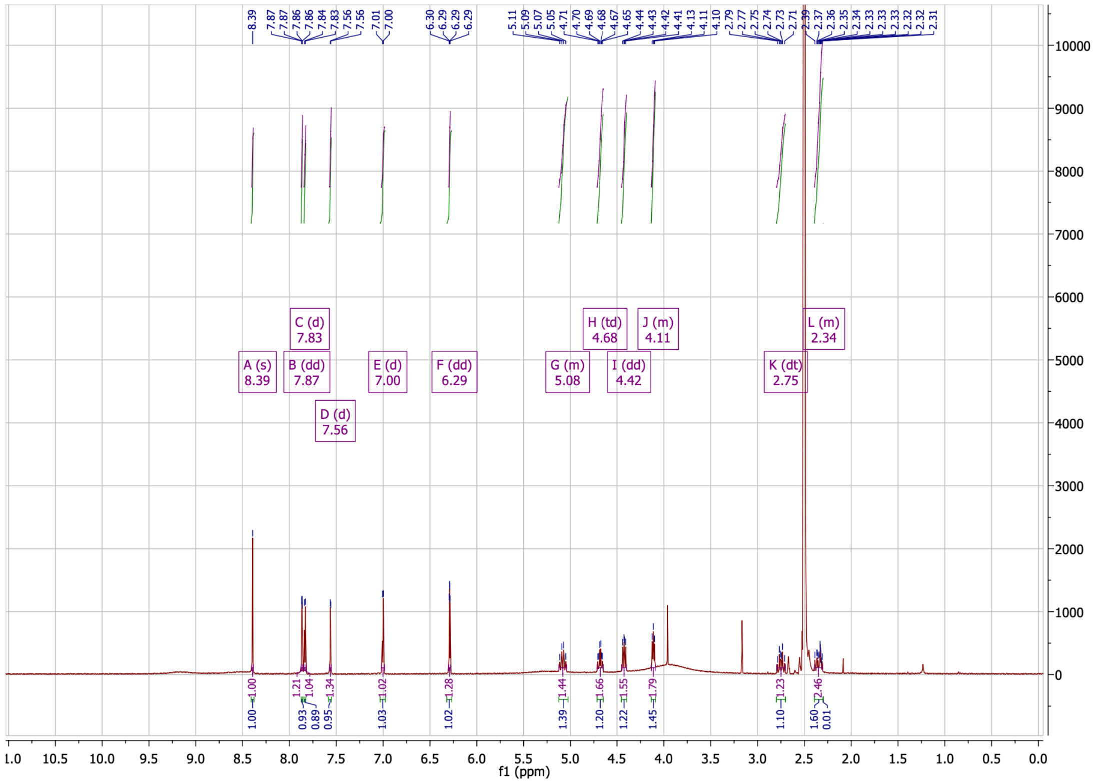

Supplementary Fig. 13. ^{1}**H-NMR spectrum of compound 2d (DMSO d6, 400 MHz).**

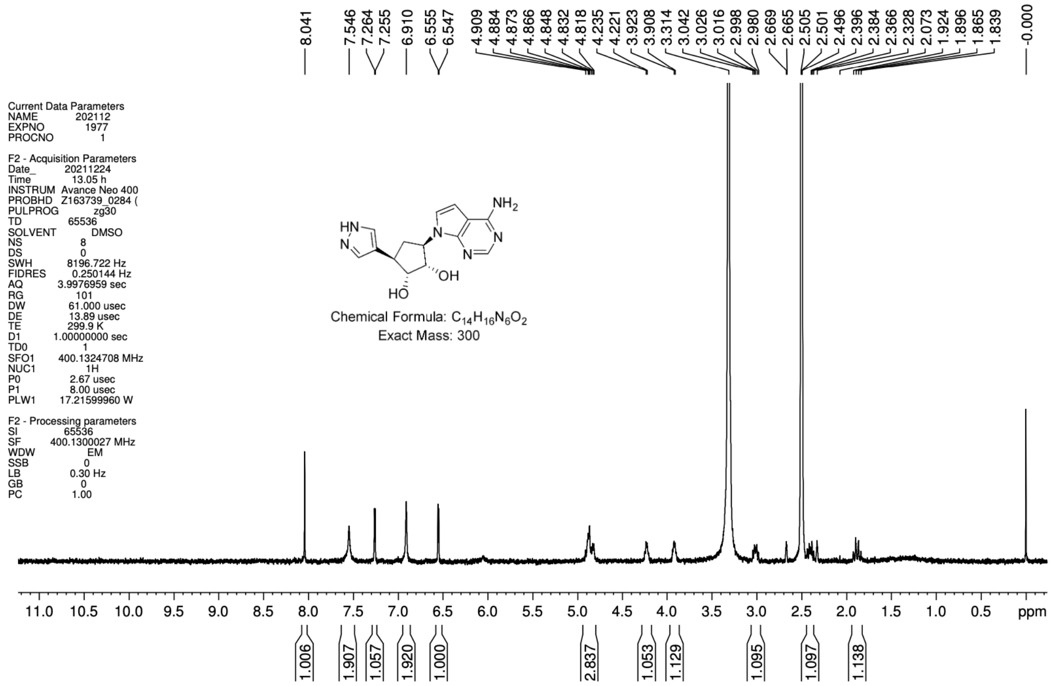

Supplementary Fig. 14. ^{1}**H-NMR spectrum of compound 2e (DMSO d6, 400 MHz).**

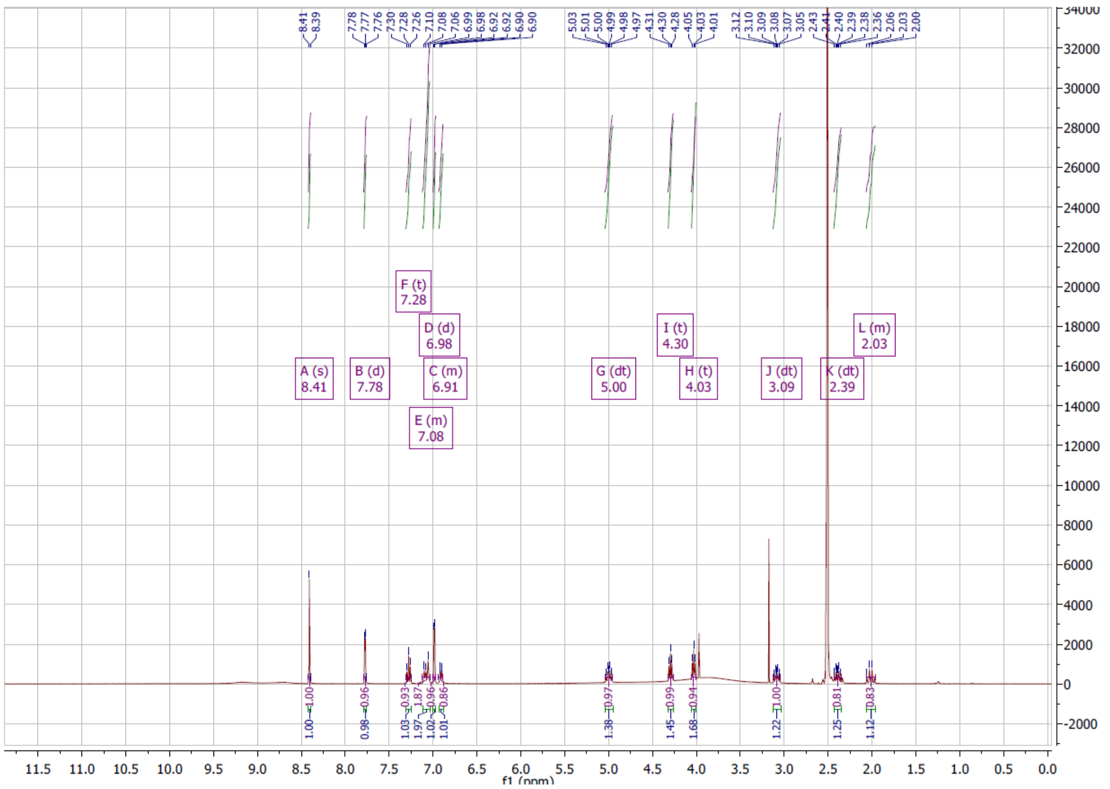

Supplementary Fig. 15. ^{1}**H-NMR spectrum of compound 2f (DMSO d6, 400 MHz).**

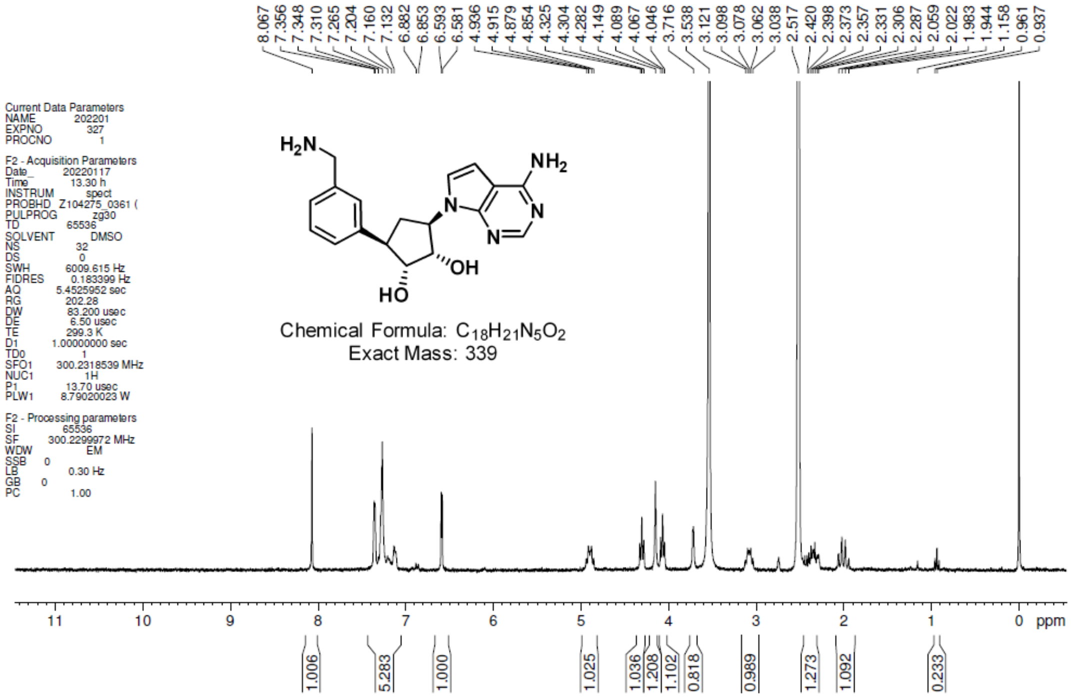

Supplementary Fig. 16. ^{1}**H-NMR spectrum of compound 2g (DMSO d6, 400 MHz).**

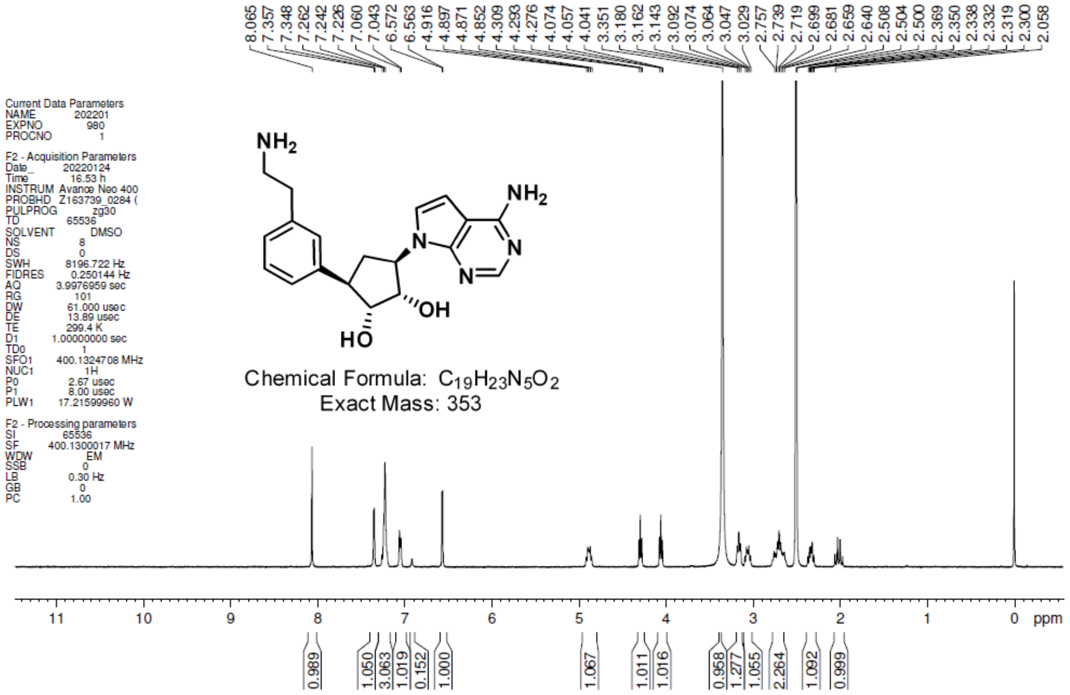

Supplementary Fig. 17. ^{1}H-NMR spectrum of compound 2h (DMSO d6, 400 MHz).

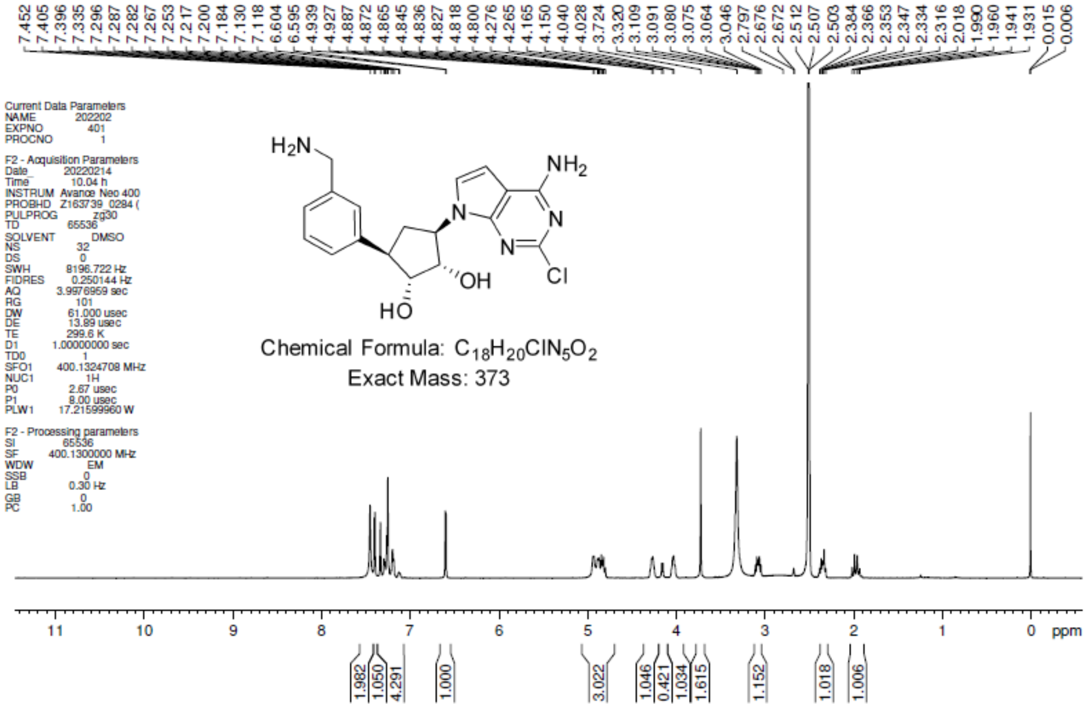

Supplementary Fig. 18. ^{1}H-NMR spectrum of compound 3a (DMSO d6, 400 MHz).

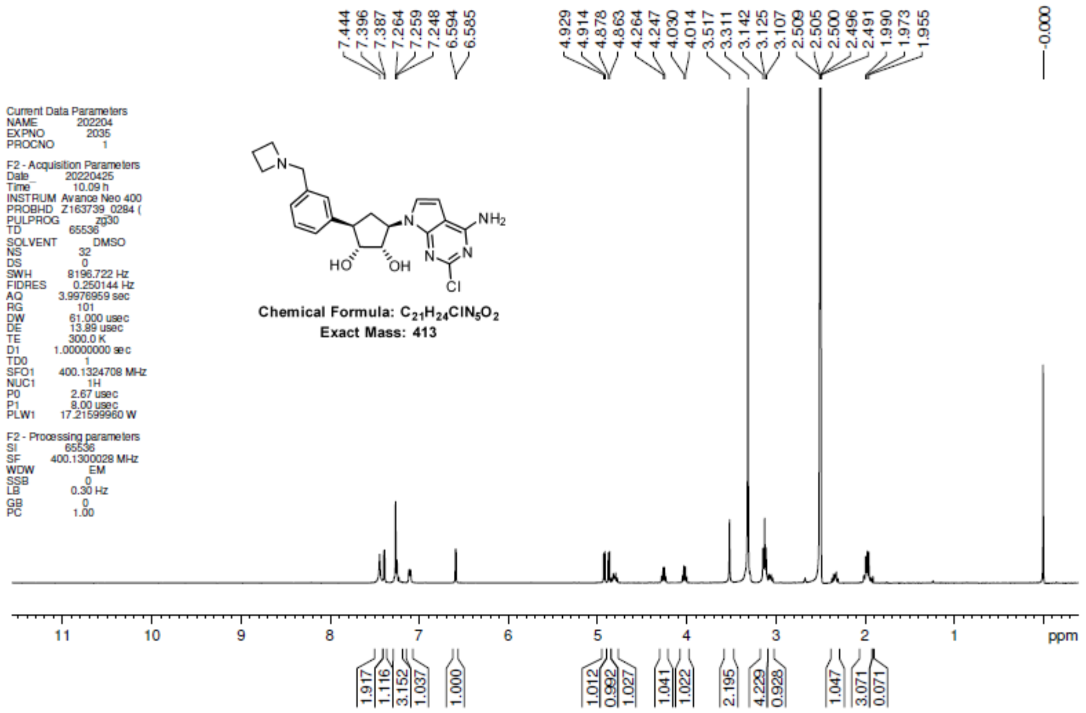

Supplementary Fig. 19. ^{1}H-NMR spectrum of compound 3b (DMSO d6, 400 MHz).

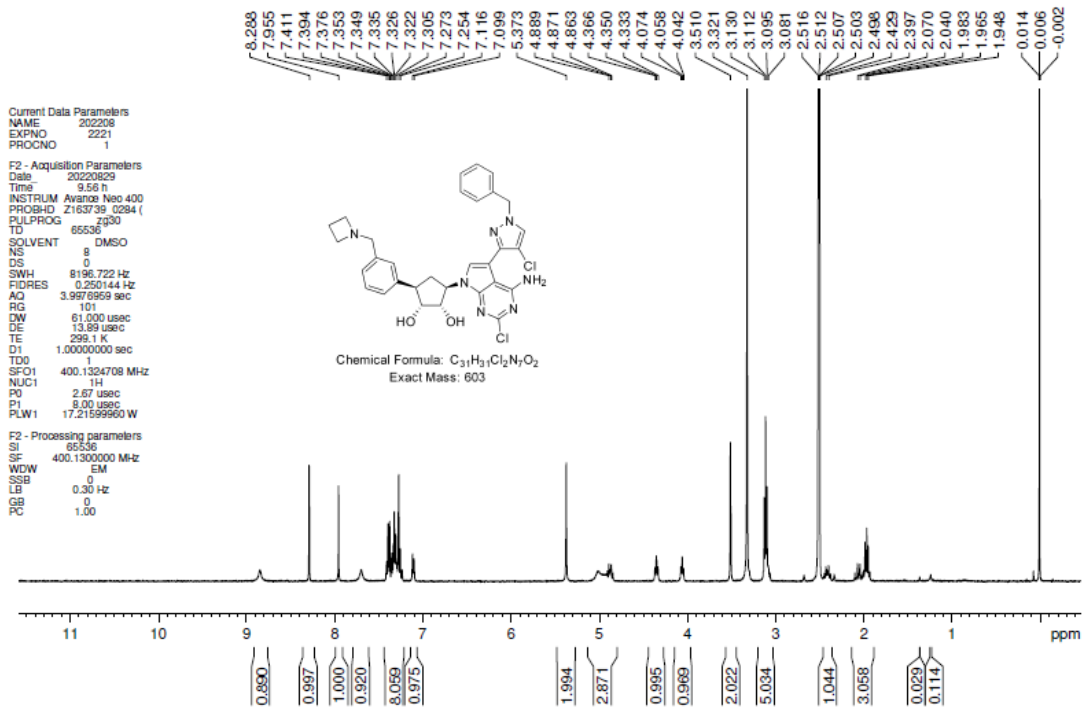

Supplementary Fig. 20. ^{1}H-NMR spectrum of compound 4 (DMSO d6, 400 MHz).

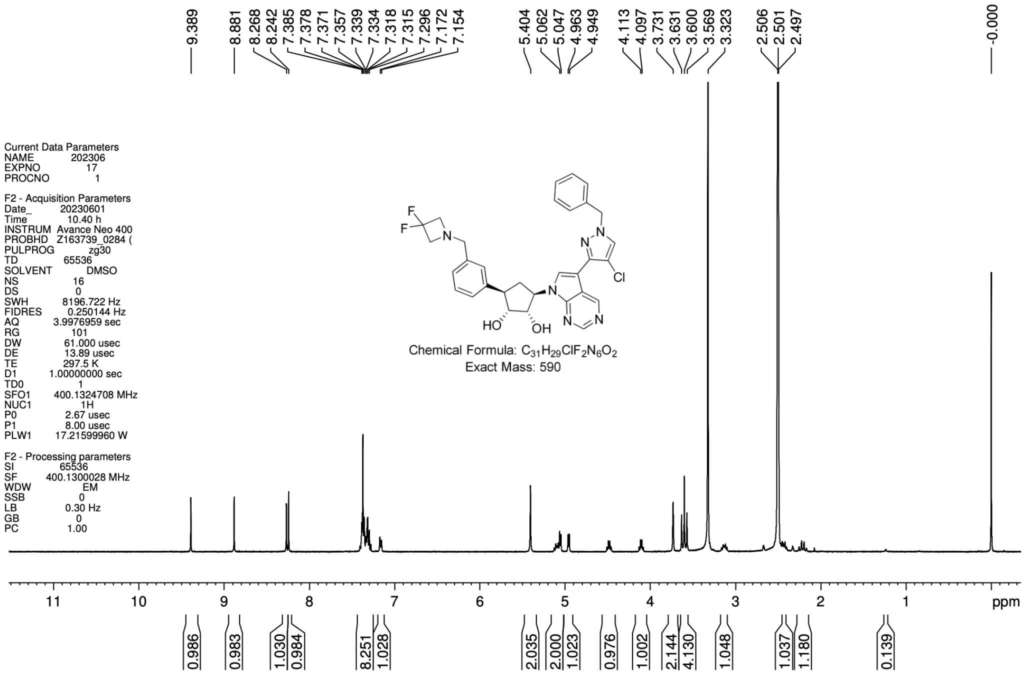

Supplementary Fig. 21. ^{1}**H-NMR spectrum of compound KMI169Ctrl (DMSO d6, 400 MHz).**

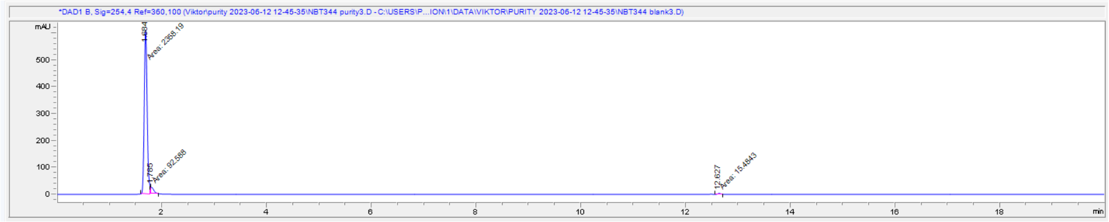

Supplementary Fig. 22. HPLC chromatogram of compound 1a

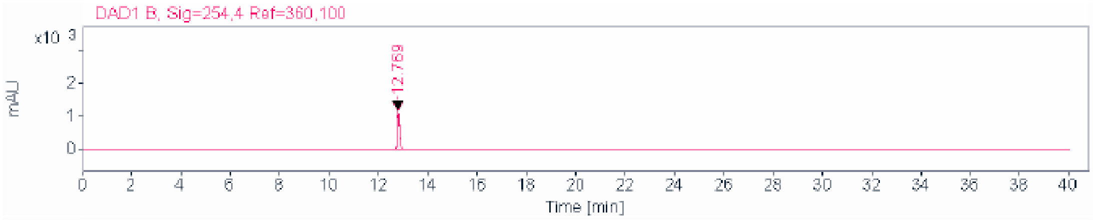

Supplementary Fig. 23. HPLC chromatogram of compound 2a

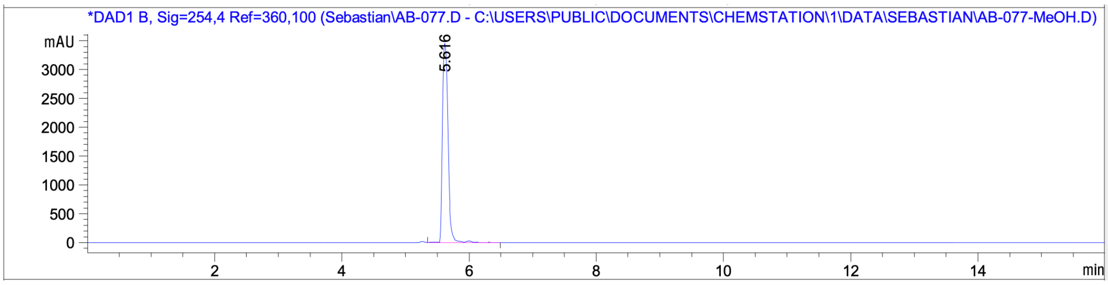

Supplementary Fig. 24. HPLC chromatogram of compound 2b

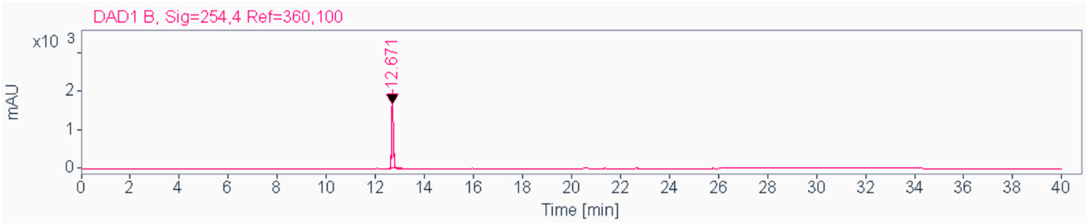

Supplementary Fig. 25. HPLC chromatogram of compound 2c

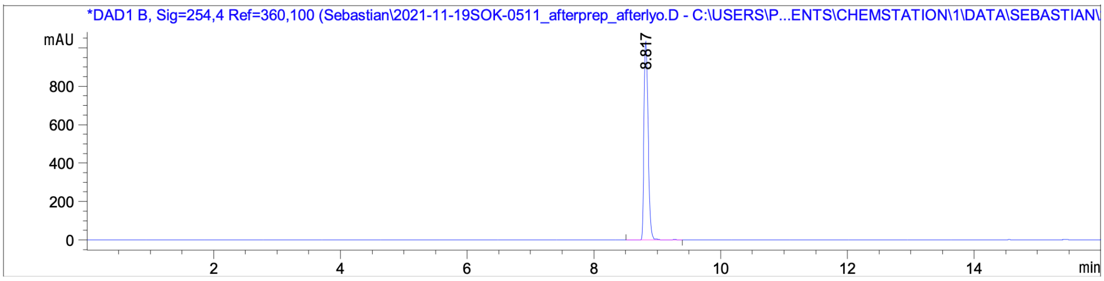

Supplementary Fig. 26. HPLC chromatogram of compound 2d

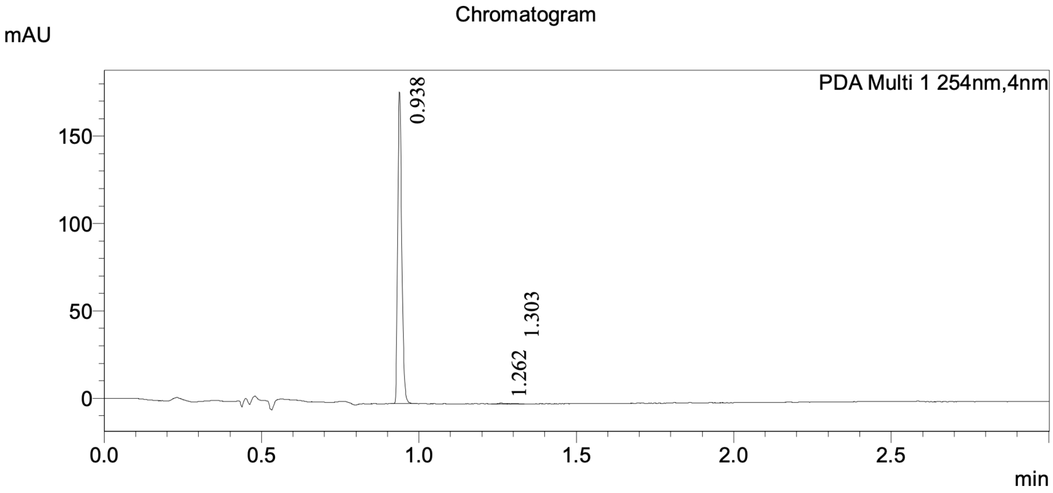

Peak Table

PDA Ch1 254nm

Peak#	Ret. Time	Height	Height%	Area	Area%
1	0.938	178169	99.540	160053	99.327
2	1.262	451	0.252	586	0.364
3	1.303	372	0.208	497	0.309
Total		178993	100.000	161136	100.000

Supplementary Fig. 27. HPLC chromatogram of compound 2e

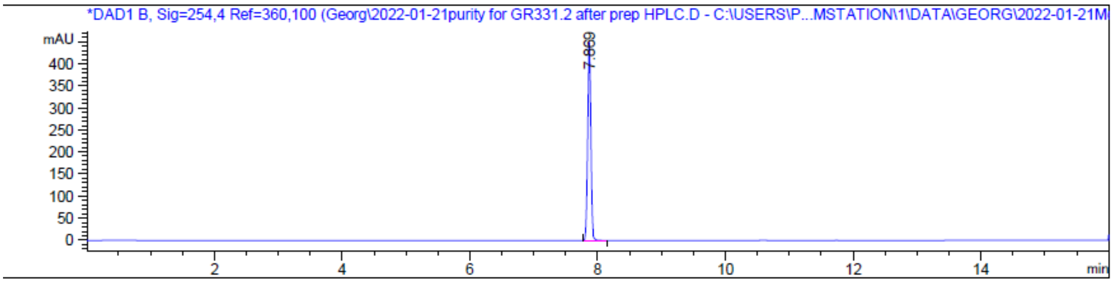

Supplementary Fig. 28. HPLC chromatogram of compound 2f

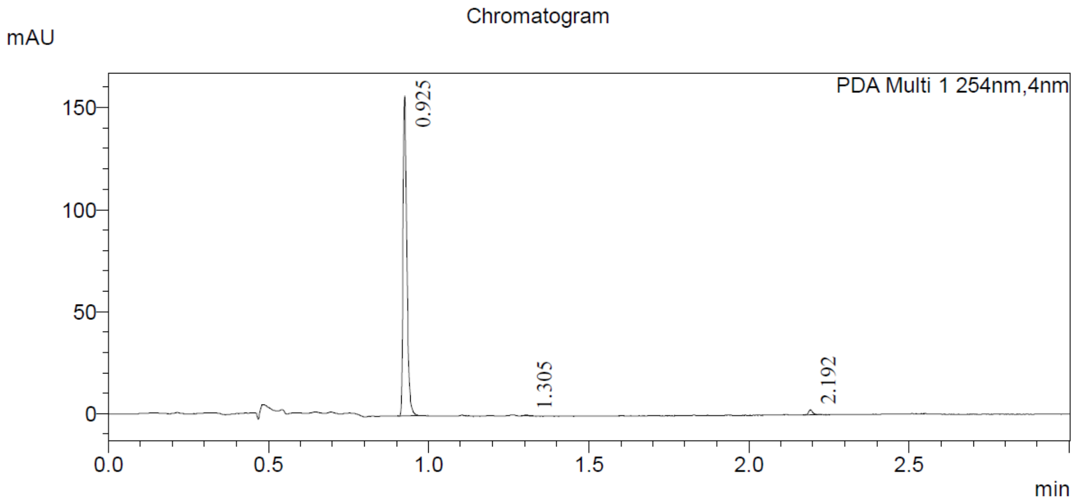

Peak Table

PDA Ch1 254nm

Peak#	Ret. Time	Height	Height%	Area	Area%
1	0.925	155388	98.116	128629	97.778
2	1.305	582	0.367	596	0.453
3	2.192	2402	1.517	2327	1.769
Total		158372	100.000	131553	100.000

Supplementary Fig. 29. HPLC chromatogram of compound 2g

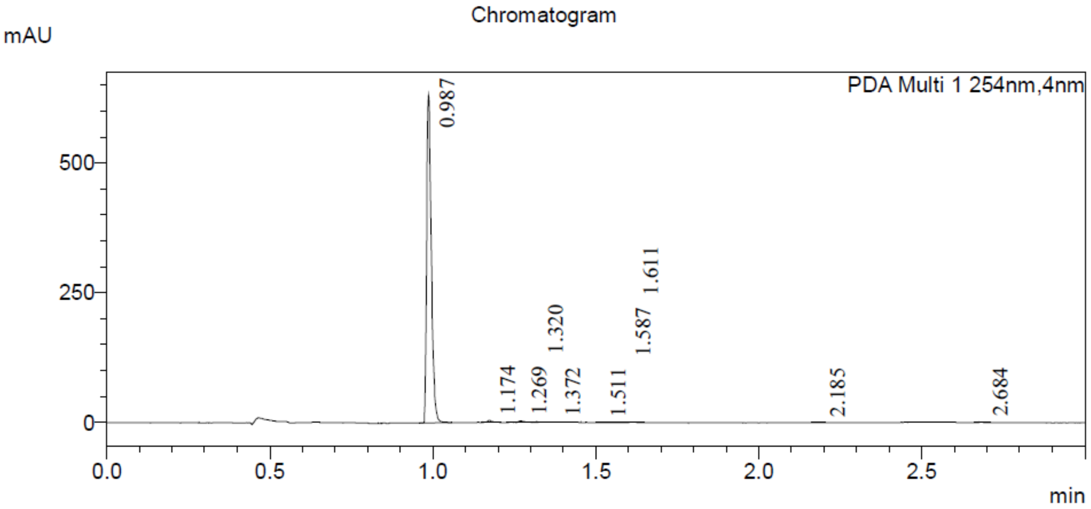

Peak Table

PDA Ch1 254nm

Peak#	Ret. Time	Height	Height%	Area	Area%
1	0.987	622402	98.318	622866	97.960
2	1.174	3678	0.581	3667	0.577
3	1.269	2447	0.387	3139	0.494
4	1.320	936	0.148	1148	0.180
5	1.372	563	0.089	1007	0.158
6	1.511	466	0.074	496	0.078
7	1.587	442	0.070	696	0.110
8	1.611	961	0.152	1308	0.206
9	2.185	597	0.094	606	0.095
10	2.684	561	0.089	907	0.143
Total		633051	100.000	635841	100.000

Supplementary Fig. 30. HPLC chromatogram of compound 2h

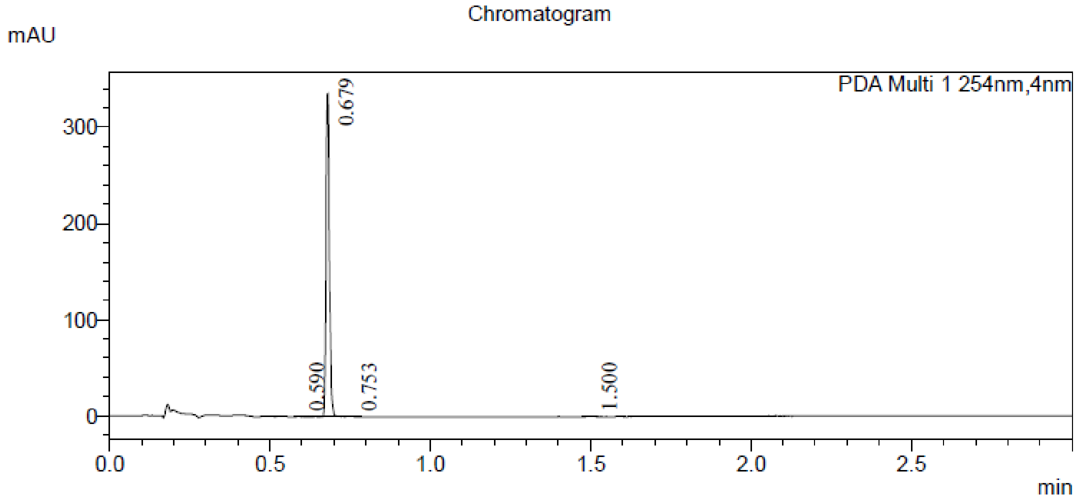

Peak Table

PDA Ch1 254nm

Peak#	Ret. Time	Height	Height%	Area	Area%
1	0.590	227	0.067	494	0.199
2	0.679	335292	99.642	246057	99.204
3	0.753	618	0.184	1021	0.412
4	1.500	359	0.107	460	0.186
Total		336495	100.000	248032	100.000

Supplementary Fig. 31. HPLC chromatogram of compound 3a

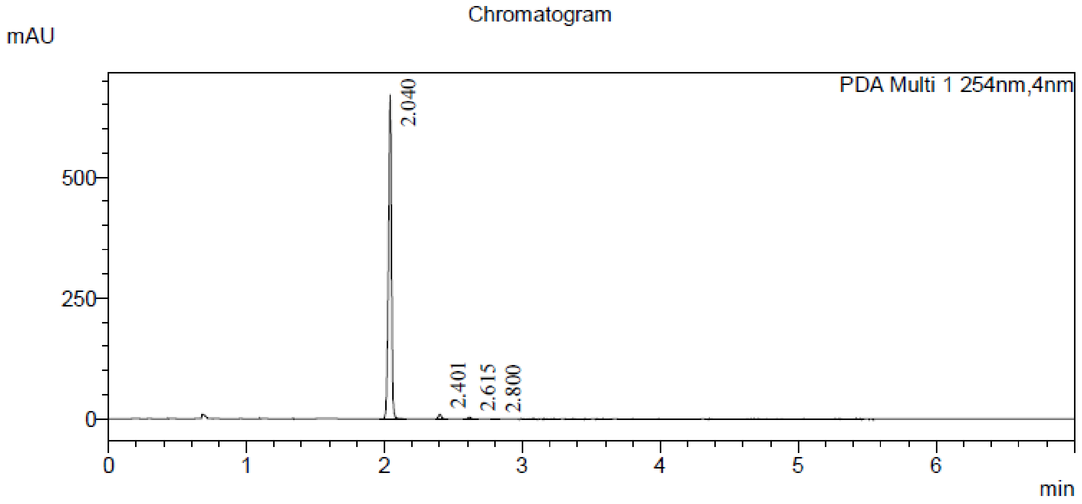

Peak Table

PDA Ch1 254nm

Peak#	Ret. Time	Height	Height%	Area	Area%
1	2.040	655894	98.116	1073877	98.192
2	2.401	7959	1.191	11963	1.094
3	2.615	3682	0.551	6141	0.562
4	2.800	952	0.142	1673	0.153
Total		668486	100.000	1093653	100.000

Supplementary Fig. 32. HPLC chromatogram of compound 3b

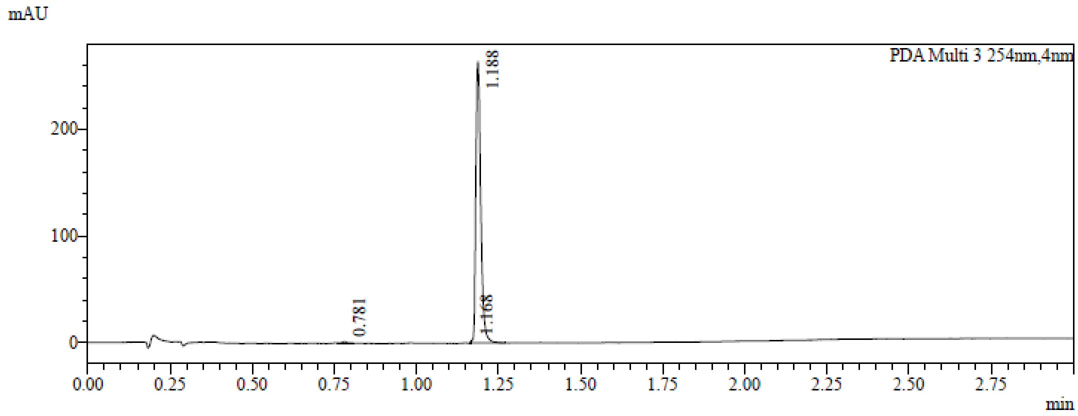

PDA Ch3 254nm

Peak#	Ret. Time	Height	Height%	Area	Area%
1	0.781	1206	0.490	1214	0.441
2	1.168	1369	0.557	663	0.241
3	1.188	243321	98.953	273463	99.318
Total				275339	100.000

Supplementary Fig. 33. HPLC chromatogram of compound 4

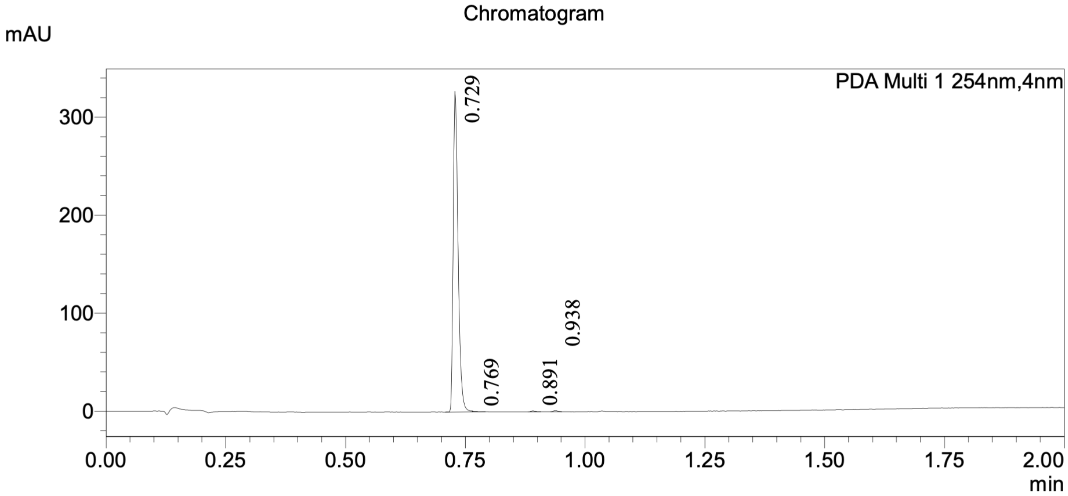

Peak Table

PDA Ch1 254nm

Peak#	Ret. Time	Height	Height%	Area	Area%
1	0.729	289158	99.147	232417	99.299
2	0.769	643	0.221	397	0.170
3	0.891	778	0.267	597	0.255
4	0.938	1066	0.366	648	0.277
Total		291645	100.000	234059	100.000

Supplementary Fig. 34. HPLC chromatogram of KMI169Ctrl

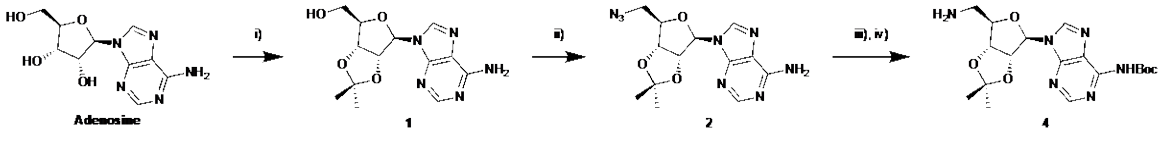

Supplementary Fig. 35. Synthetic route for compound 4. Reaction conditions: i) $CH(OEt)_3$, pTsOH, acetone, rt, 16 h, 92%; ii) DPPA, DBU, 1,4-dioxane, rt, 16 h then NaN_3, 15-crown-5, reflux, 6 h, 80%; iii) Boc_2O, NaH, THF, 0 °C to rt, 90 min, 80%; iv) H_2, Pd/C, EtOAc/MeOH, rt, 16 h, quant.

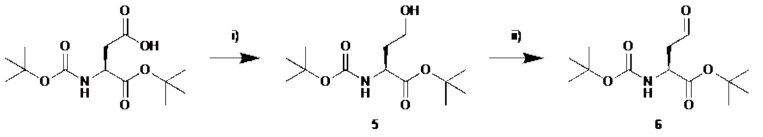

Supplementary Fig. 36. Synthetic route for compound 6. Reaction conditions: i) $ClCO_2CH_2CH(CH_3)_2$, Et_3N, THF, -5 °C, 30 min then $NaBH_4$, MeOH, 0 °C to rt, 2 h, 45%; ii) $(COCl)_2$, Et_3N, DMSO, CH_2Cl_2, -78 °C to rt, 60 min, 78%.

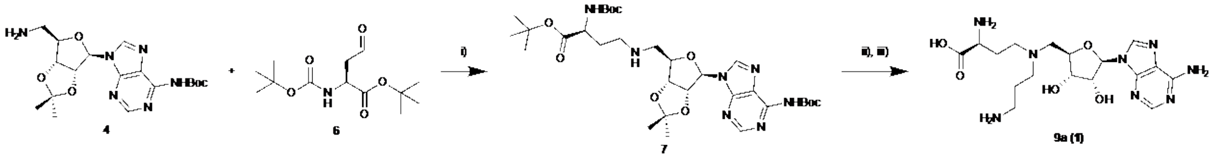

Supplementary Fig. 37. Synthetic route for compound 9a (1). Reaction conditions: i) AcOH, STAB, DCE, rt, 4 h, 43%; ii) tert-Butyl (3-oxopropyl)carbamate, STAB, AcOH, DCE, rt, 4 h, 40%; iii) TFA, water, rt, 6 h, 100%.

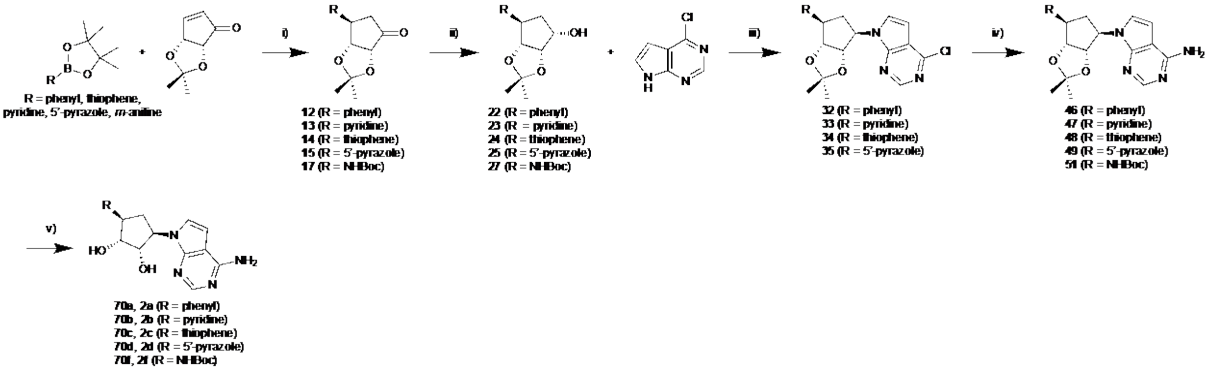

Supplementary Fig. 38. Synthetic routes to compounds 2a, 2b, 2c, 2d, and 2f. Reaction conditions: i) $[Rh(COD)Cl]_2$, KOH, 1,4-dioxane, 80 °C, 3-7 h; ii) $NaBH_4$, MeOH, 0 °C to rt, 1 h; iii) Tf_2O, pyridine, DCM, 0 °C, 1 h, then NaH, DMF, 0 °C to rt, 17 h (**32**), DIAD, PPh**3**, THF, 0 °C to rt, 17 h (**33**, **34**, **35**); iv) NH_3, 1,4-dioxane, 100 °C, 17 h (**46**, **47**, **48**, **49**), DBAD, PPh_3, PhMe, 0 °C to 80 °C, 17 h then NH_3, 1,4-dioxane, 100 °C, 17 h (**51**); v) TFA, water, rt, 3 h.

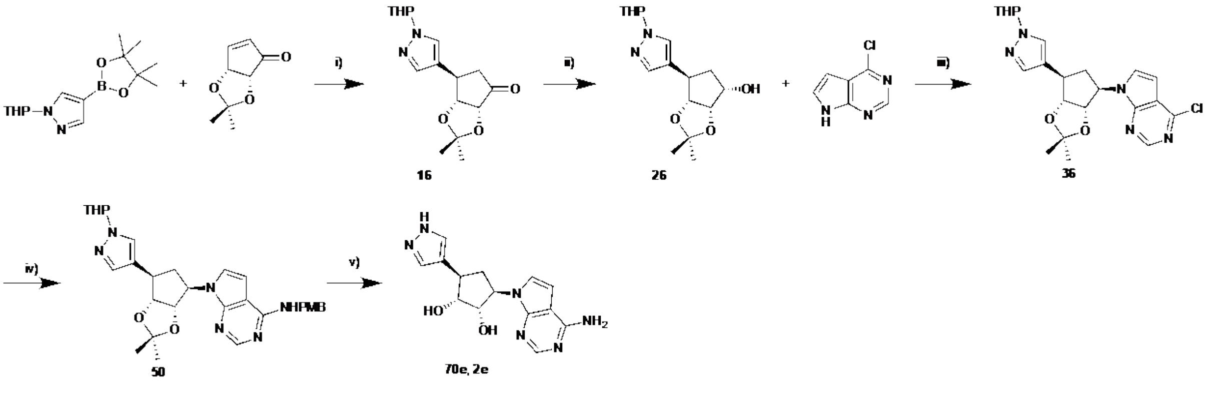

Supplementary Fig. 39. Synthetic route for compound 2e. Reaction conditions: i) $Rh(acac)(C_2H_4)_2$, (R)-BINAP, 1,4-dioxane, water, 100 °C, 2h; ii) $NaBH_4$, MeOH, 0 °C, 30 min; iii) PPh_3, DIAD, THF, 0 °C to rt, 12 h; iv) $PMBNH_2$, TEA, EtOH, 80 °C, 1 h; v) TFA, 80 °C, 30 min.

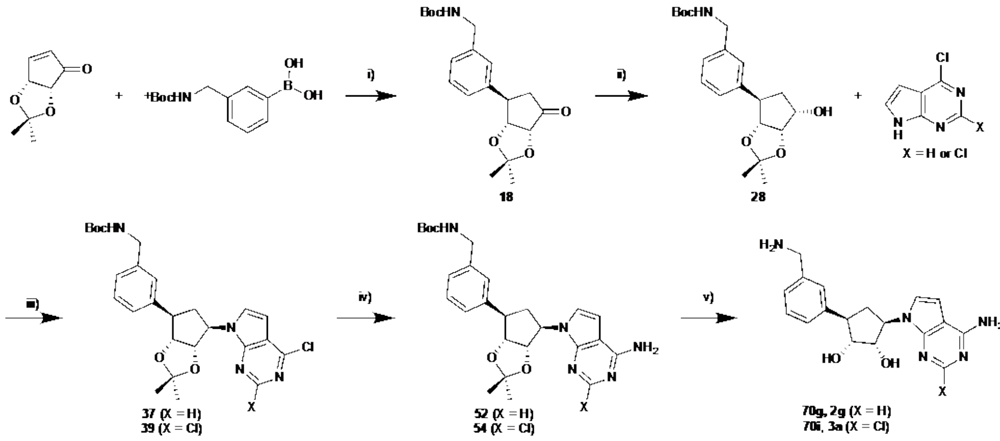

Supplementary Fig. 40. Synthetic routes for compounds 2g and 3a. Reaction conditions: i) Rh(acac)(C_2H_4)$_2$, (*R*)-BINAP, 1,4-dioxane, water, 100 °C, 2h; ii) $NaBH_4$, MeOH, 0 °C, 30 min; iii) PPh_3, DBAD, PhMe, 0 °C to 50 °C, 2 h (**37**), PPh_3, DIAD, THF, 0 °C to 40 °C, 10 h (**39**); iv) NH_3, MeOH, 80 °C, 8 h; v) TFA, DCM, 80 °C, 1 h (**70g**), HCl, MeOH, 50 °C, 20 min (**70i**).

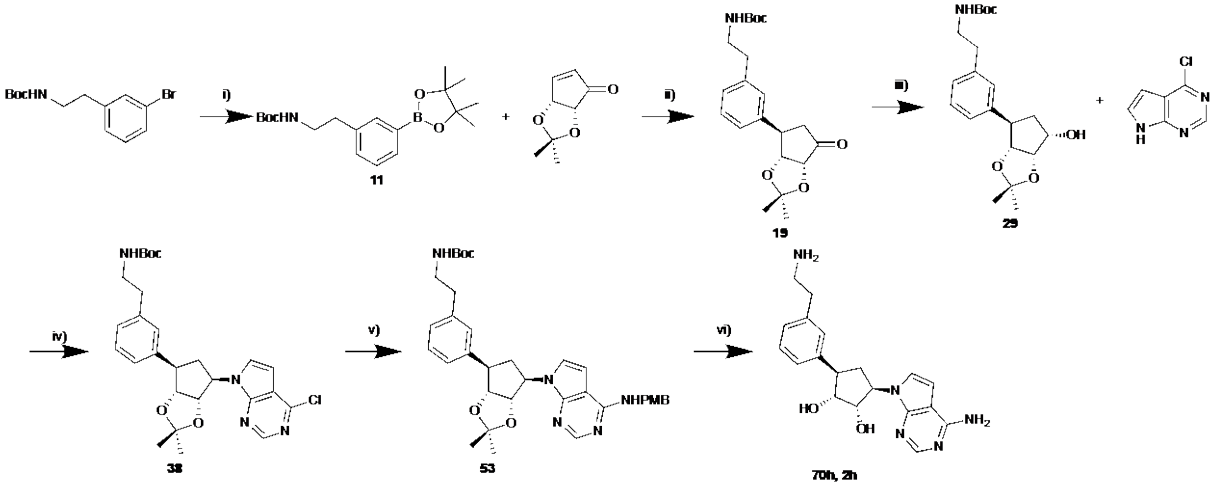

Supplementary Fig. 41. Synthetic route for compound 2h. Reaction conditions: i) B_2Pin_2, Pd(dppf)Cl_2, KOAc, 1,4-dioxane, 100 °C, 2 h, 52%; ii) Rh(acac)(C_2H_4)$_2$, (*R*)-BINAP, 1,4-dioxane, water, 100 °C, 3 h, 84%; iii) $NaBH_4$, MeOH, 0 °C, 30 min, 56%; iv) PPh_3, DIAD, THF, 0 °C to rt, 12 h, 88%; v) $PMBNH_2$, TEA, EtOH, 80 °C, 2 h, 44%; vi) TFA, DCM, 80 °C, 30 min, 18%.

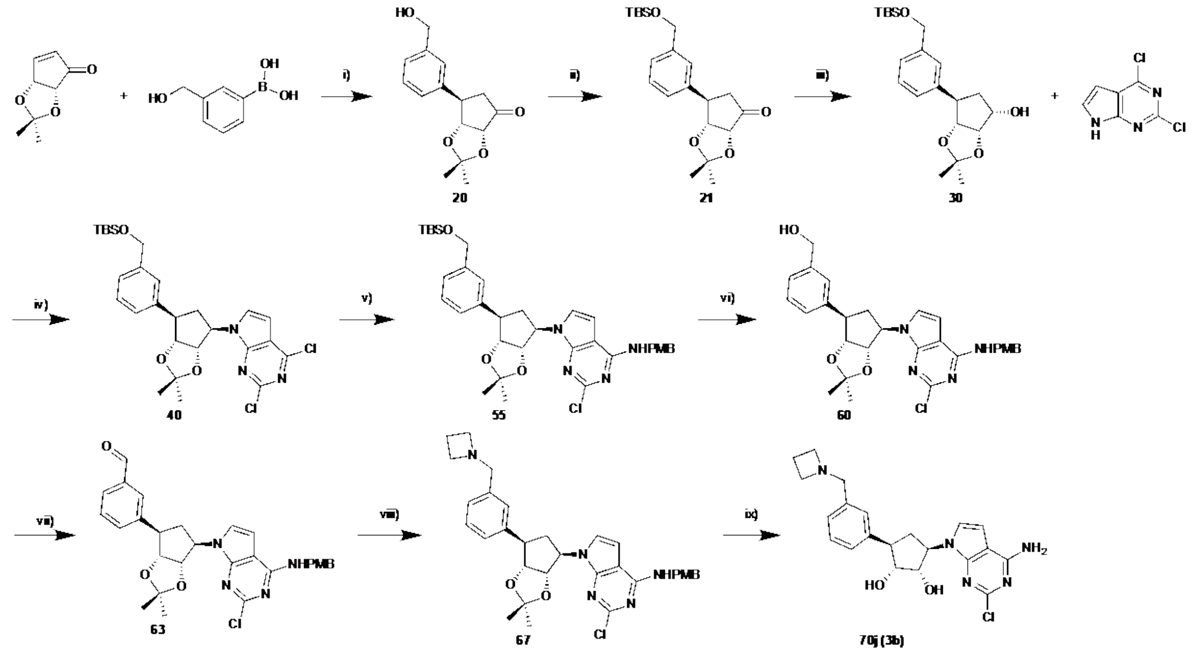

Supplementary Fig. 42. Synthetic route for compound 3b. Reaction conditions: i) Rh(acac)(C_2H_4)$_2$, (*R*)-BINAP, 1,4-dioxane, water, 100 °C, 2 h, 74%; ii) TBSCl, imidazole, DMF, 0 °C to rt, 2 h, 82%; iii) $NaBH_4$, MeOH, 0 °C, 30 min, 77%; iv) PPh_3, DBAD, PhMe, 0 °C to 50 °C, 3 h, 43%; v) $PMBNH_2$, TEA, EtOH, 80 °C, 2 h, 65%; vi) TBAF, THF, rt, 1 h, 91%; vii) MnO_2, DCM, rt, on, 91%; viii) azetidine, STAB, AcOH, DCM, rt, 30 min, 92%; ix) TFA, 60 °C, 30 min, 29%.

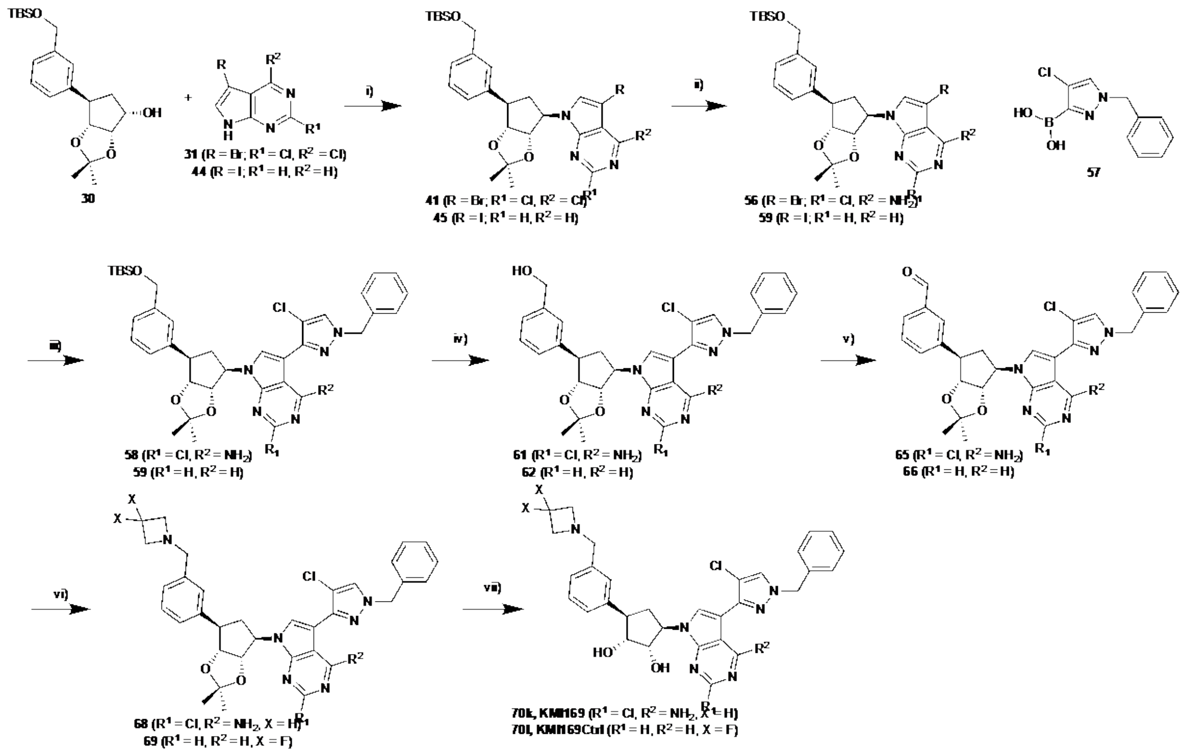

Supplementary Fig. 43. Synthetic routes for KMI169 and KMI169Ctrl. Reaction conditions: i) PPh_3, DBAD, PhMe, 0 °C to 50 °C, 3 h (**41**), PPh_3, DIAD, THF, rt, 6 h; ii) NH_3, MeOH, 80 °C, 2 h; iii) Pd(dtbpf)Cl_2, K_3PO_4, 1,4-dioxane, water, 80 °C, 1 h (**58**), Pd(dppf)Cl_2, K_3PO_4, 1,4-dioxane, water, 60 °C, 2 h (**59**); iv) TBAF, THF, rt, 30 min; v) MnO_2, DCM, 40 °C, 24 h (**65**), MnO_2, DCM, 40 °C, 4 h (**66**), vi) azetidine, $NaBH_3CN$, AcOH, DCM, rt, 30 min (**68**), 3,3-difluroazetidine, $NaBH_3CN$, DIPEA, DCM, rt, 30 min (**69**); vii) TFA, MeOH, 50 °C, 30 min (**70k**), TFA, 40 °C, 30 min (**70l**).

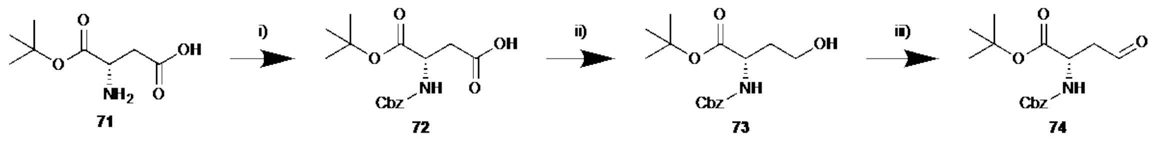

Supplementary Fig. 44. Synthetic route for compound 74. Reaction conditions: i) CbzCl, $NaHCO_3$, 1,4-dioxane/water, rt, 2 h, 23%; ii) BH3 DMS, THF, rt, 5 h, 48%; iii) PCC, DCM, rt, 4 h, 75%.

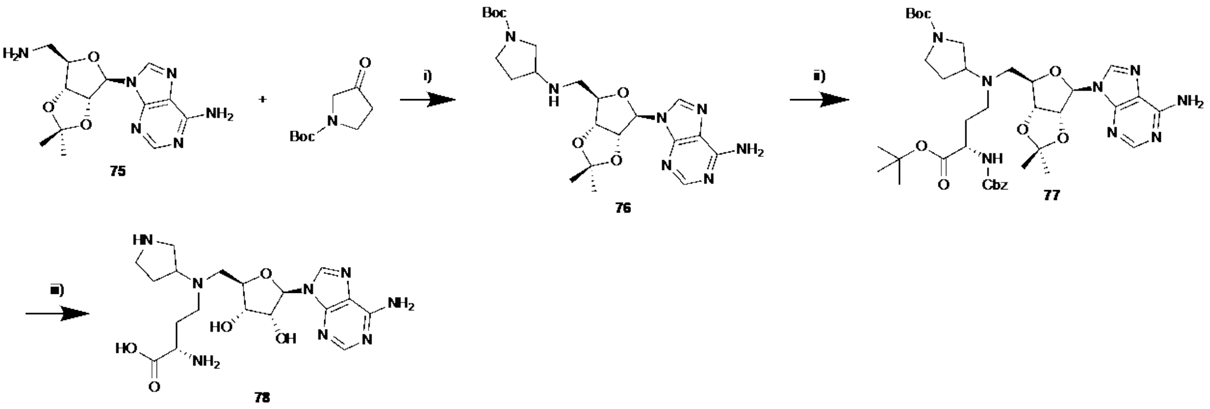

Supplementary Fig. 45. Synthetic route for compound 78. Reaction conditions: i) $NaBH_3CN$, AcOH, MeOH, rt, 2 d, 26%; ii) **74**, $NaBH_3CN$, AcOH, MeOH, 35 °C, 2 d, 58%; iii) HBr, AcOH, rt , 4 h, 18%.

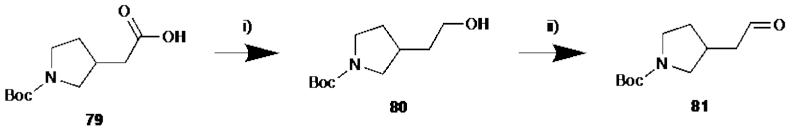

Supplementary Fig. 46. Synthetic route for compound 81. Reaction conditions: i) BH_3 DMS, THF, 50 °C, 17 h, 65%; iii) PCC, DCM, rt, 4 h, 100%.

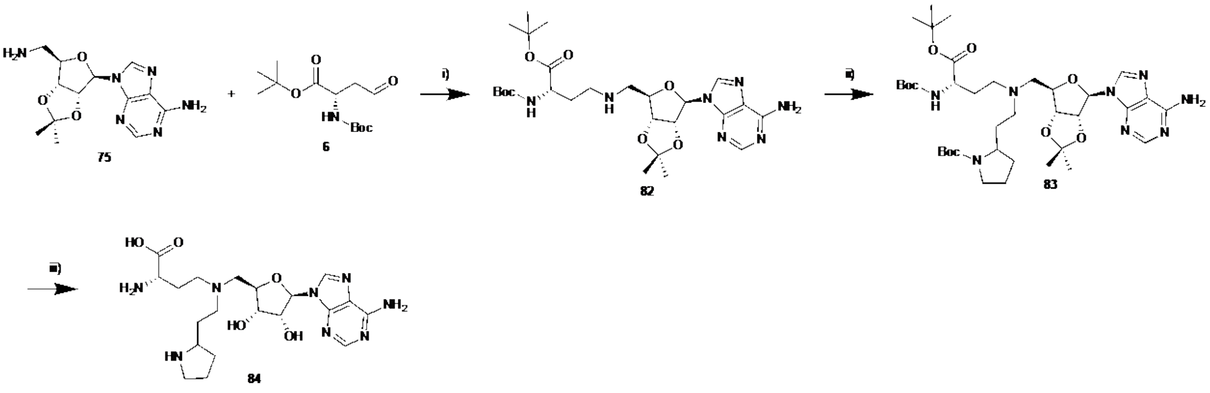

Supplementary Fig. 47. Synthetic route for compound 84. Reaction conditions: i) $NaBH_3CN$, AcOH, MeOH, 65 °C, 17 h, 36%; ii) **81**, $NaBH_3CN$, AcOH, MeOH, 65 °C, 17 h, 50%; iii) HBr, AcOH, rt , 4 h, 28%.

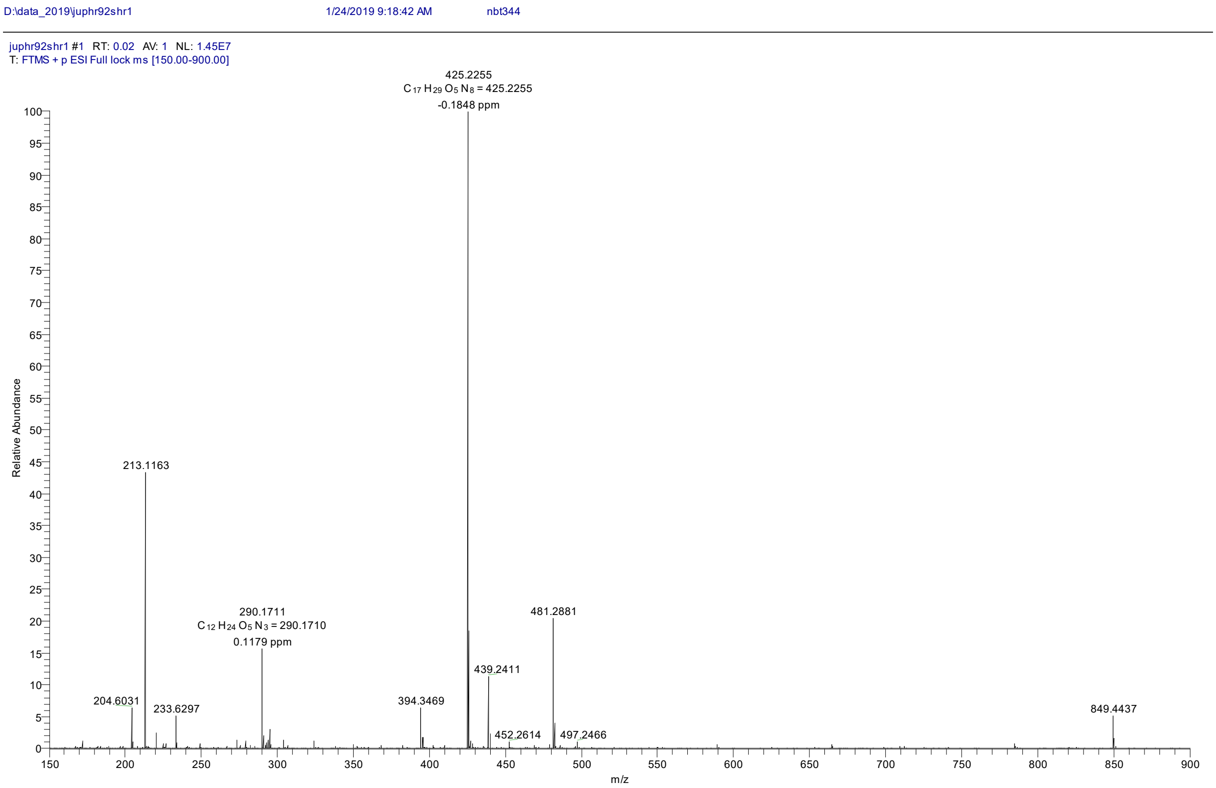

Supplementary Fig. 48. Mass spectrometry of compound 1a.

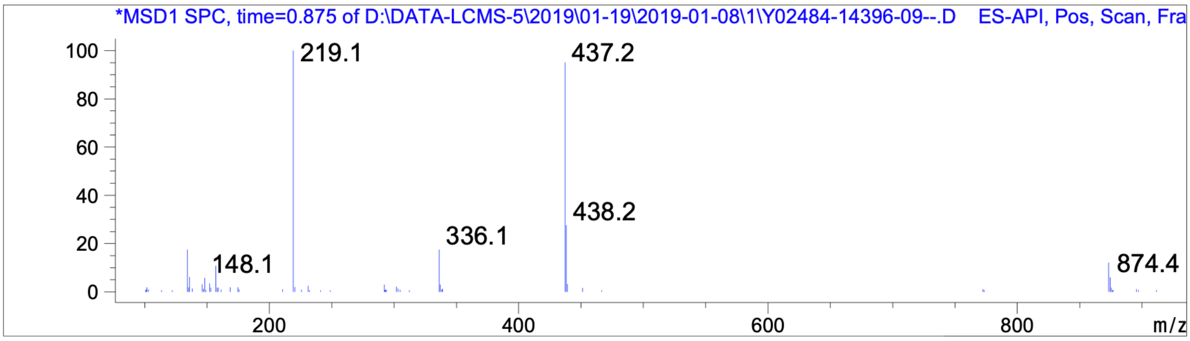

Supplementary Fig. 49. Mass spectrometry of compound 2b.

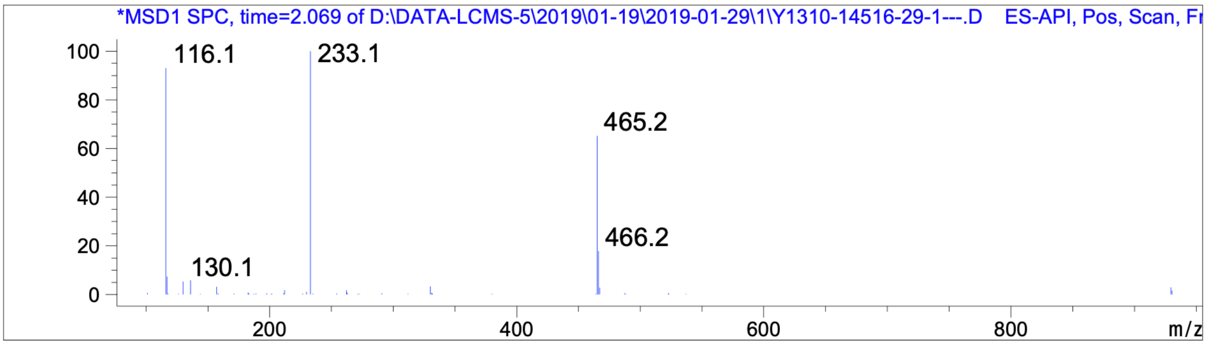

Supplementary Fig. 50. Mass spectrometry of compound 1c.

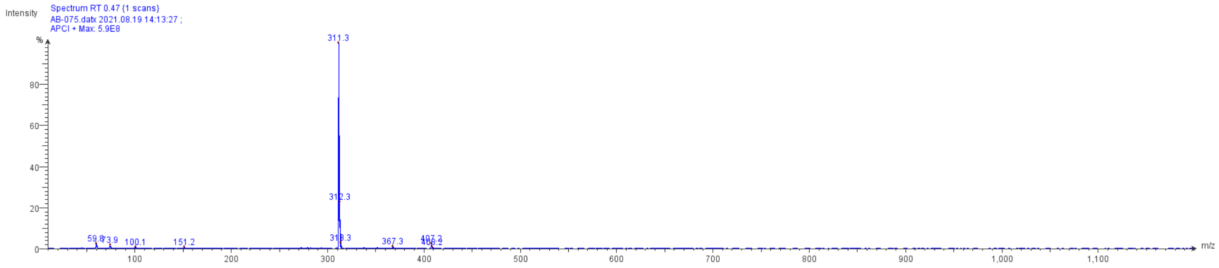

Supplementary Fig. 51. Mass spectrometry of compound 2a.

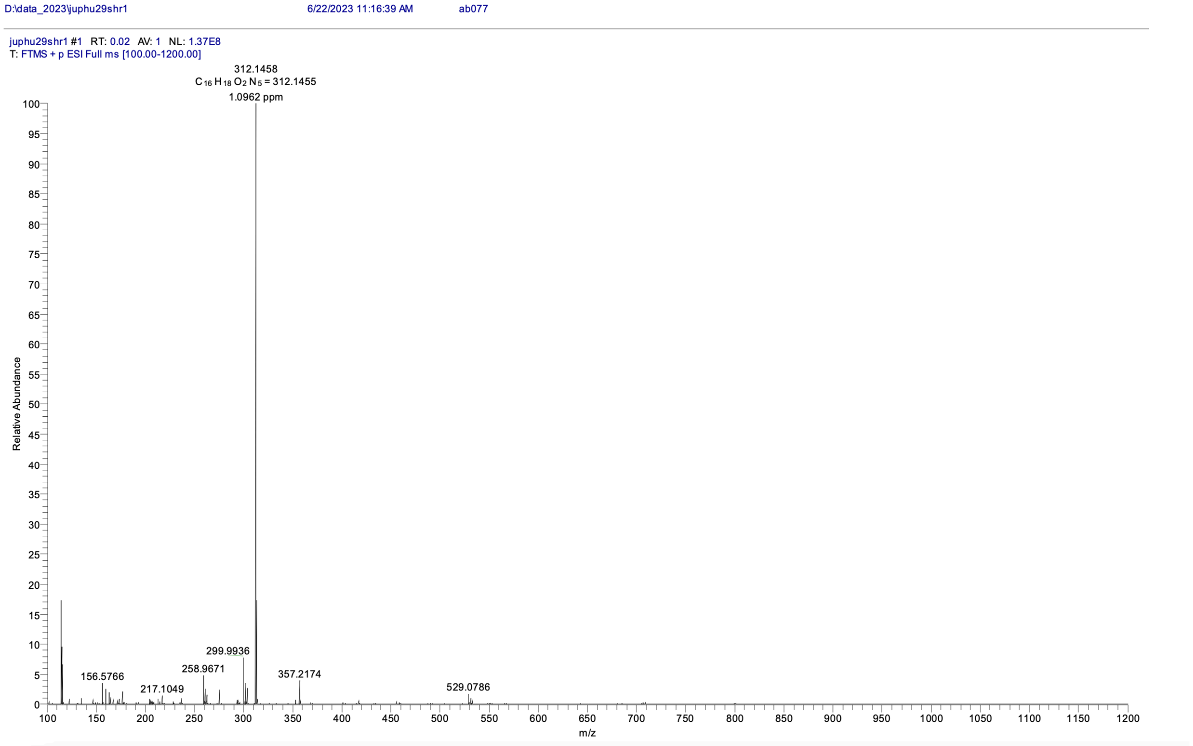

Supplementary Fig. 52. Mass spectrometry of compound 2b.

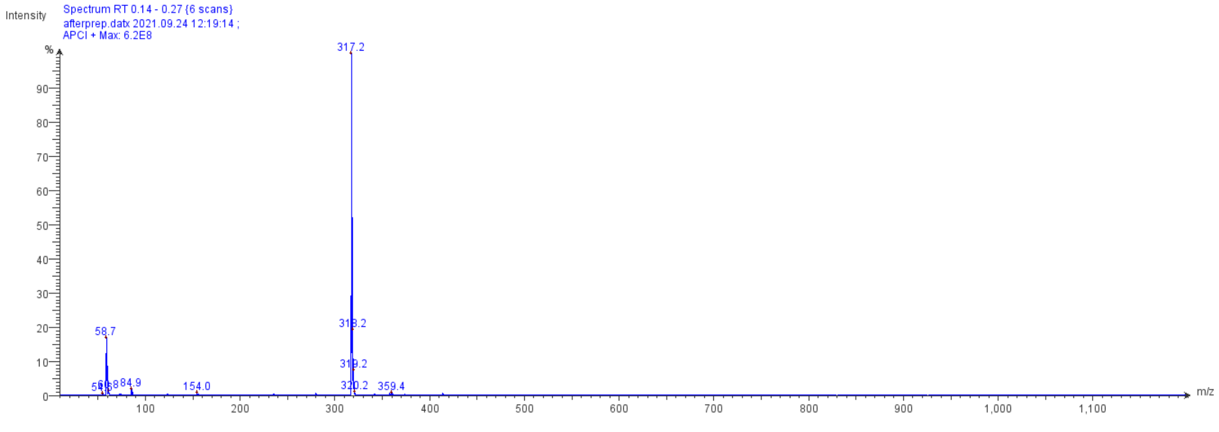

Supplementary Fig. 53. Mass spectrometry of compound 2c.

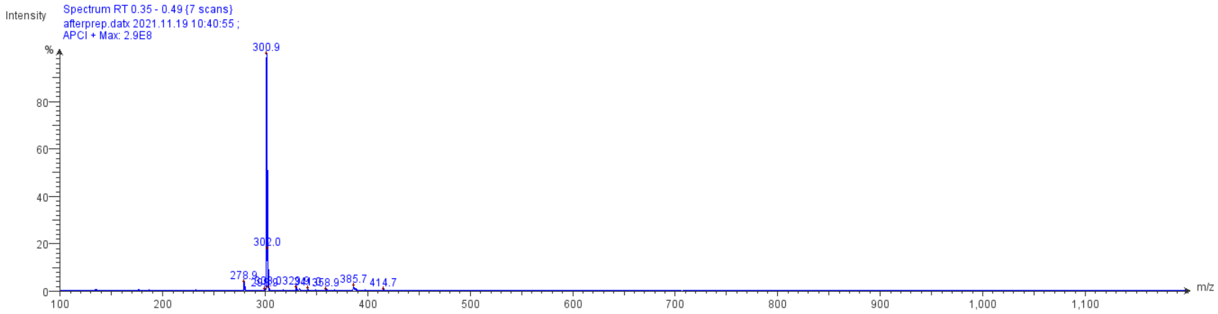

Supplementary Fig. 54. Mass spectrometry of compound 2d.

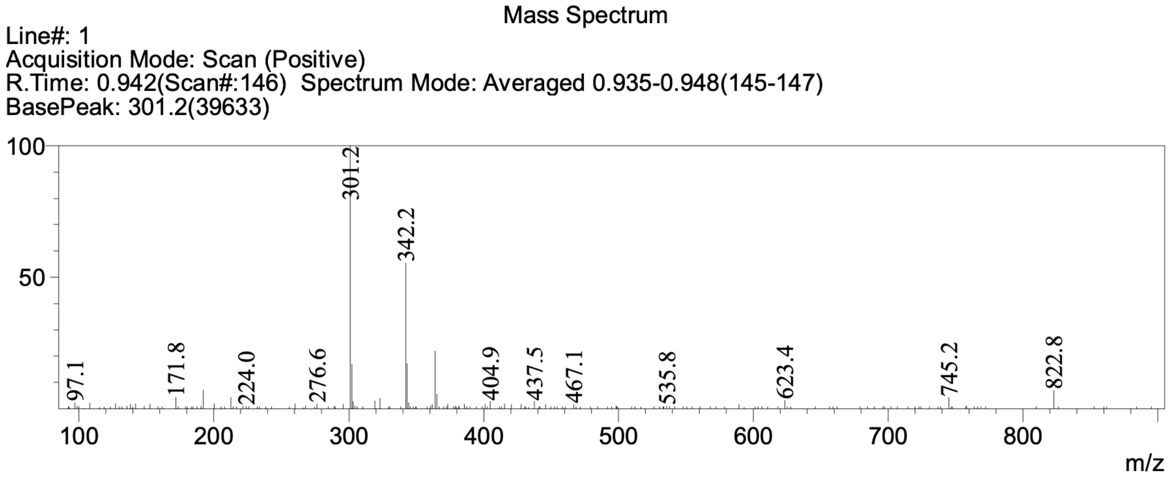

Supplementary Fig. 55. Mass spectrometry of compound 2e.

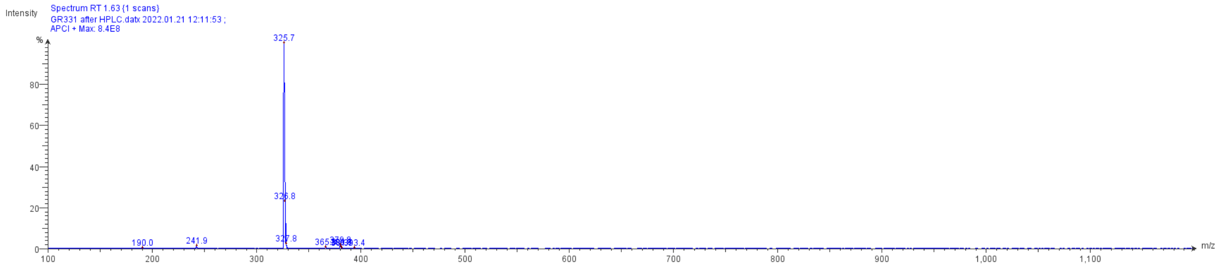

Supplementary Fig. 56. Mass spectrometry of compound 2f.

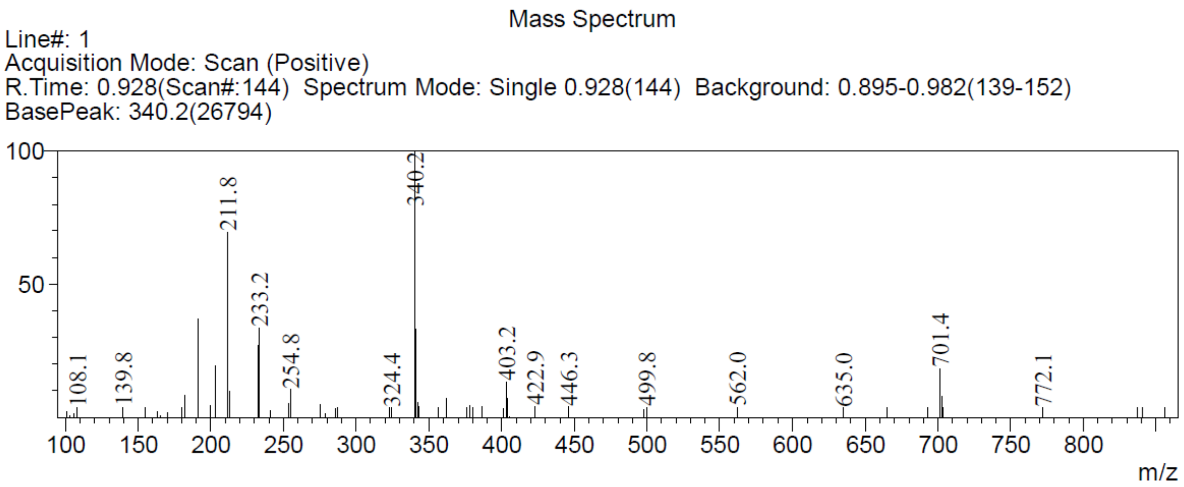

Supplementary Fig. 57. Mass spectrometry of compound 2g.

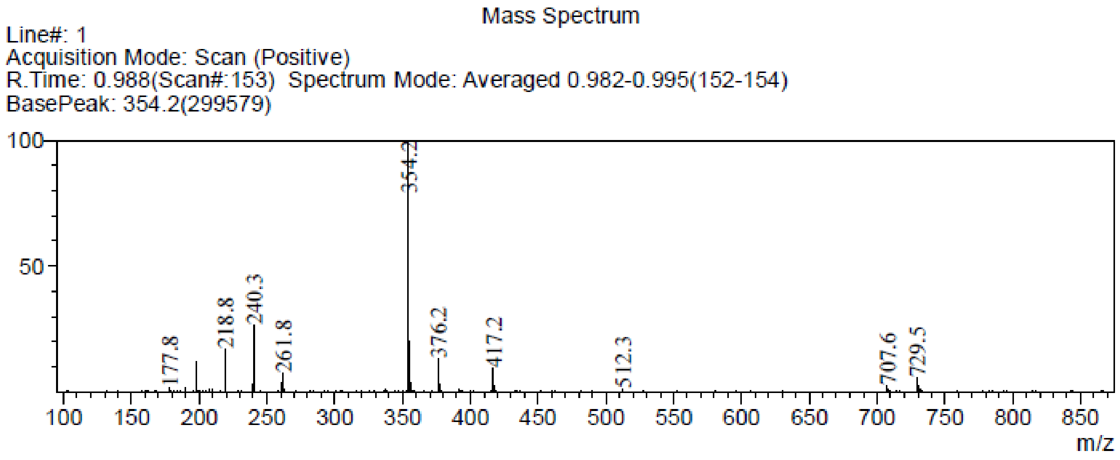

Supplementary Fig. 58. Mass spectrometry of compound 2h.

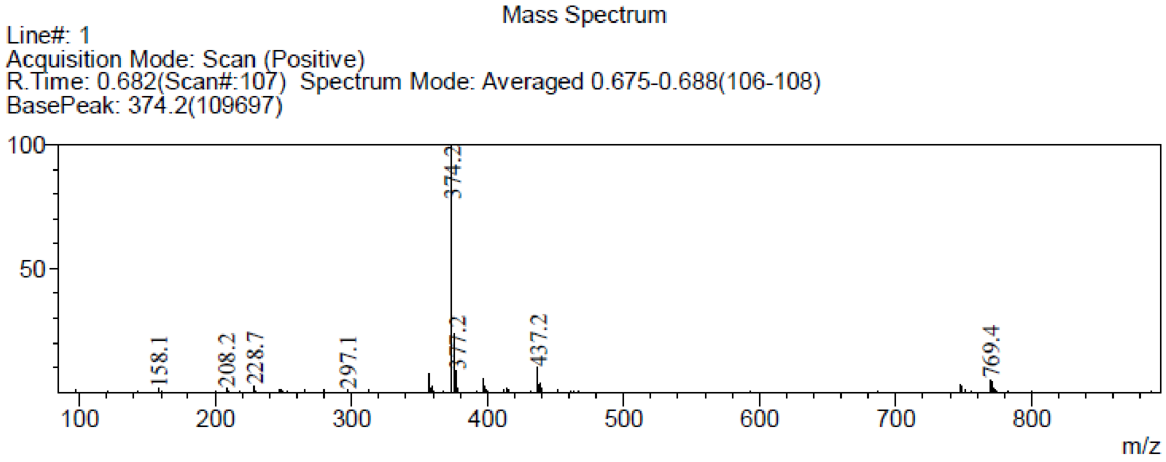

Supplementary Fig. 59. Mass spectrometry of compound 3a.

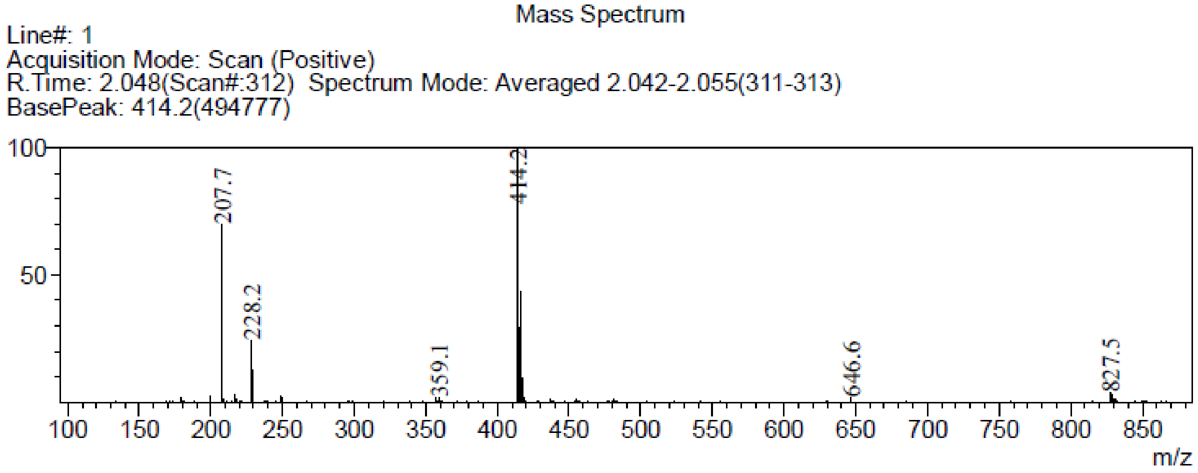

Supplementary Fig. 60. Mass spectrometry of compound 3b.

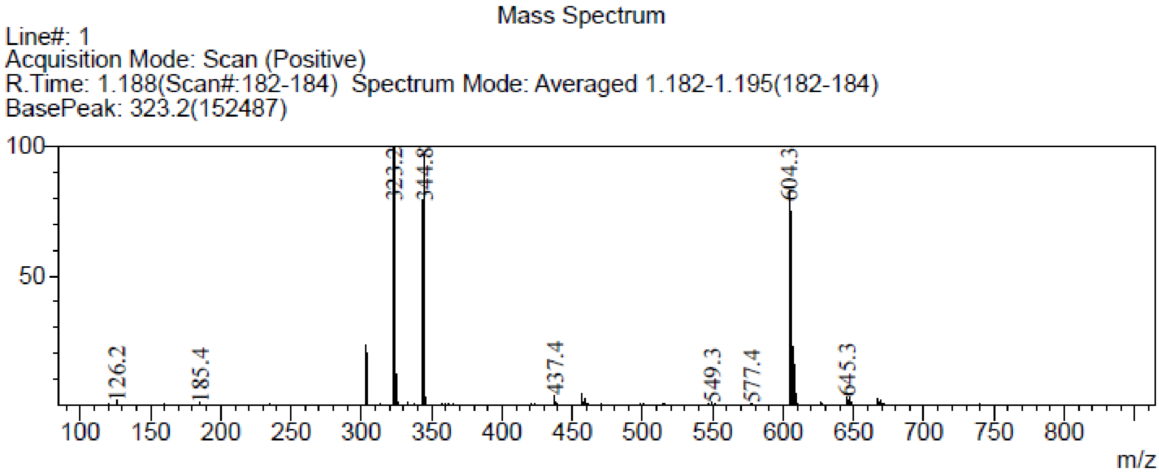

Supplementary Fig. 61. Mass spectrometry of compound 4/KMI169.

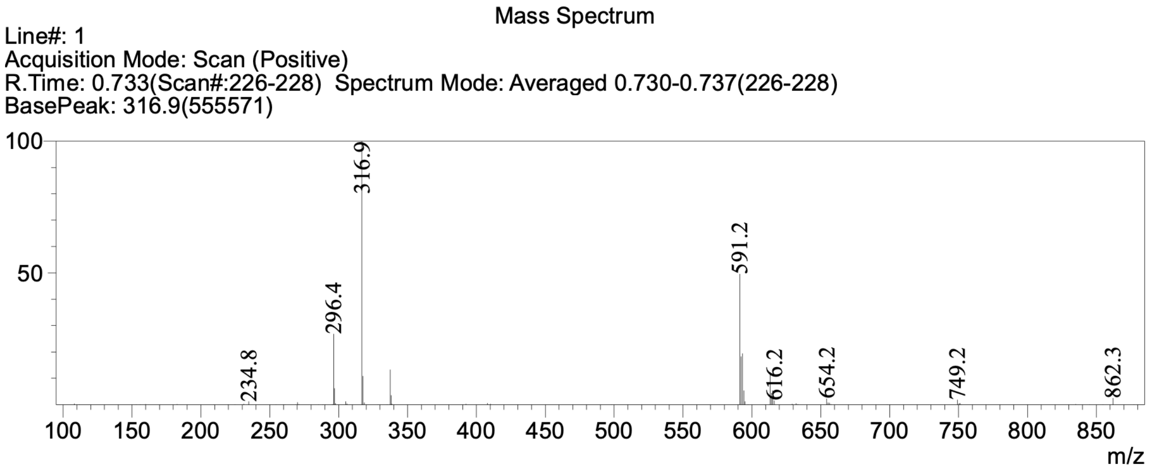

Supplementary Fig. 62. Mass spectrometry of KMI169Ctrl.

Supplementary Tables

Supplementary Table 1. Data collection and Refinement Statistics

	KMT9+compound 1a	KMT9+compound 1b	KMT9+compound 1c
**Data collection**			
Space group	$P6_122$	$P6_122$	$P6_122$
Cell dimensions			
a, *b*, *c* (Å)	110.3, 110.3, 129.9	109.8, 109.8, 130.7	110.5, 110.5, 129.8
α, β, γ (°)	90.0, 90.0, 120.0	90.0, 90.0, 120.0	90.0, 90.0, 120.0
Resolution (Å)	47.78 - 1.46 (1.49 - 1.46) *	47.57 - 1.39 (1.44 - 1.39) *	47.85 – 1.47 (1.49 – 1.47) *
Mean I/sigma (I)	23.8 (1.6)	19.8 (1.0)	26.4 (0.9)
R_{merge}	0.130 (2.798)	0.153 (4.921)	0.095 (4.189)
$CC_{1/2}$	1.000 (0.579)	1.000 (0.412)	1.000 (0.448)
Completeness (%)	99.9 (98.4)	99.9 (98.7)	99.8 (96.5)
Redundancy	38.9 (34.5)	39.0 (36.7)	39.4 (29.6)
**Refinement**			
Resolution (Å)	47.78 - 1.46 (1.49 - 1.46) *	47.57 - 1.39 (1.44 - 1.39) *	47.85 – 1.47 (1.52 – 1.47)
No. reflections	80784	93095	79631
R_{work} / R_{free}	0.143/0.180	0.152/0.179	0.158/0.190
No. atoms			
Protein	2465	2504	2474
Ligand	30	31	33
Solvent	172	167	184
Average *B*-factor			
Overall	22.00	24.92	27.60
Protein	22.33	24.45	27.02
Solvent	30.57	33.57	36.96
Ligand	14.37	16.79	18.65
R.m.s. deviations			
Bond lengths (Å)	0.018	0.013	0.017
Bond angles (º)	2.06	1.82	1.78
**Ramachandran Plot**			
Favored	98.38	97.74	98.06
Allowed	1.30	2.26	1.94
Not Allowed	0.32	0.00	0.00

*Single crystal was used the dataset; *Values in parentheses are highest-resolution shell

Supplementary Table 2. Abbreviations list for Synthesis

**Abbreviation**	**Name**
**Et_3N**	Triethylamine
**rt**	Room temperature
**MeOH**	Methanol
**CH_2Cl_2 or DCM**	Dichloromethane
**AcOH**	Acetic acid
**PPh_3**	Triphenylphosphine
**PhMe**	toluene
**EtOAc or AcOEt**	Ethyl acetate
**MeCN**	Acetonitrile
**DMF**	*N*,*N*-Dimethylformamide
**$CH(OEt)_3$**	Triethyl orthoformate
**pTsOH**	*p*-Toluenesulfonic acid
**DPPA**	Diphenylphosphoryl azide
**DBU**	1,8-Diazabicyclo[5.4.0]undec-7-ene
**NaN_3**	Sodium azide
**Boc**	*tert*-butoxycarbonyl
**TFA**	Trifluoroacetic acid
**Boc_2O**	Di-*tert*-butyl dicarbonate
**NaH**	Sodium hydride
**THF**	Tetrahydrofuran
**DBAD**	Di-*tert*-butyl azodicarboxylate
**$NaBH_4$**	Sodium borohydride
**$NaBH(OAc)_3$**	Sodium triacetoxyborohydride
**Tf_2O**	Trifluoromethanesulfonic anhydride
**$NaHCO_3$**	Sodium bicarbonate
**KOH**	Potassium hydroxide
**KAc**	Potassium acetate
**DMSO**	Dimethyl sulfoxide
**DCE**	1,2-Dichloroethane
**$[RhCl(COD)]_2$**	Cyclooctadiene rhodium chloride dimer
**B_2Pin_2**	Bis(pinacolato)diboron
**H_2**	Hydrogen
**Pd/C**	Palladium over charcoal
**BINAP**	2,2′-bis(diphenylphosphino)-1,1′-binaphthyl

Supplementary Methods

All reactions were carried out in glassware under inert (nitrogen) atmosphere. All used chemicals and reagents were purchased from diverse suppliers and were used without further purification. Solvents were freshly purified by distillation/drying over molecular sieves following the instructions from the Purification Book. Particularly mentioned anhydrous/dry solvents were purchased from Acros organics. Reactions were monitored by thin-layer chromatography (TLC) performed with Merck alumina plates coated with silica gel 60 F254, silica gel 60 RP-18 F254s or silica gel 60 NH_2 $F_{254}S$ (layer thickness: 0.2 mm) and analyzed under UV light (254 nm and 365 nm) or revealed using $KMnO_4$, Bromocresol green, ninhydrin, phosphomolybdic acid or 2,4-dinitrophenylhydrazine (2,4-DNPH) as staining agent. The composition of the mobile phase was adjusted to the compound properties. Yields were not particularly optimized. Flash column chromatography was performed on a Biotage® Isolera Prime/One or on a Biotage® Selekt Enkel purification system using 40–60 µm pre-packed silica gel columns from Biotage®, HP-spherical 50 µm pre-packed silica gel columns from Interchim (Jumbo Pack), Sfär Silica D 60 µm, Sfär KP amino D 50 µm or Sfär Silica HC D 20 µm pre-packed silica gel columns from Biotage®. NMR spectroscopy and mass spectrometry were used for product identification. Bruker Avance 400 spectrometer operating at 400 MHz, 300 MHz, for 1H, 19F, and 13C acquisitions, respectively with TopSpin. Chemical shifts are reported in ppm with the solvent residual peak as the internal standard. For ^{1}H NMR signals were referenced to: d6-DMSO, 2.50; CD_3OD, 3.31; $CDCl_3$, 7.26; For ^{13}C NMR to: d6-DMSO, 39.5; CD_3OD, 49.0; $CDCl_3$, 77.16, multiplicity abbreviations are as follows: bs = broad singlet, s = singlet, d = doublet, dd = doublet of doublets, dt = doublet of triplets, t = triplet, td = triplet of doublets, q = quartet, m = multiplet, coupling constant (*J*) are expressed in Hz. The ^{1}H assignment resulted from COSY experiments. Spectral analysis were done with MestreNova 14. Mass spectra were recorded on an Advion expression CMS using an ASAP® (Atmospheric Solids Analysis Probe; aka APCI: Atmospheric

Pressure Chemical Ionization) as ion source, on a Thermo Scientific Exactive mass spectrometer using electrospray ionization (ESI) as ion source or HR-MS were obtained on a THERMO SCIENTIFIC Advantage. Preparative HPLC was performed for all final compounds on an Agilent 1260 Infinity II using either method **D** or **E** at 210 nm. HPLC analysis was performed to determine the purity of all final compounds on an Agilent Technologies 1260 Infinity II system using diode array detector (DAD) UV detection at either 210, 230, 248, 254, 260 & 280 nm. LCMS was performed on a Shimadzu LCMS2020 in with an ESI interface using the Scan mode in positive polarity. m/z Range was 90-900, Scan speed 2143/u/sec.

Method A: XBridge® Shield RP18 5µm XB-C18 100 Å 150 x 4.6 mm column and eluent A was H_2O containing 0.05 % trifluoracetic acid (TFA) and eluent B was CH_3CN containing 0.05 % TFA. Linear gradient conditions were as follows: 0–9 min: 100:0 (A/B); 9–11 min: 0:100 (A/B); 11–13 min: 0:100; (A/B); 13–14 min: 100:0 (A/B); 14–16 min: 100:0 (A/B) with a flowrate of 0.95 $mL.min^{-1}$.

Method B: Phenomenex Kinetex® 5µm XB-C18 100 Å 250 x 4.6 mm column and eluent A was H_2O containing 0.05 % trifluoracetic acid (TFA) and eluent B was CH_3CN containing 0.05 % TFA. Linear gradient conditions were as follows: 0–4 min: 90:10 (A/B); 4–29 min: 90:0→100 (A/B); 29–31 min: 0:100; (A/B); 31–31.5 min: 90:10 (A/B); 31.5–40 min: 90:10 (A/B) with a flowrate of 1.00 $mL.min^{-1}$.

Method C: Phenomenex Kinetex® 5µm XB-C18 100 Å 250 x 4.6 mm column and eluent A was H_2O containing 0.05 % trifluoracetic acid (TFA) and eluent B was CH_3CN containing 0.05 % TFA. Linear gradient conditions were as follows: 0–1 min: 100:0 (A/B); 1–9 min: 60:40 (A/B); 9–11 min: 5:95; (A/B); 11–13 min: 5:95 (A/B); 13–14 min: 100:0 (A/B); 14–16 min: 100:0 (A/B) with a flowrate of 0.95 $mL.min^{-1}$.

Method D: XBridge® Shield RP18 5µm XB-C18 100 Å 150 x 4.6 mm column and eluent A was H_2O containing 0.05 % trifluoracetic acid (TFA) and eluent B was CH_3CN

containing 0.05 % TFA. Linear gradient conditions were as follows: 0–1 min: 100:0 (A/B); 1–9 min: 60:40 (A/B); 9–11 min: 5:95; (A/B); 11–13 min: 5:95 (A/B); 13–14 min: 100:0 (A/B); 14–16 min: 100:0 (A/B) with a flowrate of 0.95 mL.min^{-1}.

Purification methods:

Method E: Phenomenex Kinetex® 5μM XB-C18 100 Å 250 x 21.2 mm column and eluent A was H_2O containing 0.05 % trifluoracetic acid (TFA) and eluent B was CH_3CN containing 0.05 % TFA. Linear gradient conditions were as follows: 0–1 min: 100:0 (A/B); 1–9 min: 100:0→60:40 (A/B); 9–11 min: 60:40→5:95; (A/B); 11–13 min: 5:95 (A/B); 13–14 min: 5:95→100:0 (A/B); 14–20 min: 100:0 (A/B) with a flowrate of 20.20 mL.min^{-1}.

Method F: Phenomenex Kinetex® 5 μM XB-C18 100 Å 250 x 21.2 mm column and eluent A was H_2O containing 0.05 % trifluoracetic acid (TFA) and eluent B was CH_3CN containing 0.05 % TFA. Linear gradient conditions were as follows: 0–4 min: 90:10 (A/B); 4–29 min: 90:0→100 (A/B); 29–31 min: 0:100; (A/B); 31–31.5 min: 90:10 (A/B); 31.5–40 min: 90:10 (A/B) with a flowrate of 22.00 mL.min^{-1}.

2nd reductive amination (8a) and the final deprotection general procedure A (9a)

To a stirred solution of 7 (1.1 eq.) and corresponding aldehydes (1 eq.) in dry DCE (0.12 m based on 7) was added AcOH (1.1 eq.). The solution was stirred for 4 h at rt, then $NaBH(OAc)_3$ (2.6 eq.) was added and the mixture was stirred for 4 h at rt and 12 h at 70 ° C. After completion, the reaction was quenched by the addition of a 5 % aq. $NaHCO_3$ solution and the phases were separated. The aqueous phase was then extracted 3 times with CH_2Cl_2 and the combined organic phases once with brine. Drying over Na_2SO_4, filtration and evaporation afforded the crude product that was subjected to silica gel column chromatography eluting with CH_2Cl_2/MeOH (mostly 99.5:0.5 - 94:6) to afford the tertiary amines as yellow oils.

Tertiary amines 8a was dissolved (0.02 m) in freshly prepared TFA/H_2O (4:1) solution and stirred at rt for 6 h, then evaporated to give the desired product 9a as foam (3 TFA salts).

Rh-catalyzed 1,4-addition general procedure B

To a heat-dried three-necked round bottom flask equipped with a stirring bar and air condenser was charged with (3a*R*,6a*R*)-2,2-dimethyl-3a,6a-dihydro-4*H*-cyclopenta[*d*][1,3]dioxol-4-one (1.00 eq.), $[Rh(COD)Cl]_2$ (0.01 eq.), KOH (0.2 eq.), and a corresponding aryl pinacol ester (1.50 eq.) under nitrogen atmosphere, Then, 1,4-dioxane (0.20 M) was added and the resulted mixture was degassed. Degassed water was then added, and the mixture was placed into a pre-heated heating plate (50 °C). The reaction mixture was heated to 80 °C and stirred for 3 to 7 h upon complete consumption of the carbasugar mimic. TLC was used for monitoring the reaction progress. Then, the reaction was cooled down to ambient temperature and was diluted with water. The aqueous phase was extracted three times with EtOAc and the combined organic layers were washed with brine, dried over sodium sulfate, and concentrated under reduced pressure. The obtained crude product was purified by flash chromatography to obtain the desired product **12**, **13**, **14**, **15** and **17**.

Ketone reduction general procedure C

Compounds **12**, **13**, **14**, **15** and **17** (1.00 eq.) were dissolved in anhydrous MeOH (0.20 M) and cooled down to 0 °C. Then, $NaBH_4$ (1.50 eq.) was added portion wise. The reaction mixture was stirred for 1 h at 0 °C (until the bubbling stopped). Upon full conversion indicated by TLC, cold water was added. The aqueous phase was extracted five times with CH_2Cl_2. The combined organic layers were dried over sodium sulfate and concentrated under reduced pressure. The obtained residue was purified with silica gel chromatography to afford the pure products **22**, **23**, **24**, **25** and **27**.

Nucleophilic substitution general procedure D

The alcohol **22** (1.00 eq.) was dissolved in dry CH_2Cl_2 (0.10 M). Then, dry pyridine (3.00 eq.) was added and cooled down to 0 °C. To the cooled solution Tf_2O (2.00 eq.) was added dropwise. The reaction mixture was stirred for 1 h at 0 °C. Then, the reaction mixture was quenched with cold water and extracted with CH_2Cl_2. The combined organic layers were dried over sodium sulfate and concentrated to complete dryness. 4-chloro-7*H*-pyrrolo[2,3-*d*]pyrimidine (1.20 eq.) was dissolved in dry DMF (0.16 M) and cooled down to 0 °C before NaH (2.00 eq.) was added portion wise. The resulted mixture was stirred for 15 min at ambient temperature before a corresponding triflate (1.00eq.) solution in DMF (0.16 M) was added dropwise. The mixture was stirred for 17 h at ambient temperature. Then, water was added carefully, and the resulted mixture was extracted with EtOAc. The combined organic layers were washed extensively with brine, dried over sodium sulfate, and concentrated under reduced pressure. The crude products were purified over silica to afford the target compounds **32**.

Mitsunobu type glycosylation general procedure E

To a cooled solution of compounds **23**, **24** and **25** (1.00 eq.) in dry THF (0.15 M) was added PPh_3 (2.00 eq.) followed by dropwise addition of DIAD (1.80 eq.) under nitrogen atmosphere. The resulted mixture was stirred for 30 min at 0 °C. Then, the corresponding nucleobase (1.40 eq.) was added at 0 °C. The reaction mixture was stirred for 17 h at ambient temperature. After 17 h, the reaction was diluted with saturated bicarbonate solution and extracted with EtOAc. The combined organic layers were washed with brine, dried over sodium sulfate, and concentrated under reduced pressure. The obtained residue was purified by flash chromatography to afford the target compounds **33**, **34**, and **35**.

Aromatic substitution general procedure F

A pressure flask was charged with compound **32**, **33**, **34**, and **35** (1.00 eq.). Then, a 2:1 mixture of ammonia/ 1,4-dioxane (0.20 m) was added, and the resulted mixture was

heated to 100 °C and stirred upon full consumption. After 24 h, the reaction was allowed to cool down to rt and concentrated to complete dryness. The crude products were purified over silica gel chromatography to afford the target compounds **46**, **47**, **48**, and **49**.

Deprotection general procedure G

A solution of **46**, **47**, **48**, **49**, and **51** (0.01 M) in H_2O/TFA (1:1) was stirred at room temperature for 4 h. The mixture was concentrated to dryness and the residue was purified by preparative HPLC to afford the products as white foams **70a-d** and **70f**.

((3aR,4R,6R,6aR)-6-(6-amino-9H-purin-9-yl)-2,2-dimethyltetrahydrofuro[3,4-d][1,3]dioxol-4-yl)methanol (1)

To a solution of adenosine (10.0 g, 37.4 mmol, 1 equiv.) in acetone (1 L), was added $CH(OEt)_3$ (31.1 mL, 187 mmol, 5 equiv.) and *p*TsOH (35.6 g, 187 mmol, 5 equiv.) . The mixture was stirred at rt for 16 h, then quenched with aqueous saturated $NaHCO_3$, concentrated until a precipitate was obtained. The precipitate was dissolved in MeOH/CH_2Cl_2, filtered, and evaporated to give the final product (10.5 g, 34.4 mmol, 92%) as a white solid. ^{1}H NMR (500 MHz, DMSO-d_6) δ 8.33 (s, 1H), 8.15 (s, 1H), 7.33 (s, 2H), 6.11 (d, J = 3.1 Hz, 1H), 5.33 (dd, J = 6.2, 3.1 Hz, 1H), 5.29 (d, J = 21.9 Hz, 1H), 4.96 (dd, J = 6.2, 2.5 Hz, 1H), 4.20 (td, J = 4.8, 2.5 Hz, 1H), 3.59 – 3.48 (m, 2H), 1.53 (d, J = 0.7 Hz, 3H), 1.31 (d, J = 0.8 Hz, 3H). ^{13}C NMR (101 MHz, DMSO) δ 156.1, 152.5, 148.8, 139.6, 119.0, 113.0, 89.5, 86.3, 83.2, 81.3, 61.5, 27.0, 25.1. R_f: 0.31 (4 % MeOH in CH_2Cl_2; + 2 % NH_3 7 M in MeOH). HRMS (ESI): calcd. for $C_{13}H_{17}N_5O_4$ $[M+H]^+$: 308.13 found: 308.13.

9-((3a*R*,4*R*,6*R*,6a*R*)-6-(azidomethyl)-2,2-dimethyltetrahydrofuro[3,4-*d*][1,3]dioxol-4-yl)-9*H*-purin-6-amine (2)

To a solution of ((3a*R*,4*R*,6*R*,6a*R*)-6-(6-amino-9*H*-purin-9-yl)-2,2-dimethyltetrahydro-furo[3,4-*d*][1,3]dioxol-4-yl)methanol (6.50 g, 21.2 mmol, 1 equiv.) in dioxane (70 mL) at 0 °C were added DPPA (9.12 mL, 42.3 mmol, 2 equiv.) and DBU (9.50 mL, 63.5

mmol, 3 equiv.). The mixture was stirred at rt for 16 h. NaN_3 (6.88 g, 106 mmol, 5 equiv.) and 15-crown-5 (4.66 g, 21.2 mmol, 1 equiv.) were added and the mixture was stirred at 110 °C for 6 h. The organic phase was evaporated, water was added and the aqueous phase was extracted three times with EtOAc. The combined organics were washed with brine, dried over Na_2SO_4 and evaporated. The residue was purified on silica gel column eluting with 30-100% AcOEt in PE to afford the desired product (6.43 g, 19.3 mmol, 91%) as a light yellow solid. ^1H NMR (400 MHz, $CDCl_3$) δ 8.35 (s, 1H), 7.91 (s, 1H), 6.11 (d, J = 2.4 Hz, 1H), 6.06 – 5.91 (m, 2H), 5.46 (ddd, J = 6.4, 2.3, 0.4 Hz, 1H), 5.06 (ddd, J = 6.4, 3.5, 0.5 Hz, 1H), 4.45 – 4.31 (m, 1H), 3.66 – 3.45 (m, 2H), 1.61 (d, J = 0.7 Hz, 3H), 1.39 (d, J = 0.7 Hz, 3H). ^{13}C NMR (101 MHz, $CDCl_3$) δ 155.8, 153.2, 149.3, 140.0, 120.4, 114.8, 90.8, 85.7, 84.1, 82.2, 52.4, 27.2, 25.4. R_f: 0.41 (95:5 CH_2Cl_2/MeOH). HRMS (ESI): m/z $[M+H]^+$ calcd. for $C_{13}H_{17}N_8O_3$: 333.13 found: 333.13.

***tert*-butyl (9-((3aR,4R,6R,6aR)-6-(azidomethyl)-2,2-dimethyltetrahydrofuro[3,4-d][1,3]dioxol-4-yl)-9H-purin-6-yl)carbamate (3)**

To a solution of **2** (2.47 g, 7.4 mmol, 1 eq) in dry THF (C = 0.3 M, V = 25 mL) at 0°C under argon was added portionwise 60 % suspended NaH in oil (744 mg, 18 mmol, 2.5 eq). After 45 min at room temperature, Boc_2O was added (1.776 g, 8.14 mmol, 1.1 eq) in 3 mL dry THF at 0°C. After 1 h 30 min at room temperature, Boc_2O (807 mg, 3.7 mmol, 0.5 eq) was added at 0°C and the reaction was stirred at room temperature for 1 h 30 min. After that, reaction was quenched with NaCl at 0°C, extracted with AcOEt. The combined organics were washed with brine, dried over Na_2SO_4 and evaporated. The residue was purified on silica gel column eluting with 10–100% AcOEt in cyclohexane to afford the desired product (661 mg, 1.53 mmol, 80%) as a light yellow solid**.** ^1H NMR (400 MHz, $CDCl_3$) δ 8.77 (s, 2H), 8.09 (s, 2H), 6.15 (d, J = 2.4 Hz, 1H), 5.46 (dd, J = 6.4, 2.4 Hz, 1H), 5.05 (dd, J = 6.5, 3.6 Hz, 2H), 3.58 (d, J = 5.5 Hz, 2H), 1.63 (d, J = 0.7 Hz, 3H), 1.54 (s, 9H), 1.39 (d, J = 0.7 Hz, 3H). R_f: 0.76 (Ethyl Acetate). HRMS (ESI) : m/z $[M+H]^+$ calcd. for $C_{18}H_{25}N_8O_5$: 433.19 found: 433.19.

***tert*-butyl (9-((3aR,4R,6R,6aR)-6-(aminomethyl)-2,2-dimethyltetrahydrofuro[3,4-d][1,3]dioxol-4-yl)-9H-purin-6-yl)carbamate (4)**

To a solution of **3** (2.56 g, 5.9 mmol, 1eq) in AcOEt/MeOH (1:1, 19 mL) was added Pd/C (256 mg, 10 % w/w). The suspension was put under H_2 and stirred for 16 h at room temperature. The solution was then filtrated on celite and evaporated to afford the desired product as a grey foam (2.39 g, quant). **^{1}H NMR** (300 MHz, $CDCl_3$) δ 8.75 (s, 1H), 8.06 (s, 1H), 7.96 (d, *J* = 12.3 Hz, 1H), 6.07 (d, *J* = 3.0 Hz, 1H), 5.47 (dd, *J* = 6.5, 3.1 Hz, 1H), 5.02 (dd, *J* = 6.5, 3.5 Hz, 1H), 4.27 (ddd, *J* = 6.0, 4.5, 3.5 Hz, 1H), 3.10 – 2.88 (m, 2H), 1.63 (d, *J* = 0.7 Hz, 3H), 1.57 (s, 9H), 1.39 (d, *J* = 0.7 Hz, 3H). **R_f**: 0.42 (95:5 CH_2Cl_2/MeOH + 1 % Et_3N). **HRMS** (ESI) : m/z $[M +H]^+$ calcd. for $C_{18}H_{27}N_6O_5$: 407.20 found: 407.20.

***tert*-butyl (*tert*-butoxycarbonyl)-*L*-homoserinate (5)**

To a solution of (S)-4-(tert-butoxy)-3-((tert-butoxycarbonyl)amino)-4-oxobutanoic acid (500 mg, 1.73 mmol, 1 eq) and Et_3N (265 mL, 1.90 mmol, 1.1 eq) in dry THF (6.9 mL) was added isobutyl chloroformate (247 mL, 1.90 mmol, 1.1 equiv.) at -5 °C. The mixture was stirred for 30 min, then filtered. The solution containing (*S*)-(*S*)-4-(*tert*-butoxy)-3-((*tert*-butoxycarbonyl)amino)-4-oxobutanoic (ethyl carbonic) anhydride was recovered, cooled at 0 °C, MeOH (6.9 mL) and $NaBH_4$ (131 mg, 3.46 mmol, 2 equiv.) were added portionwise. The solution was then stirred at rt for 2 h. The reaction was quenched with diluted ammonium chloride, extract three times with EtOAc. The combined organics were washed with brine, dried over Na_2SO_4 and evaporated. The residue was purified on silica gel column eluting with 20-100% AcOEt in cyclohexane to afford the desired product (345 mg, 0.77 mmol, 45%) as a colorless oil. ^{1}H NMR (400 MHz, $CDCl_3$) δ 5.33 (d, *J* = 7.8 Hz, 1H), 4.42 – 4.28 (m, 1H), 3.80 – 3.57 (m, 2H), 3.44 (br s, 1H), 2.12 (ddt, *J* = 17.7, 12.8, 7.0 Hz, 1H), 1.69-1.49 (m, 1H), 1.47 (s, 9H), 1.45 (s, 9H). ^{13}C NMR (101 MHz, $CDCl_3$) δ 172.1, 156.7, 82.4, 80.4, 58.4, 51.1, 36.7, 28.4, 28.1. R_f: 0.26 (67:33 cyclohexane/AcOEt). HRMS (ESI) : m/z $[M +H]^+$ calcd. for $C_{13}H_{26}NO_5$: 276.17 found: 276.18.

***tert*-butyl (S)-2-((*tert*-butoxycarbonyl)amino)-4-oxobutanoate (6)**

To a solution of oxalyl chloride (1.17 mL, 13.6 mmol, 1.5 equiv.) in dry CH_2Cl_2 (30 mL) at -78 °C was added DMSO (2.15 mL, 18.2 mmol, 2 equiv.) in CH_2Cl_2 (5 mL). After 15 min, *tert*-butyl (*tert*-butoxycarbonyl)-*L*-homoserinate (2.50 g, 9.08 mmol, 1 equiv.) in CH_2Cl_2 (10 mL) was added. After 30 min, Et_3N was added (6.30 mL, 45.4 mmol, 5 equiv.) and the mixture was allowed to return at rt. The organic phase was washed with a solution of citric acid (5% w/w), then aqueous saturated $NaHCO_3$, brine, dried over Na_2SO_4, evaporated. The residue was purified on silica gel column (10-100% AcOEt in cyclohexane) to afford the desired product (1.96 g, 79%) as a colorless oil. ^{1}H NMR (400MHz, $CDCl_3$) 9.75 (s, 1H), 5.35 (m, 1H), 4.42 (m, 1H), 2.84-3.06 (m, 2H), 1.4 (s, 18H). ^{13}C NMR (101 MHz, $CDCl_3$) δ 199.3, 170.0, 155.4, 82.7, 80.1, 49.5, 46.4, 28.4, 27.9. R_f: 0.44 (76:33 cyclohexane/AcOEt). HRMS (ESI) : m/z $[M +H]^+$ calc. for $C_{13}H_{24}NO_5$: 274.16 found: 274.16.

***tert*-butyl (*S*)-2-((*tert*-butoxycarbonyl)amino)-4-((((3a*R*,4*R*,6*R*,6a*R*)-6-(6-((*tert*-butoxycarbonyl)amino)-9*H*-purin-9-yl)-2,2-dimethyltetrahydrofuro[3,4-*d*][1,3]dioxol-4-yl)methyl)amino)butanoate (7)**

To a stirred solution of **4** (3.79 g, 9.23 mmol, 1.1 eq.) and aldehyde **6** (2.32 g, 8.39 mmol, 1 eq.) in dry DCE (0.12 m based on the aldehyde) was added AcOH (533 µL, 9.23 mmol, 1.1 eq.). The solution was stirred for 4 h at rt, then $NaBH(OAc)_3$ (4.67 g, 21.82 mmol, 2.6 eq.) was added and the mixture was stirred for 4 h at rt. After completion, the reaction was quenched by the addition of a 5 % aq. $NaHCO_3$ solution and the phases were separated. The aqueous phase was then extracted 3 times with CH_2Cl_2 and the combined organic phases once with brine. Drying over Na_2SO_4, filtration and evaporation afforded the crude product that was subjected to silica gel column chromatography eluting with CH_2Cl_2/MeOH (99.5:0.5–90.5:9.5) to afford the secondary amine **7** (2.4 g, 3.6 mmol, 43 %). $C_{31}H_{49}N_7O_9$ (663.77 g/mol). ^{1}H-NMR (400 MHz; DMSO-d_6): δ 10.14 (bs, 1H, N<u>*H*</u> carbamate nucleobase), 8.62 (s, 1H, H2), 8.61 (s, 1H, H8), 7.15 (d, ^3J = 8.0 Hz, 1H, N<u>*H*</u> carbamate), 6.17 (d, ^3J = 2.7 Hz, 1H,

H1'), 5.47 (dd, ^3J = 6.0 Hz, 2.7 Hz, 1H, H2'), 4.99 (dd, ^3J = 6.0 Hz and ^3J = 2.7 Hz, 1H, H3'), 4.22 (td, ^3J = 5.6 Hz and ^3J = 2.8 Hz, 1H, H4'), 3.94–3.87 (m, 1H, Hα), 2.73 (dd, ^2J = 12.4 Hz and ^3J = 5.8Hz, 1H, H5$_A$'), 2.61 (dd, ^2J = 12.4 Hz and ^3J = 5.8 Hz, 1H, H5$_B$'), 2.56–2.45 (m, 2H, Hγ), 1.75–1.71 (m, 1H, Hβ$_A$), 1.66-1.60 (m, 1H, Hβ$_B$), 1.55 (s, 3H, C*H*$_3$), 1.47 (s, 9H, C*H*$_3$ *t*Bu), 1.41–1.29 (m, 22H, C*H*$_3$, 2 x C*H*$_3$ *t*Bu); HRMS (ESI): calcd. for $C_{31}H_{50}N_7O_9$ [M+H]$^+$: 664.3665, found: 664.3661.

***tert*-butyl (S)-2-((tert-butoxycarbonyl)amino)-4-((((3aR,4R,6R,6aR)-6-(6-((tert-butoxycarbonyl)amino)-9H-purin-9-yl)-2,2-dimethyltetrahydrofuro[3,4-d][1,3]dioxol-4-yl)methyl)(3-((tert-butoxycarbonyl)amino)propyl)amino)butanoate (8a) & (*S*)-2-amino-4-((((2*R*,3*S*,4*R*,5*R*)-5-(6-amino-9*H*-purin-9-yl)-3,4-dihydroxytetrahydrofuran-2-yl)methyl)(3-aminopropyl)amino)butanoic acid (1a, 9a)**

Reductive amination (**8a**): yield: 89.6 mg, 0.109 mmol, 40 %. HRMS (ESI): calcd. for $C_{39}H_{65}N_8O_{11}$ [M+H]$^+$: 821.4767, found: 821.4766; R*f*: 0.72 (CH_2Cl_2/MeOH 9:1). Deprotection (**1a**, **9a**): yield: 75 mg, 0.098 mmol, 100 % (3 TFA salt). ^{1}H-NMR (400 MHz; DMSO-*d*$_6$): *δ* 8.52 (s, 1H, H2), 8.49–8.27 (m, 4H, H8, N*H*$_3^+$), 7.93 (bs, 3H, N*H*$_3^+$), 6.01 (d, ^3J = 4.8 Hz, 1H, H1'), 4.64 (t, ^3J = 4.8 Hz, 1H, H2'), 4.41–4.31 (m, 1H, H4'), 4.23 (t, ^3J = 4.8 Hz, 1H, H3'), 4.01 (t, ^3J = 6.0 Hz, 1H, Hα), 3.70–3.58 (m, 1H, H5'$_A$), 3.53–3.47 (m, 1H, H5'$_B$), 3.32–3.24 (m, 2H, Hγ), 3.23–3.12 (m, 2H, H1''), 2.91–2.78 (m, 2H, H3''), 2.30–2.17 (m, 1H, Hβ$_A$), 2.15–2.04 (m, 1H, Hβ$_B$), 1.97–1.84 (m, 2H, H2''); HRMS (ESI): calc. for $C_{17}H_{29}N_8O_5$ [M+H]$^+$: 425.2255, found: 425.2255; HPLC: t_R = 1.684 min (Method A). UV-purity at 254 nm = 95.6%.

***tert*-butyl (3-(4,4,5,5-tetramethyl-1,3,2-dioxaborolan-2-yl)phenyl)carbamate (10)**

2190 mg (10.00 mmol; 1.00 eq) of 3-(4,4,5,5-tetramethyl-1,3,2-dioxaborolan-2-yl)aniline were dissolved in 50 mL of DMF. 2728 mg (12.50 mmol; 1.25 eq) of Boc_2O and 2.77 mL (20.00 mmol; 2.00 eq) of Et_3N were added. The reaction mixture was heated at 90 °C overnight. The solvent was removed under reduced pressure and the residue was purified by column chromatography (Petrolether/EtOAc 99:1% to 65:35%) to give 2380 mg (7.45 mmol; 75%) of the title compound in the form of a colorless

solid. R_f = 0.80 (Petrolether/EtOAc 3:1). ^{1}H-NMR (400 MHz, DMSO-d_6) δ = 9.32 (s, 1H), 7.90 (s, 1H), 7.48-7.42 (m, 1H), 7.27-7.22 (m, 2H), 1.47 (s, 9H), 1.29 (s, 12H) ppm.

***tert*-butyl N-{2-[3-(4,4,5,5-tetramethyl-1,3,2-dioxaborolan-2-yl)phenyl]ethyl}carba-mate (11)**

Into a 40 mL vial were added tert-butyl N-[2-(3-bromophenyl)ethyl]carbamate (1 g, 3.3 mmol, 1.0 eq.), 4,4,5,5-tetramethyl-2-(tetramethyl-1,3,2-dioxaborolan-2-yl)-1,3,2-dioxa-borolane (1.27 g, 5.0 mmol, 1.5 eq), KOAc (0.65 g, 6.6 mmol, 2.0 eq) and Pd(dppf)Cl_2 (0.24 g, 0.33 mmol, 0.1 eq) in dioxane (10.0 mL). The reaction was stirred for 2 h at 100 °C, then concentrated. The residue was dissolved in DCM (4.0 mL) and was purified by silica gel column chromatography (3:1 Petroleum ether:AcOEt) to afford tert-butyl N-{2-[3-(4,4,5,5-tetramethyl-1,3,2-dioxaborolan-2-yl)phenyl]ethyl}carbamate (0.6 g, 52%) as a white solid. LCMS (conditions XBridge C18, 50*3.0 mm, 3.5 µm, 5% - 100% Acetonitrile / 5 mM aqueous NH_4HCO_3 - 2.5 min, 1.50 mL/min,): T_R = 1.89 min; ES m/z $[M+H]^+$: 348.

(6*R*)-2,2-dimethyl-6-phenyltetrahydro-4*H*-cyclopenta[*d*][1,3]dioxol-4-one (12)

Following the general procedure **B** for [Rh]-catalyzed 1,4-addition, compound **12** was obtained starting from (3a*R*,6a*R*)-2,2-dimethyl-3a,6a-dihydro-4*H*-cyclopenta[*d*][1,3]dioxol-4-one (1.00 g, 6.16 mmol), 4,4,5,5-tetramethyl-2-phenyl-1,3,2-dioxaborolane (1.95 g, 9.24 mmol), $[Rh(COD)Cl]_2$ (0.31 g, 0.62 mmol), and KOH (0.07 g, 1.23 mmol) in dry degassed 1,4-dioxane (0.15 M, 41.10 mL) and degassed water (5.00 M, 1.20 mL) after column chromatography on silica (cyclohexane/EtOAc; 0-30%) as yellowish resin (1.14 g, 80%). R_f = 0.57 (cyclohexane/EtOAc; 30%). ^{1}H NMR (400 MHz, DMSO-*d6*) *δ* 7.38 – 7.32 (m, 2H, *m*-Ar), 7.29 – 7.25 (m, 1H, *p*-Ar), 7.25 – 7.21 (m, 2H, *o*-Ar), 4.72 (dd, *J* = 5.8, 2.5 Hz, 1H, H1´), 4.57 (dt, *J* = 5.8, 0.9 Hz, 1H, H2´), 3.55 (ddd, *J* = 8.7, 5.8, 2.6 Hz, 1H, H3´), 3.02 – 2.90 (m, 1H, H4´), 2.54 (dd, *J* = 5.7, 1.7 Hz, 1H, H4´), 1.42 – 1.39 (m,

3H, C*H*$_3$, acetonide), 1.28 (d, *J* = 0.7 Hz, 3H, C*H*$_3$, acetonide). APCI: calc. for $C_{14}H_{16}O_3$ $[M+H]^+$: 233.11, found: 233.2 $[M+H]^+$.

(3a*R*,6*R*,6a*R*)-2,2-dimethyl-6-(pyridin-4-yl)tetrahydro-4*H*-cyclopenta[*d*][1,3]dioxol-4-one (13)

Following the general procedure **B** for [Rh]-catalyzed 1,4-addition, compound **13** was obtained starting from (3a*R*,6a*R*)-2,2-dimethyl-3a,6a-dihydro-4*H*-cyclopenta[*d*][1,3]dioxol-4-one (1.00 g, 6.16 mmol), 4-(4,4,5,5-tetramethyl-1,3,2-dioxaborolan-2-yl)pyridine (1.95 g, 9.24 mmol), $[Rh(COD)Cl]_2$ (0.31 g, 0.62 mmol), and KOH (0.07 g 1.23 mmol) in dry degassed 1,4-dioxane (0.15 M, 41.10 mL) and degassed water (5.00 M, 1.20 mL) after column chromatography on silica (cyclohexane/EtOAc; 100%) as yellowish resin (1.10 g, 76%). R_f = 0.37 (cyclohexane/EtOAc; 100%). ^1H NMR (400 MHz, DMSO-*d*6) δ 8.55 – 8.51 (m, 2H, H2/6 pyridine), 7.33 – 7.28 (m, 2H, H3/5, pyridine), 4.75 (dd, *J* = 5.9, 3.1 Hz, 1H, H1′), 4.60 (dt, *J* = 5.9, 0.9 Hz, 1H, H2′), 3.56 (ddd, *J* = 9.4, 6.7, 3.1 Hz, 1H, H3′), 3.01 – 2.89 (m, 1H), 2.60 (ddd, *J* = 18.3, 6.8, 1.6 Hz, 1H, H4′), 1.41 (s, 3H, C*H*$_3$, acetonide), 1.29 (s, 3H, C*H*$_3$, acetonide). APCI: calc. for $C_{13}H_{16}NO_3$ $[M+H]^+$: 234.11, found: 234.3 $[M+H]^+$.

(3a*R*,6*S*,6a*R*)-2,2-dimethyl-6-(thiophen-2-yl)tetrahydro-4*H*-cyclopenta[*d*][1,3]dioxol-4-one (14)

Following the general procedure **B** for [Rh]-catalyzed 1,4-addition, compound **14** was obtained starting from (3a*R*,6a*R*)-2,2-dimethyl-3a,6a-dihydro-4*H*-cyclopenta[*d*][1,3]dioxol-4-one (1.00 g, 6.16 mmol), 4,4,5,5-tetramethyl-2-(thiophen-2-yl)-1,3,2-dioxaborolane (2.00 g, 9.24 mmol), $[Rh(COD)Cl]_2$ (0.16 g, 0.31 mmol), and KOH (0.07 g, 1.23 mmol) in dry degassed 1,4-dioxane (0.15 M, 41.10 mL) and degassed water (5.00 M, 1.20 mL) after column chromatography on silica (cyclohexane/EtOAc; 0-30%) as colorless resin (320 mg, 22%). R_f = 0.27 (cyclohexane/EtOAc; 20%). ^1H NMR (400 MHz, DMSO-*d*6) δ ^1H NMR

(400 MHz, DMSO-d_6) δ 7.45 (dd, *J* = 5.1, 1.2 Hz, 1H, H5 thiophene), 7.01 – 6.98 (m, 1H, H4 thiophene), 6.95 (dt, *J* = 3.5, 1.1 Hz, 1H, H3 thiophene), 4.82 (dd, *J* = 5.7, 2.3 Hz, 1H, H1′), 4.46 (ddt, *J* = 5.7, 1.6, 0.8 Hz, 1H, H2′), 3.84 – 3.78 (m, 1H, H3′), 3.09 – 3.01 (m, 1H, H4′), 2.58 – 2.51 (m, 1H, H4′), 1.39 (s, 3H, C$\underline{H}_3$, acetonide), 1.30 (s, 3H, C$\underline{H}_3$, acetonide). APCI: calc. for $C_{12}H_{15}O_3S$ $[M+H]^+$: 239.07, found: 239.1 $[M+H]^+$.

(3a*R*,6*R*,6a*R*)-2,2-dimethyl-6-(1*H*-pyrazol-5-yl)tetrahydro-4*H*-cyclopenta[*d*][1,3]dioxol-4-one (15)

Following the general procedure **B** for [Rh]-catalyzed 1,4-addition, compound **15** was obtained starting from (3a*R*,6a*R*)-2,2-dimethyl-3a,6a-dihydro-4*H*-cyclopenta[*d*][1,3]dioxol-4-one (0.80 g, 4.93 mmol), *tert*-butyl 5-(4,4,5,5-tetramethyl-1,3,2-dioxaborolan-2-yl)-1*H*-pyrazole-1-carboxylate (2.24 g, 7.39 mmol), $[Rh(COD)Cl]_2$ (0.25 g, 0.49 mmol), and KOH (0.06 g, 0.99 mmol) in dry degassed 1,4-dioxane (0.15 M, 32.90 mL) and degassed water (5.00 M, 1.00 mL) after column chromatography on silica (cyclohexane/EtOAc, 0-60%) as colorless resin (500 mg, 46%). Under standard conditions, the boc group was cleaved off. ^1H NMR (400 MHz, DMSO-*d6*) *δ* 7.91 (dd, *J* = 2.3, 0.6 Hz, 1H, H3 pyrazol), 7.49 (d, *J* = 1.8 Hz, 1H, H4 pyrazol), 6.27 (t, *J* = 2.1 Hz, 1H, N*H* pyrazol), 5.17 (d, *J* = 7.6 Hz, 1H, H1′), 4.72 – 4.65 (m, 1H, H2′), 4.50 – 4.45 (m, 1H, H3′), 3.14 (dd, *J* = 18.1, 7.5 Hz, 1H, H4′), 2.39 – 2.31 (m, 1H, H4′), 1.40 (s, 3H, C$\underline{H}_3$, acetonide), 1.28 (s, 3H, C$\underline{H}_3$, acetonide). APCI: calc. for $C_{11}H_{15}N_2O_3$ $[M+H]^+$: 223.10, found: 223.2 $[M+H]^+$.

(3aR,6R,6aR)-2,2-Dimethyl-6-[1-(oxan-2-yl)pyrazol-4-yl]-tetrahydrocyclopenta[d] [1,3]dioxol-4-one (16)

Into a 40 mL vial were added (3aR,6aR)-2,2-dimethyl-3aH,6aH-cyclopenta[d][1,3]dioxol-4-one (0.5 g, 3.2 mmol, 1.0 eq), 1-(oxan-2-yl)-4-(4,4,5,5-tetramethyl-1,3,2-dioxaborolan-2-yl)pyrazole (1.35 g, 4.9 mmol, 1.5 eq), acetylacetonato bis(ethylene)rhodium(I) (83.7 mg, 0.32 mmol, 0.1 eq) and *R*-BINAP

(260 mg, 0.32 mmol, 0.1 eq) in dioxane (5 mL) at room temperature. The reaction was stirred for 2h at 100 °C, then concentrated. The residue was dissolved in DCM (3 mL) and was purified by silica gel column chromatography (2:1 Petroleum ether:AcOEt) to afford (3aR,6R,6aR)-2,2-dimethyl-6-[1-(oxan-2-yl)pyrazol-4-yl]-tetrahydrocyclopenta[d][1,3]dioxol-4-one **16** (0.5 g, 55%) as a white solid. LCMS (conditions: XBridge C18, 50*3.0 mm, 3.5 µm, Mobile Phase A: Water/5mM NH_4HCO_3, Mobile Phase B: Methanol, 10% - 95% B - 2 min, 1.50 mL/min): T_R = 1.28 min; ES m/z $[M+H]^+$: 307.

***tert*-butyl (3-((3ar,4r,6ar)-2,2-dimethyl-6-oxotetrahydro-4*H*-cyclopenta[d][1,3]dioxol-4-yl)phenyl)carbamate (17)**

Following the general procedure **B** for [Rh]-catalyzed 1,4-addition, compound **17** was obtained starting from (3a*R*,6a*R*)-2,2-dimethyl-3a,6a-dihydro-4*H*-cyclopenta[*d*][1,3]dioxol-4-one (1.04 g, 6.77 mmol), compound **10** (2.38 g, 7.45 mmol), $[Rh(COD)Cl]_2$ (0.34 g, 0.68 mmol), and KOH (0.08 g, 1.35 mmol) in dry degassed 1,4-dioxane (0.15 M, 45.13 mL) and degassed water (5.00 M, 1.35 mL) after column chromatography on silica (cyclohexane/EtOAc; 0-50%) as yellowish resin (1.10 g, 3.16 mmol). The product was used without further characterization. TLC: R_f = 0.76 (Cyclohexane/EtOAc 1:1).

***tert*-butyl (3-((3aR,4R,6aR)-2,2-dimethyl-6-oxotetrahydro-4H-cyclopenta[d][1,3]diox-ol-4-yl)benzyl)carbamate (18)**

Into a 40 mL vial were added (3aR,6aR)-2,2-dimethyl-3aH,6aH-cyclopenta[d][1,3]dioxol-4-one (0.5 g, 3.2 mmol, 1.0 eq), 3-{[(tert-butoxycarbonyl)amino]methyl}phenylboronic acid (1.6 g, 6.5 mmol, 2.0 eq), acetylacetonato bis(ethylene)rhodium(I) (84.0 mg, 0.3 mmol, 0.1 eq), (*R*)-BINAP (0.2 g, 0.3 mmol, 0.1 eq), dioxane (5.0 mL) and H_2O (0.5 mL) at room temperature under N_2 atmosphere. The reaction mixture was stirred at 100°C for 2 h, then allowed to cool to room temperature. The resulting mixture was concentrated under reduced pressure.

The residue was extracted with AcOEt (3 x 20.0 mL). The combined organic layers were washed with brine and dried over anhydrous Na_2SO_4. After filtration, the filtrate was concentrated under reduced pressure. The residue was dissolved in DCM (3.0 mL) and purified by silica gel column chromatography (70:30 Petroleum ether/THF) to afford tert-butyl (3-((3aR,4R,6aR)-2,2-dimethyl-6-oxotetrahydro-4H-cyclopenta[d][1,3]dioxol-4-yl)benzyl)carbamate **18** (350 mg, 29%) as yellow oil. LCMS (conditions: L-column3 ODS, 50*3.0 mm, 3.0 μm, Mobile Phase A: Water/5mM NH_4HCO_3+0.05% Ammonia, Mobile Phase B: Acetonitrile, 5%-100%B – 2 min, 1.50 L/min): T_R = 1.45 min; ES m/z $[M-H]^-$: 360.

***tert*-butyl N-(2-{3-[(3aR,4R,6aR)-2,2-dimethyl-6-oxo-tetrahydrocyclopenta[d][1,3]di-oxol-4-yl]phenyl}ethyl)carbamate (19)**

Into a 40 mL vial were added (3aR,6aR)-2,2-dimethyl-3aH,6aH-cyclopenta[d][1,3]dioxol-4-one (0.18 g, 1.15 mmol, 1.0 eq), tert-butyl N-{2-[3-(4,4,5,5-tetramethyl-1,3,2-dioxaborolan-2-yl)phenyl]ethyl}carbamate **11** (0.6 g, 1.7 mmol, 1.5 eq), acetylacetonato bis(ethylene)rhodium(I) (29.64 mg, 0.12 mmol, 0.1 eq) and R-BINAP (91.2 mg, 0.12 mmol, 0.1 eq) in dioxane (8.0 mL) and H_2O (0.8 mL). The reaction was stirred for 3 h at 100 °C, then cooled and quenched with water (10 mL). This was extracted with EtOAc (3x15 mL). The organic layer was dried over anhydrous Na_2SO_4, filtered and the filtrate was concentrated. The residue was dissolved in DCM (3 mL) and was purified by silica gel column chromatography (2:1 Petroleum ether:AcOEt) to afford tert-butyl N-(2-{3-[(3aR,4R,6aR)-2,2-dimethyl-6-oxo-tetrahydrocyclopenta[d][1,3]dioxol-4-yl]phenyl}ethyl)carbamate **19** (0.36 g, 84%) as a white solid. LCMS (conditions XBridge C18, 50*3.0 mm, 3.5 μm, 5% - 100% Acetonitrile / 5 mM aqueous NH_4HCO_3 - 2 min, 1.50 mL/min,): T_R = 1.44 min; ES m/z $[M+H]^+$: 376.

(3aR,6R,6aR)-6-(3-(hydroxymethyl)phenyl)-2,2-dimethyltetrahydro-4H-cyclopenta[d][1,3]dioxol-4-one (20)

A solution of (3aR,6aR)-2,2-dimethyl-3aH,6aH-cyclopenta[d][1,3]dioxol-4-one (3.0 g, 19.5 mmol, 1.0 eq), 3-(hydroxymethyl)phenylboronic acid (3.8 g, 25.3 mmol, 1.3 eq) and R-BINAP (1.2g, 1.95 mmol, 0.1 eq), acetylacetonato bis(ethylene)rhodium(I) (546 mg, 1.95 mmol, 0.1 eq) in 1,4-dioxane (30.0 mL) and H_2O (3 mL) was stirred for 2 h at 100 °C under nitrogen atmosphere. The resulting mixture was concentrated under reduced pressure. The resulting mixture was extracted with AcOEt (3 x 50.0 mL). The combined organic layers were washed with brain, dried over anhydrous Na_2SO_4. After filtration, the filtrate was concentrated under reduced pressure. The residue was dissolved in DCM (5.0 mL) and purified by silica gel column chromatography (70:30 Petroleum ether/THF) to afford (3aR,6R,6aR)-6-(3-(hydroxymethyl)phenyl)-2,2-dimethyltetrahydro-4H-cyclopenta[d][1,3]dioxol-4-one **20** (3.5 g, 74 %) as a white oil. LCMS (conditions L-column3 C18, 50*3.0 mm, 3.0 um, Mobile Phase A: Water/5mM NH_4HCO_3, Mobile Phase B: Methanol, 10%-95%B-2 min, ES, m/z): T_R =1.28 min; $[M-H]^-$: 261.

4H-cyclopenta[d][1,3]dioxol-4-one (21)

Into a 100 mL vial were added (3aR,6R,6aR)-6-(3-(hydroxymethyl)phenyl)-2,2-dimethyltetrahydro-4H-cyclopenta[d][1,3]dioxol-4-one **20** (3.5 g, 13.3 mmol, 1.0 eq) and Imidazole (1.36g, 20 mmol, 1.5 eq), t-butyldimethylchlorosilane (3.0 g, 20 mmol, 1.5 eq) in DMF (30.0 mL) at 0 °C. The resulting mixture was stirred for additional 2 h at room temperature. The resulting mixture was extracted with EtOAc (3 x 50.0 mL). The combined organic layers were washed with water (3x50.0 mL), dried over anhydrous Na_2SO_4. After filtration, the filtrate was concentrated under reduced pressure. The resulting mixture was diluted with DCM (8.0 mL) and purified by silica gel column chromatography (60:40 Petroleum ether/THF) to afford (3aR,6R,6aR)-6-(3-(((tert-butyldimethylsilyl)oxy)methyl)phenyl)-2,2-dimethyltetrahydro-4H-cyclopenta[d][1,3]dioxol-4-one **21** (4.1 g, 82%) as a white solid. LCMS (conditions L-

column3 C18, 50*3.0 mm, 3.0 um, Mobile Phase A: Water/5mM NH_4HCO_3, Mobile Phase B: Methanol, 10%-95%B-2min, ES, m/z): T_R =1.70 min; $[M-H]^-$: 375.

(3a*S*,4*S*,6*R*,6a*R*)-2,2-dimethyl-6-phenyltetrahydro-4*H*-cyclopenta[*d*][1,3]dioxol-4-ol (22)

Following the general procedure **C** for ketone reduction, compound **22** was obtained starting from compound **12** (1.13 g, 4.82 mmol), $NaBH_4$ (0.28 g, 7.22 mmol) in dry MeOH (0.20 M, 24.10 mL) after column chromatography on silica (cyclohexane/EtOAc; 0-50%) as colorless resin (954 mg, 85%). R_f = 0.22 (cyclohexane/EtOAc; 30%). ^{1}H NMR (400 MHz, DMSO-d_6) δ 7.33 – 7.28 (m, 2H, *m*-Ar), 7.22 – 7.17 (m, 3H, *o,p*-Ar), 4.60 (dd, *J* = 6.3, 2.3 Hz, 1H, H3′), 4.57 – 4.49 (m, 1H, H2′), 4.45 (d, *J* = 5.7 Hz, 1H, -O*H*), 4.04 – 3.95 (m, 1H, H1′), 3.19 (ddd, *J* = 7.5, 5.1, 2.3 Hz, 1H, H4′), 2.10 (dt, *J* = 12.5, 7.8 Hz, 1H, H5′), 1.79 (dt, *J* = 12.5, 5.2 Hz, 1H, H5′), 1.46 (s, 3H, CH_3, acetonide), 1.27 (s, 3H, CH_3, acetonide). APCI: calc. for $C_{14}H_{19}O_3$ $[M+H]^+$: 235.13, found: 235.2 $[M+H]^+$.

(3a*S*,4*S*,6*R*,6a*R*)-2,2-dimethyl-6-(pyridin-4-yl)tetrahydro-4*H*-cyclopenta[*d*][1,3]dioxol-4-ol (23)

Following the general procedure **C** for ketone reduction, compound **23** was obtained starting from compound **13** (1.09 g, 4.63 mmol), $NaBH_4$ (0.27 g, 6.94 mmol) in dry MeOH (0.20 M, 23.10 mL) after column chromatography on silica (CH_2Cl_2/MeOH; 0-10%) as colorless resin (862 mg, 79%). R_f = 0.22 (EtOAc; 100%). ^{1}H NMR (400 MHz, DMSO-d_6) δ 8.49 – 8.46 (m, 2H, H2, H6 pyridine), 7.25 – 7.22 (m, 2H, H3, H5 pyridine), 4.62 (dd, *J* = 6.2, 2.4 Hz, 1H, H3′), 4.56 (d, *J* = 5.6 Hz, 1H, -O*H*), 4.55 – 4.51 (m, 1H, H2′), 4.00 – 3.92 (m, 1H, H1′), 3.19 (ddd, *J* = 7.6, 5.1, 2.4 Hz, 1H, H4′), 2.12 (dt, *J* = 12.7, 7.9 Hz, 1H, H5′), 1.82 (dt, *J* = 12.5, 5.2 Hz, 1H, H5′), 1.46 (s, 3H, CH_3, acetonide), 1.28 (s, 3H, CH_3, acetonide). APCI: calc. for $C_{13}H_{18}NO_3$ $[M+H]^+$: 236.12, found: 236.3 $[M+H]^+$.

(3a*S*,4*S*,6*S*,6a*R*)-2,2-dimethyl-6-(thiophen-2-yl)tetrahydro-4*H*-cyclopenta[*d*][1,3]dioxol-4-ol (24)

Following the general procedure **C** for ketone reduction, compound **24** was obtained starting from compound **14** (0.32 g, 0.79 mmol), $NaBH_4$ (0.05 g, 1.19 mmol) in dry MeOH (0.20 M, 4.00 mL) after column chromatography on silica (cyclohexane/EtOAc; 0-50%) as colorless resin (265 mg, 84%). R_f = 0.72 (cyclohexane/EtOAc; 50%). ^{1}H NMR: (400MHz DMSO-d_6): δ 7.37 (dd, J = 5.1, 1.2 Hz, 1H, H5-thiophene), 6.95 (dd, J = 5.1, 3.5 Hz, 1H, H4 thiophene), 6.91 (dt, J = 3.5, 1.2 Hz, 1H, H3-thiophene), 4.50 – 4.45 (m, 1H, H1´), 4.05 – 3.96 (m, 1H, H3´), 3.42 – 3.24 (m, 1H, H4´), 2.17 – 2.06 (m, 1H. H5´), 1.89 (m, 1H, H5´), 1.47 – 1.41 (m, 3H, C<u>*H*</u>3), 1.27 (d, J = 0.7 Hz, 3H, C<u>*H*</u>3). APCI: calc. for $C_{12}H_{17}O_3S$ $[M+H]^+$: 241.08, found: 241.2 $[M+H]^+$.

(3a*S*,4*S*,6*R*,6a*R*)-2,2-dimethyl-6-(1*H*-pyrazol-5-yl)tetrahydro-4*H*-cyclopenta[*d*][1,3]dioxol-4-ol (25)

Following the general procedure **C** for ketone reduction, compound **25** was obtained starting from compound **15** (0.59 g, 1.81 mmol), $NaBH_4$ (0.10 g, 2.72 mmol) in dry MeOH (0.20 M, 9.10 mL) after column chromatography on silica (cyclohexane/EtOAc; 0-50%) as colorless resin (315 mg, 76%). R_f = 0.44 (cyclohexane/EtOAc; 50%). ^{1}H NMR (400 MHz, DMSO-*d*6) δ 8.70 (s, 1H, H2), 8.07 (d, *J* = 3.7 Hz, 1H, H6), 7.90 (dd, *J* = 2.3, 0.6 Hz, 1H, H3 pyrazol), 7.56 (dd, *J* = 1.8, 0.5 Hz, 1H, -N<u>*H*</u> pyrazol), 6.77 (d, *J* = 3.7 Hz, 1H, H5), 6.32 (dd, *J* = 2.3, 1.9 Hz, 1H, H4 pyrazol), 5.32 – 5.25 (m, 1H, H1´), 5.10 (dd, *J* = 7.7, 5.6 Hz, 1H, H2´), 5.02 (dd, *J* = 7.7, 5.4 Hz, 1H, H3´), 4.93 – 4.85 (m, 1H, H4´), 3.01 (q, *J* = 12.2 Hz, 1H, H5´), 2.76 – 2.66 (m, 1H, H5´), 1.56 (s, 3H, C<u>*H*</u>3, acetonide), 1.40 (s, 3H, C<u>*H*</u>3, acetonide). APCI: calc. for $C_{11}H_{17}N_2O_3$ $[M+H]^+$: 225.12, found: 225.0 $[M+H]^+$.

(3aS,4S,6R,6aR)-2,2-Dimethyl-6-[1-(oxan-2-yl)pyrazol-4-yl]-tetrahydro-3aH-cyclopenta[d][1,3]dioxol-4-ol (26)

Into a 40 mL vial were added (3aR,6R,6aR)-2,2-dimethyl-6-[1-(oxan-2-yl)pyrazol-4-yl]-tetrahydrocyclopenta[d][1,3]dioxol-4-one **16** (0.5 g, 1.8 mmol, 1.0 eq) in MeOH (5 mL), followed by $NaBH_4$ (0.17 g, 2.7 mmol, 1.5 eq) at 0 °C. The reaction was stirred for 0.5 h at room temperature, then concentrated. The residue was dissolved in DCM (3 mL) and was purified by silica gel column chromatography (3:2 Petroleum ether:AcOEt) to afford (3aS,4S,6R,6aR)-2,2-dimethyl-6-[1-(oxan-2-yl)pyrazol-4-yl]-tetrahydro-3aH-cyclopenta[d][1,3]dioxol-4-ol **26** (0.4 g, 79%) as a white solid. LCMS (conditions Xbridge Shield C18, 50*3.0 mm, 3.5 μm, Mobile Phase A: Water/0.05% Ammonia, Mobile Phase B: Acetonitrile, 5 - 100% B - 2 min, 1.50 mL/min): T_R = 0.89 min; ES m/z $[M+H]^+$: 309.

***tert*-butyl (3-((3a*R*,4*R*,6*S*,6a*S*)-6-hydroxy-2,2-dimethyltetrahydro-4*H*-cyclopenta[*d*]-[1,3]dioxol-4-yl)phenyl)carbamate (27)**

Following the general procedure **C** for ketone reduction, compound **27** was obtained starting from compound **17** (1.10 g, 3.16 mmol), $NaBH_4$ (0.18 g, 4.74 mmol) in dry MeOH (0.20 M, 15.80 mL) after column chromatography on silica (cyclohexane/EtOAc; 0-25%) as colorless resin (950 mg, 2.72 mmol), which was used without further characterization. R_f = 0.48 (Cyclohexane/EtOAc 1:1).

***tert*-butyl(3-((3aR,4R,6S,6aS)-6-hydroxy-2,2-dimethyltetrahydro-4H-cyclopenta[d][1,3]dioxol-4-yl)benzyl)carbamate (28)**

To a stirred solution of tert-butyl (3-((3aR,4R,6aR)-2,2-dimethyl-6-oxotetrahydro-4H-cyclopenta[d][1,3]dioxol-4-yl)benzyl)carbamate **18** (340 mg, 0.9 mmol, 1.0 eq) in MeOH (4.0 mL) was added $NaBH_4$ (36.0 mg, 0.9 mmol, 1.0 eq) in portions at 0°C. The reaction mixture was stirred at room temperature for 30 min then concentrated under reduced pressure. The residue was dissolved in DCM (5.0 mL), and purified by silica gel column chromatography (40:60 Petroleum ether/THF) to afford tert-butyl (3-

((3aR,4R,6S,6aS)-6-hydroxy-2,2-dimethyltetrahydro-4H-cyclopenta[d][1,3]dioxol-4-yl)benzyl)carbamate **28** (330 mg, 96%) as a clear oil. LCMS (conditions: L-column3 ODS, 50*3.0 mm, 3.0 µm, Mobile Phase A: Water/5mM NH_4HCO_3+0.05% Ammonia, Mobile Phase B: Acetonitrile, 5%-100%B - 2 min, 1.50 L/min): T_R = 1.38 min; ES m/z $[M-H]^-$: 362.

***tert*-butyl N-(2-{3-[(3aR,4R,6S,6aS)-6-hydroxy-2,2-dimethyl-tetrahydro-3aH-cyclope-nta[d][1,3]dioxol-4-yl]phenyl}ethyl)carbamate (29)**

Into an 8 mL vial was added tert-butyl N-(2-{3-[(3aR,4R,6aR)-2,2-dimethyl-6-oxo-tetrahydrocyclopenta[d][1,3]dioxol-4-yl]phenyl}ethyl)carbamate **19** (0.36 g, 0.96 mmol, 1.0 eq) and MeOH (4.0 mL), followed by $NaBH_4$ (54.7 mg, 1.45 mmol, 1.5 eq) at 0 °C. The reaction was stirred for 0.5 h then quenched with water (10 mL) and extracted with EtOAc (3x10 mL). The organic layer was dried over anhydrous Na_2SO_4, filtered and the filtrate was concentrated. The residue was dissolved in DCM (2 mL) and was purified by silica gel column chromatography (1:1 Petroleum ether:AcOEt) to afford tert-butyl N-(2-{3-[(3aR,4R,6S,6aS)-6-hydroxy-2,2-dimethyl-tetrahydro-3aH-cyclopenta[d][1,3]dioxol-4-yl]phenyl}ethyl)carba-mate **29** (0.2 g, 56%) as a white solid. LCMS (conditions XBridge C18, 50*3.0 mm, 3.5 µm, 5% - 100% Acetonitrile / 5 mM aqueous NH_4HCO_3 - 2 min, 1.50 mL/min,): T_R = 1.44 min; ES m/z $[M+H]^+$: 378.

(3aS,4S,6R,6aR)-6-(3-(((tert-butyldimethylsilyl)oxy)methyl)phenyl)-2,2-dimethyltetrahydro-4H-cyclopenta[d][1,3]dioxol-4-ol (30)

To a stirred solution of (3aR,6R,6aR)-6-(3-(((tert-butyldimethylsilyl)oxy)methyl)phenyl)-2,2-dimethyltetrahydro-4H-cyclopenta[d][1,3]dioxol-4-one **20** (4.1 g, 10.5 mmol, 1.0 eq) in MeOH (30.0 mL) were added $NaBH_4$ (0.6 g, 15.8 mmol, 1.5 eq) in portions at 0°C under N_2 atmosphere. The resulting mixture was stirred for additional 30 min at room temperature. The reaction was quenched with ice water at 0 °C. The resulting mixture was extracted with AcOEt (3 x 50.0 mL). The combined organic layers were washed with brain, dried over

anhydrous Na_2SO_4. After filtration, the filtrate was concentrated under reduced pressure. The residue was dissolved in DCM (8.0 mL) and purified by silica gel column chromatography (50:50 Petroleum ether/THF) to afford (3aS,4S,6R,6aR)-6-(3-(((tert-butyldimethylsilyl)oxy)methyl)phenyl)-2,2-dimethyltetrahydro-4H-cyclopenta[d][1,3]dioxol-4-ol **30** (3.2g, 77%) as a white solid. LCMS (conditions L-column3 C18, 50*3.0 mm, 3.0 um, Mobile Phase A: Water/5mM NH_4HCO_3, Mobile Phase B: Methanol, 10%-95%B-3 min (+-), 1.50 L/min, ES, m/z): T_R =2.54 min; $[M-H]^-$: 377.

5-bromo-2,4-dichloro-7*H*-pyrrolo[2,3-*d*]pyrimidine (31)

2,4-dichloro-7*H*-pyrrolo[2,3-*d*]pyrimidine (5.00 g, 26.33 mmol) was dissolved in THF (0.20 M, 131.60 mL). Then, NBS (5.21 g, 28.96 mmol) was added in one portion. The resulted mixture was stirred overnight at room temperature protected from light. The reaction mixture was concentrated under reduced pressure and the obtained residue was purified over silica gel chromatography to afford the title compound as beige solid (6.22 g, 88%). R_f = 0.28 (n-heptane/EtOAc; 50%). ^{1}H-NMR (DMSO-d_6, 400 MHz) δ 13.17 (s, 1H, -N*H*), 8.00 (s, 1H, H6). APCI: calc. for $C_6H_3Cl_2BrN_3$ $[M+H]^+$: 265.88, found: 265.8/267.8/269.8 $[M+H]^+$.

4-chloro-7-((3a*S*,4*R*,6*R*,6a*R*)-2,2-dimethyl-6-phenyltetrahydro-4*H*-cyclopenta[*d*][1,3]dioxol-4-yl)-7*H*-pyrrolo[2,3-*d*]pyrimidine (32)

Following the general procedure **D** for esterification & substitution, compound **32** was obtained from compound **22** (0.20 g, 0.85 mmol), with Tf_2O (0.29 mL, 1.69 mmol), dry pyridine (0.21 mL, 2.54 mmol) in CH_2Cl_2 (0.10 M, 8.50 mL) followed by substitution with NaH (60%, 0.06 g, 1.45 mmol), 6-chloro-7-deazapurine (0.14 g, 0.87 mmol) in dry DMF (0.08 M, 9.00 mL) after column chromatography on silica (cyclohexane/EtOAc; 0-60%) as white foam (189 mg, 59%). R_f = 0.59 (cyclohexane/EtOAc; 40%). ^{1}H NMR (400 MHz, DMSO-d_6) δ 8.68 (s, 1H, H2), 8.12 (d, J = 3.7 Hz, 1H, H6), 7.43 – 7.33 (m, 4H, *o*,*m*-Ar), 7.29 – 7.24 (m, 1H, *p*-

Ar), 6.77 (d, *J* = 3.6 Hz, 1H, H5), 5.29 (ddd, *J* = 11.3, 7.6, 5.5 Hz, 1H, H1′), 5.00 (dd, *J* = 7.6, 5.5 Hz, 1H, H2′), 4.78 (t, *J* = 7.2 Hz, 1H, H3′), 3.34 – 3.26 (m, 1H, H4′), 2.64 – 2.54 (m, 2H, H5′), 1.54 (s, 3H, C*H*3, acetonide), 1.23 (s, 3H, C*H*3, acetonide). APCI: calc. for $C_{20}H_{21}ClN_3O_2$ $[M+H]^+$: 370.12, found: 370.1/372.1 $[M+H]^+$.

4-chloro-7-((3a*S*,4*R*,6*R*,6a*R*)-2,2-dimethyl-6-(pyridin-4-yl)tetrahydro-4*H*-cyclopenta[*d*][1,3]dioxol-4-yl)-7*H*-pyrrolo[2,3-*d*]pyrimidine (33)

Following the general procedure **E** for Mitsunobu reaction, compound **33** was obtained starting from compound **23** (0.20 g, 0.84 mmol), 6-chloro-7-deazapurine (0.19 g, 1.18 mmol), PPh_3 (0.45 g, 1.68 mmol), and DIAD (0.30 mL, 1.52 mmol) in dry THF (0.15 M, 5.60 mL) after column chromatography on silica (cyclohexane/EtOAc; 0-100%) as white foam (359 mg, 0.97 mmol). R_f = 0.78 (cyclohexane/EtOAc; 50%). ^1H NMR (400 MHz, DMSO-*d*6) *δ* 8.68 (s, 1H, H2 adenine), 8.56 – 8.52 (m, 2H, H2, H6 pyridine), 8.11 (d, *J* = 3.7 Hz, 1H, H6 adenine), 7.45 – 7.41 (m, 2H, H3, H5 pyridine), 6.78 (d, *J* = 3.7 Hz, 1H, H5), 5.39 – 5.27 (m, 1H, H1′), 4.99 (dd, *J* = 7.5, 5.3 Hz, 1H, H2′), 4.82 (t, *J* = 7.2 Hz, 1H, H3′), 3.39 – 3.34 (m, 1H, H4′), 2.65 – 2.57 (m, 2H, H5′), 1.55 (s, 3H, C*H*3, acetonide), 1.24 (s, 3H, C*H*3, acetonide). APCI: calc. for $C_{19}H_{20}ClN_4O_2$ $[M+H]^+$: 371.12, found: 371.2/373.2 $[M+H]^+$.

4-chloro-7-((3a*S*,4*R*,6*S*,6a*R*)-2,2-dimethyl-6-(thiophen-2-yl)tetrahydro-4*H*-cyclopenta[*d*][1,3]dioxol-4-yl)-7*H*-pyrrolo[2,3-*d*]pyrimidine (34)

Following the general procedure **E** for Mitsunobu reaction, compound **34** was obtained starting from compound **24** (0.25 g, 1.03 mmol), 6-chloro-7-deazapurine (0.34 g, 1.44 mmol), PPh_3 (0.55 g, 2.06 mmol), and DIAD (0.37 mL, 1.85 mmol) in dry THF (0.25 M, 4.10 mL) after column chromatography on silica (cyclohexane/EtOAc; 0-80%) as colorless resin (197 mg, 51%). R_f = 0.73 (cyclohexane/EtOAc; 50%). ^1H NMR (400 MHz, DMSO-*d*6) *δ* 8.68 (s, 1H, H2), 8.08 (d, *J* = 3.7 Hz, 1H, H6), 7.42 (dd, *J* = 4.8, 1.5 Hz, 1H, H5 thiophene), 7.04 – 6.99 (m, 2H, H3, H4 thiophene), 6.75 (d, *J* = 3.6 Hz, 1H, H5), 5.28 (ddd, *J* = 10.7, 8.1, 5.2 Hz, 1H, H1′), 5.00 (dd, *J* = 7.5, 5.2 Hz, 1H,

H2′), 4.77 (t, *J* = 7.2 Hz, 1H, H3′), 3.56 (dt, *J* = 11.3, 7.3 Hz, 1H, H4′), 2.68 – 2.59 (m, 2H, H5′), 1.54 (s, 3H, C*H*3, acetonide), 1.24 (s, 3H, C*H*3, acetonide). APCI: calc. for $C_{18}H_{29}ClN_3O_2S$ [M+H]$^+$: 376.08, found: 376.2/378.2 [M+H]$^+$.

4-chloro-7-((3a*S*,4*R*,6*R*,6a*R*)-2,2-dimethyl-6-(1*H*-pyrazol-5-yl)tetrahydro-4*H*-cyclopenta[*d*][1,3]dioxol-4-yl)-7*H*-pyrrolo[2,3-*d*]pyrimidine (35)

Following the general procedure **D** for Mitsunobu reaction, compound **35** was obtained starting from compound **25** (0.26 g, 0.79 mmol), 6-chloro-7-deazapurine (0.31 g, 1.11 mmol), PPh_3 (0.42 g, 1.59 mmol), and DIAD (0.29 mL, 1.43 mmol) in dry THF (0.15 M, 5.30 mL) after column chromatography on silica (petrol ether/EtOAc; 0-50%) as white foam (267 mg, 0.58 mmol). R_f = 0.42 (petrol ether/EtOAc; 50%). ^{1}H NMR (400 MHz, DMSO-d_6) δ 8.70 (s, 1H, H2), 8.07 (d, *J* = 3.7 Hz, 1H, H6), 7.90 (dd, *J* = 2.3, 0.6 Hz, 1H, H3 pyrazol), 7.56 (dd, *J* = 1.8, 0.5 Hz, 1H, -N*H* pyrazol), 6.77 (d, *J* = 3.7 Hz, 1H, H5), 6.32 (dd, *J* = 2.3, 1.9 Hz, 1H, H4 pyrazol), 5.32 – 5.25 (m, 1H, H1′), 5.10 (dd, *J* = 7.7, 5.6 Hz, 1H, H2′), 5.02 (dd, *J* = 7.7, 5.4 Hz, 1H, H3′), 4.93 – 4.85 (m, 1H, H4′), 3.01 (q, *J* = 12.2 Hz, 1H, H5′), 2.76 – 2.66 (m, 1H, H5′), 1.56 (s, 3H, C*H*3, acetonide), 1.40 (s, 3H, C*H*3, acetonide). APCI: calc. for $C_{17}H_{19}ClN_5O_2$ [M+H]$^+$: 360.11, found: 360.2/362.1 [M+H]$^+$.

4-[(3aR,4R,6R,6aS)-6-{4-chloropyrrolo[2,3-d]pyrimidin-7-yl}-2,2-dimethyl-tetrahydro-3aH-cyclopenta[d][1,3]dioxol-4-yl]-1-(oxan-2-yl)pyrazole (36)

Into an 8 mL vial were added (3aS,4S,6R,6aR)-2,2-dimethyl-6-[1-(oxan-2-yl)pyrazol-4-yl]-tetrahydro-3aH-cyclopenta[d][1,3]dioxol-4-ol (0.2 g, 0.65 mmol, 1.2 eq), 4-chloro-7H-pyrrolo[2,3-d]pyrimidine (83.3 mg, 0.54 mmol, 1.0 eq) and DIAD (0.22 g, 1.08 mmol, 2.0 eq), followed by PPh_3 (0.28 g, 1.08 mmol, 2.0 eq) in THF (5 mL) dropwise at 0 °C. The reaction was stirred for 12 h at room temperature, then concentrated. The residue was dissolved in DCM (3 mL) and was purified by silica gel column chromatography (1:1 Petroleum ether:AcOEt) to afford 4-[(3aR,4R,6R,6aS)-6-{4-chloropyrrolo[2,3-d]pyrimidin-7-yl}-2,2-dimethyl-tetrahydro-3aH-

cyclopenta[d][1,3]dioxol-4-yl]-1-(oxan-2-yl)pyrazole **36** (0.19 g, 80%) as a white solid. LCMS (conditions XBridge C18, 50*3.0 mm, 3.5 μm, Mobile Phase A: Water/5mM NH_4HCO_3, Mobile Phase B: Acetonitrile, 5% - 100% B - 2 min, 1.50 mL/min,): T_R = 1.47 min; ES m/z $[M+H]^+$: 444, 446.

***tert*-butyl (3-((3aR,4R,6R,6aS)-6-(4-chloro-7H-pyrrolo[2,3-d]pyrimidin-7-yl)-2,2-dimethyltetrahydro-4H-cyclopenta[d][1,3]dioxol-4-yl)benzyl)carbamate (37)**

To a stirred solution of tert-butyl (3-((3aR,4R,6S,6aS)-6-hydroxy-2,2-dimethyltetrahydro-4H-cyclopenta[d][1,3]dioxol-4-yl)benzyl)carbamate (0.31 g, 0.8 mmol, 1.2 eq), 4-chloro-7H-pyrrolo[2,3-d]pyrimidine (0.11 g, 0.7 mmol, 1.0 eq) and PPh_3 (0.37 g, 1.4 mmol, 2.0 eq) in toluene (2.5 mL) was added DBAD (0.33 g, 1.4 mmol, 2.0 eq) in toluene (2.5 mL) dropwise at 0°C. The reaction mixture was stirred at 50°C for 2 h, then concentrated under reduced pressure. The residue was dissolved in DCM (3.0 mL) and purified by silica gel column chromatography (60:40 Petroleum ether/THF) to afford tert-butyl (3-((3aR,4R,6R,6aS)-6-(4-chloro-7H-pyrrolo[2,3-d]pyrimidin-7-yl)-2,2-dimethyltetrahydro-4H-cyclopenta[d][1,3]dioxol-4-yl)benzyl)carbamate **37** (220 mg, 61%) as a white solid. LCMS (conditions: L-column3 ODS, 50*3.0 mm, 3.0 μm, Mobile Phase A: Water/5mM NH_4HCO_3+0.05% Ammonia, Mobile Phase B: Acetonitrile, 5%-100% B - 3 min, 1.50 L/min): T_R = 2.04 min; ES m/z $[M+H]^+$: 499, 501.

***tert*-butyl N-(2-{3-[(3aR,4R,6R,6aS)-6-{4-chloropyrrolo[2,3-d]pyrimidin-7-yl}-2,2-dimethyl-tetrahydro-3aH-cyclopenta[d][1,3]dioxol-4-yl]phenyl}ethyl)carbamate (38)**

Into an 8 mL vial were added tert-butyl N-(2-{3-[(3aR,4R,6S,6aS)-6-hydroxy-2,2-dimethyl-tetrahydro-3aH-cyclopenta[d][1,3]dioxol-4-yl]phenyl}ethyl)carbamate (0.2 g, 0.54 mmol, 1.3 eq), 4-chloro-7H-pyrrolo[2,3-d]pyrimidine (63.2 mg, 0.41 mmol, 1.0 eq) and PPh_3 (0.22 g, 0.82 mmol, 2.0 eq) in THF (3 mL), followed by DIAD (0.17 g, 0.82 mmol, 2.0 eq) at 0 °C. The resulting mixture was stirred for 12h, then concentrated.

The residue was dissolved in DCM (2 mL) and was purified by silica gel column chromatography (1:1 Petroleum ether:AcOEt) to afford tert-butyl N-(2-{3-[(3aR,4R,6R,6aS)-6-{4-chloropyrrolo[2,3-d]pyrimidin-7-yl}-2,2-dimethyl-tetrahydro-3aH-cyclopenta[d][1,3]dioxol-4-yl]phenyl}ethyl)carbamate **38** (0.19 g, 88%) as a white solid. LCMS (conditions XBridge C18, 50*3.0 mm, 3.5 μm, 5% - 100% Acetonitrile / 5 mM aqueous NH_4HCO_3 - 2 min, 1.50 mL/min,): T_R = 1.64 min; ES m/z $[M+H]^+$: 513, 515.

***tert*-butyl N-({3-[(3aR,4R,6R,6aS)-6-{2,4-dichloropyrrolo[2,3-d]pyrimidin-7-yl}-2,2-dimethyl-tetrahydro-3aH-cyclopenta[d][1,3]dioxol-4-yl]phenyl}methyl)carbamate (39)**

Into a 40 mL vial was placed a solution of tert-butyl N-({3-[(3aR,4R,6S,6aS)-6-hydroxy-2,2-dimethyl-tetrahydro-3aH-cyclopenta[d][1,3]dioxol-4-yl]phenyl}methyl)carbamate (0.41 g, 1.1 mmol, 1.4 eq) in THF (2.0 mL). 2,4-Dichloro-7H-pyrrolo[2,3-d]pyrimidine (0.15 g, 0.8 mmol, 1.0 eq) and PPh_3 (0.42 g, 1.6 mmol, 2.0 eq) were added, followed by DIAD (0.32 g, 1.6 mmol, 2.0 eq) dropwise over 2 min at 0°C. The resulting mixture was stirred for 10 h at 40°C, then concentrated under reduced pressure. The residue was dissolved in DCM (2.0 ml) and purified by silica gel column chromatography (70:30 Petroleum ether:THF) to afford tert-butyl N-({3-[(3aR,4R,6R,6aS)-6-{2,4-dichloropyrrolo[2,3-d]pyrimidin-7-yl}-2,2-dimethyl-tetrahydro-3aH-cyclopenta[d][1,3]dioxol-4-yl]phenyl}methyl)carbamate **39** (280 mg, 65%) as a colorless oil. LCMS (conditions: Kinetex EVO C18, 50*3.0 mm, 2.6um, Mobile Phase A: Water/5mM NH_4HCO_3+0.05% Ammonia, Mobile Phase B: Acetonitrile/5%water, 2%-100% B - 3 min, 1.50 L/min): T_R = 2.06 min; ES m/z $[M+H]^+$: 533, 535.

7-((3aS,4R,6R,6aR)-6-(3-(((tert-butyldimethylsilyl)oxy)methyl)phenyl)-2,2-dimethyltetrahydro-4H-cyclopenta[d][1,3]dioxol-4-yl)-2,4-dichloro-7H-pyrrolo[2,3-d]pyrimidine (40)

To a stirred solution of 2,4-dichloro-7H-pyrrolo[2,3-d]pyrimidine (0.36 g, 1.8 mmol, 1.0 eq), (3aS,4S,6R,6aR)-6-(3-(((tert-butyldimethylsilyl)oxy)methyl)phenyl)-2,2-dimethyltetrahydro-4H-cyclopenta[d][1,3]dioxol-4-ol (1.08 g, 2.8 mmol, 1.5 eq) and PPh_3 (1.0 g, 3.8 mmol, 2.0 eq) in toluene (4.0 mL) were added DBAD (0.88 g, 3.8 mmol, 2.0 eq) which dissolved in toluene (3.0 mL) dropwise at 0°C under N_2 atmosphere. The resulting mixture was stirred for additional 3h at 50 °C. The resulting mixture was concentrated under reduced pressure. The residue was dissolved in DCM (5.0 mL) and purified by silica gel column chromatography (50:50 Petroleum ether/THF) to afford 7-((3aS,4R,6R,6aR)-6-(3-(((tert-butyldimethylsilyl)oxy)methyl)phenyl)-2,2-dimethyltetrahydro-4H-cyclopenta[d][1,3]dioxol-4-yl)-2,4-dichloro-7H-pyrrolo[2,3-d]pyrimidine **40** (460 mg, 43%) as a white solid. LCMS (conditions Cortecs C18+, 50*3.0 mm, 2.7 μm, Mobile Phase A: Water/0.05%TFA, Mobile Phase B: Acetonitrile, 2%-100%B-3min, ES, m/z): T_R =2.60 min; $[M+H]^+$: 548, 550.

5-bromo-7-((3aS,4R,6R,6aR)-6-(3-(((tert-butyldimethylsilyl)oxy)methyl)phenyl)-2,2-dimethyltetrahydro-4H-cyclopenta[d][1,3]dioxol-4-yl)-2,4-dichloro-7H-pyrrolo[2,3-d]pyrimidine (41)

A solution of 5-bromo-2,4-dichloro-7H-pyrrolo[2,3-d]pyrimidine (1.7 g, 6.51 mmol, 1.0 equiv), (3aS,4S,6R,6aR)-6-(3-{[(tert-butyldimethylsilyl)oxy]methyl}phenyl)-2,2-dimethyl-tetrahydro-3aH-cyclopenta[d][1,3]dioxol-4-ol (3.2 g, 8.47 mmol, 1.3 equiv) and PPh_3 (3.4 g, 13.02 mmol, 2.0 equiv) in toluene (20.0 mL) was stirred for 5 min at room temperature under nitrogen atmosphere. To the above mixture was added DBAD (3.0 g, 13.02 mmol, 2.0 equiv) in toluene (20.0 mL) dropwise over 5 min at 0°C. The resulting mixture was stirred for 3 h at 50°C. The resulting mixture was concentrated under reduced pressure. The residue was purified by silica gel column chromatography (4:1 Petroleum ether/THF) to afford 7-[(3aS,4R,6R,6aR)-6-(3-{[(tert-

butyldimethylsilyl)oxy]methyl}phenyl)-2,2-dimethyl-tetrahydro-3aH-cyclopenta[d][1,3]dioxol-4-yl]-5-bromo-2,4-dichloropyrrolo[2,3-d]pyrimidine **41** (3.0 g, 57%) as a white solid. LCMS (conditions L-column3 ODS, 50*3.0 mm, 3.0 um, Mobile Phase A: Water/5mM NH_4HCO_3+0.05% Ammonia, Mobile Phase B: Acetonitrile/5%water, 5%-100%B-3min, ES, m/z):T_R=2.90 min; $[M+H]^+$: 626, 628.

4-iodo-7*H*-pyrrolo[2,3-*d*]pyrimidine (42)

A mixture of 4-chloro-7*H*-pyrrolo[2,3-*d*]pyrimidine (3.00 g, 19.34 mmol) and hydroiodic acid (57%, 4.34 g, 19.34 mmol) was stirred at room temperature overnight. The resulted solid was filtered off, and suspended with water. The suspension was cooled down to 0 °C and the pH was subsequently adjusted to 8 by adding aqueous ammonia solution. The solid was filtered off, washed with cold water and dried to complete dryness to afford the title product as beige solid (4.19 g, 88%). ^{1}H NMR (400 MHz, DMSO) δ 12.51 (s, 1H, -N*H*), 8.43 (s, 1H, H2), 7.66 (dd, J = 3.6, 2.3 Hz, 1H, H6), 6.32 (dd, J = 3.5, 1.8 Hz, 1H, H5). APCI: calc. for $C_6H_5IN_3$ $[M+H]^+$: 245.94, found: 245.6 $[M+H]^+$.

7*H*-pyrrolo[2,3-*d*]pyrimidine (43)

Compound **42** (2.00 g, 8.08 mmol) was dissolved in MeOH (40.40 mL) under nitrogen atmosphere. Then, Et_3N (1.71 mL, 12.12 mmol) was added, and the resulted solution was degassed. Palladium on activated charcoal moistened with water (1.72 g, 0.81 mmol) was added and the mixture was degassed. Afterwards, the reaction mixture was purged with hydrogen (balloon) and stirred at ambient temperature overnight. After 17 h, the reaction mixture was purged with nitrogen and filtered over celite. The filtrate was concentrated under reduced pressure. The obtained residue was purified over silica (cyclohexane/EtOAc; 0-100%) to afford the pure product (853 mg, 89%). ^{1}H NMR (400 MHz, DMSO-d_6) δ 12.12 (s, 1H- N*H*), 8.99 (s, 1H, H2), 8.74 (s, 1H, H4), 7.56 (dd, J = 3.5, 2.4 Hz, 1H, H6), 6.58 (dd, J = 3.5, 1.8 Hz, 1H, H7). APCI: calc. for $C_6H_6N_3$ $[M+H]^+$: 120.05, found: 119.9 $[M+H]^+$.

5-iodo-7*H*-pyrrolo[2,3-*d*]pyrimidine (44)

Compound **43** (0.84 g, 7.00 mmol) and NIS (1.67 g, 7.35 mmol) was stirred in dry MeCN (11.70 mL) under nitrogen atmosphere overnight. The solution was concentrated to complete dryness and the obtained crude product was purified by flash chromatography (cyclohexane/EtOAc; 0-100%) to afford the title product (1.44 g, 84%). ^{1}H NMR (400 MHz, DMSO-d_6) δ 12.58 (s, 1H, -N$\underline{H}$), 8.81 (s, 1H, H2), 8.74 (s, 1H, H4), 7.83 (d, J = 2.0 Hz, 1H, H6). APCI: calc. for $C_6H_5IN_3$ [M+H]$^+$: 245.94, found: 245.6 [M+H]$^+$.

7-((3a*S*,4*R*,6*R*,6a*R*)-6-(3-(((*tert*-butyldimethylsilyl)oxy)methyl)phenyl)-2,2-dimethyltetrahydro-4*H*-cyclopenta[*d*][1,3]dioxol-4-yl)-5-iodo-7*H*-pyrrolo[2,3-*d*]pyrimidine (45)

Into a 40 mL vial were added 5-iodo-7H-pyrrolo[2,3-d] pyrimidine (250 mg, 2.0 mmol, 1.0 eq), (3aS,4S,6R,6aR)-6-(3-{[(tert-butyldimethylsilyl)oxy]methyl}phenyl)-2,2-dimethyl-tetrahydro-3aH-cyclopenta[d][1,3]dioxol-4-ol (500 mg, 2.6 mmol, 1.3 eq), PPh_3 (503 mg, 4.0 mmol, 2.0 eq) and THF (3.0 mL) at room temperature. To the above mixture was added DIAD (415 mg, 4.0 mmol, 2.0 eq) in THF (2.0 mL) dropwise over 10 min at 0°C. The resulting mixture was stirred for 6 h at room temperature and was concentrated under reduced pressure. The residue was dissolved in DCM (2.0 mL) and was purified by silica gel column chromatography (5:1 Petroleum ether: Tetrahydrofuran) to afford 7-[(3aS,4R,6R,6aR)-6-(3-{[(tert-butyldimethylsilyl)oxy]methyl}phenyl)-2,2-dimethyl-tetrahydro-3aH-cyclopenta[d][1,3]dioxol-4-yl]-5-iodopyrrolo[2,3-d]pyrimidine **45** (450 mg, 75%) as a white solid. LCMS (conditions Halo C18, 30*3.0 mm, 2.0 μm, Mobile Phase A: Water/0.05%TFA, Mobile Phase B: Acetonitrile, 5%-95%B-1.2 min, 1.50 L/min, ES, m/z): T_R = 0.98 min; [M+H]$^+$: 606.

7-((3a*S*,4*R*,6*R*,6a*R*)-2,2-dimethyl-6-phenyltetrahydro-4*H*-cyclopenta[*d*][1,3]dioxol-4-yl)-7*H*-pyrrolo[2,3-*d*]pyrimidin-4-amine (46)

Following the general procedure **F** for aromatic substitution, compound **46** was obtained starting from compound **32** (0.18 g, 0.45 mmol) in a mixture of 1,4-dioxane and ammonia (0.05 M, 9.60 mL, 1:2) after column chromatography on silica (cyclohexane/EtOAc; 0-60%) as white foam (72 mg, 89%). R_f = 0.52 (cyclohexane/EtOAc; 60%).^{1}H NMR (400 MHz, DMSO-d_6) δ 8.07 (s, 1H, H2), 7.44 (d, J = 3.6 Hz, 1H, H6), 7.41 – 7.32 (m, 4H, *o*,*m*-Ar), 7.29 – 7.22 (m, 1H, *p*-Ar), 6.98 (s, 2H, -CN$\underline{H}_2$), 6.59 (d, J = 3.6 Hz, 1H, H5), 5.12 (ddd, J = 10.8, 8.1, 5.4 Hz, 1H, H1′), 4.96 (dd, J = 7.6, 5.4 Hz, 1H, H2′), 4.75 (t, J = 7.2 Hz, 1H, H3′), 3.29 – 3.22 (m, 1H, H4′), 2.54 – 2.45 (m, 2H, H5′ under DMSO peak), 1.52 (s, 3H, C$\underline{H}_3$, acetonide), 1.22 (s, 3H, C$\underline{H}_3$, acetonide). APCI: calc. for $C_{20}H_{23}N_4O_2$ [M+H]$^+$: 351.17, found: 351.3 [M+H]$^+$.

7-((3a*S*,4*R*,6*R*,6a*R*)-2,2-dimethyl-6-(pyridin-4-yl)tetrahydro-4*H*-cyclopenta[*d*][1,3]dioxol-4-yl)-7*H*-pyrrolo[2,3-*d*]pyrimidin-4-amine (47)

Following the general procedure **F** for aromatic substitution, compound **47** was obtained starting from compound **33** (0.35 g, 0.93 mmol) in a mixture of 1,4-dioxane and ammonia (0.10 M, 9.40 mL, 1:2) after column chromatography on silica (CH_2Cl_2/MeOH; 0-10%) as white foam (66.20 mg, 20%). R_f = 0.59 (CH_2Cl_2/MeOH; 10%). ^{1}H NMR (400 MHz, DMSO-d_6) δ 8.55 – 8.51 (m, 2H, H2, H6 pyridine), 8.07 (s, 1H, H2 adenine), 7.44 (d, J = 3.6 Hz, 1H, H6 adenine), 7.42 – 7.39 (m, 2H, H3, H5 pyridine), 7.00 (s, 2H, -CN$\underline{H}_2$), 6.60 (d, J = 3.5 Hz, 1H, H5 adenine), 5.14 (ddd, J = 11.0, 7.8, 5.2 Hz, 1H, H1′), 4.95 (dd, J = 7.5, 5.2 Hz, 1H, H2′), 4.79 (t, J = 7.2 Hz, 1H, H3′), 3.31 – 3.24 (m, 1H, H4′), 2.52 (s, 2H, H5′ under DMSO peak), 1.53 (s, 3H, C$\underline{H}_3$, acetonide), 1.23 (s, 3H, C$\underline{H}_3$, acetonide). APCI calc. for $C_{19}H_{22}N_5O_2$ [M+H]$^+$: 352.17, found: 311.3 [M+H]$^+$.

7-((3a*S*,4*R*,6*S*,6a*R*)-2,2-dimethyl-6-(thiophen-2-yl)tetrahydro-4*H*-cyclopenta[*d*][1,3]dioxol-4-yl)-7*H*-pyrrolo[2,3-*d*]pyrimidin-4-amine (48)

Following the general procedure **F** for aromatic substitution, compound **48** was obtained starting from compound **34** (0.20 g, 0.52 mmol) in a mixture of 1,4-dioxane and ammonia (0.10 M, 5.20 mL, 1:2) after column chromatography on silica (cyclohexane/EtOAc; 0-100%) as white foam (27 mg, 15%). R_f = 0.67 (CH_2Cl_2/MeOH; 10%). ^{1}H NMR (400 MHz, DMSO-d_6) δ 8.06 (s, 1H, H2), 7.42 – 7.38 (m, 2H, H6, H5 thiophene), 7.03 – 6.96 (m, 4H, -CN$\underline{H}_2$, H3, H4 thiophene), 6.58 (d, *J* = 3.5 Hz, 1H, H5), 5.15 – 5.04 (m, 1H, H1′), 4.96 (dd, *J* = 7.5, 5.2 Hz, 1H, H2′), 4.73 (t, *J* = 7.2 Hz, 1H, H3′), 3.49 (dt, *J* = 11.3, 7.3 Hz, 1H, H4′), 2.60 – 2.52 (m, 2H, H5′), 1.52 (s, 3H, C$\underline{H}_3$, acetonide), 1.23 (s, 3H, C$\underline{H}_3$, acetonide). APCI calc. for $C_{18}H_{21}N_4O_2S$ $[M+H]^+$: 357.13, found: 357.1 $[M+H]^+$.

7-((3a*S*,4*R*,6*R*,6a*R*)-2,2-dimethyl-6-(1*H*-pyrazol-5-yl)tetrahydro-4*H*-cyclopenta[*d*][1,3]dioxol-4-yl)-7*H*-pyrrolo[2,3-*d*]pyrimidin-4-amine (49)

Following the general procedure **F** for aromatic substitution, compound **49** was obtained starting from compound **35** (0.26 g, 0.50 mmol) in a mixture of 1,4-dioxane and ammonia (0.10 M, 5.00 mL, 1:2) after column chromatography on silica (CH_2Cl_2, MeOH; 0-20%) as white foam (27 mg, 0.08 mmol). R_f = 0.43 (CH_2Cl_2, MeOH; 10%). APCI calc. for $C_{17}H_{21}N_6O_2$ $[M+H]^+$: 341.16; found: 340.9 $[M+H]^+$.

7-[(3aS,4R,6R,6aR)-2,2-Dimethyl-6-[1-(oxan-2-yl)pyrazol-4-yl]-tetrahydro-3aH-cyclopenta[d][1,3]dioxol-4-yl]-N-[(4-methoxyphenyl)methyl]pyrrolo[2,3-d]pyrimidin-4-amine (50)

Into an 8 mL vial were added 4-[(3aR,4R,6R,6aS)-6-{4-chloropyrrolo[2,3-d]pyrimidin-7-yl}-2,2-dimethyl-tetrahydro-3aH-cyclopenta[d][1,3]dioxol-4-yl]-1-(oxan-2-yl)pyrazole **36** (96 mg, 0.22 mmol, 1.0 eq), (4-methoxyphenyl)methanamine (89 mg, 0.65 mmol, 3.0 eq) and TEA (43.8 mg, 0.43 mmol, 2.0 eq) in EtOH (2 mL) at room temperature. The reaction was stirred for 1 h at 80 °C. The resulting mixture was concentrated to afford 7-[(3aS,4R,6R,6aR)-2,2-dimethyl-6-[1-(oxan-2-yl)pyrazol-4-

yl]-tetrahydro-3aH-cyclopenta[d][1,3]dioxol-4-yl]-N-[(4-methoxyphenyl)methyl]pyrrolo[2,3-d]pyrimidin-4-amine **50** (80 mg, 68%) as a yellow solid. LCMS (conditions Xbridge Shield C18, 50*3.0 mm, 3.5 µm, Mobile Phase A: Water/0.05% Ammonia, Mobile Phase B: Acetonitrile, 5% - 100% B - 3 min, 1.50 mL/min): T_R = 1.90 min; ES m/z $[M+H]^+$: 545.

***tert*-butyl (3-((3aR,4R,6R,6aS)-6-(4-amino-7*H*-pyrrolo[2,3-d]pyrimidin-7-yl)-2,2-dimethyltetrahydro-4*H*-cyclopenta[d][1,3]dioxol-4-yl)phenyl)carbamate (51)**

950 mg (2.72 mmol; 1.00 eq) of *tert*-butyl (3-((3aR,4R,6aS)-6-hydroxy-2,2-dimethyltetrahydro-4*H*-cyclopenta[d][1,3]dioxol-4-yl)phenyl)carbamate were dissolved in 30 mL of Toluene under inert gas atmosphere. At 0 °C, 1427 mg (5.44 mmol; 2.00 eq) of PPh_3 were added. 10 minutes later 1253 mg (5.44 mmol; 2.00 eq) of Di-*tert*-butyl-azodicarboxylate were attached. The reaction solution was stirred for further 10 minutes before 544 mg (3.54 mmol; 1.30 eq) of 4-chloro-7*H*-pyrrolo[2,3-d]pyrimidine were added and the whole was heated to 80 °C overnight. The solvent was removed under reduced pressure and the crude was purified by column chromatography (Cyclohexane/EtOAc 99:1% to 60:40%). The product was directly used without further characterization. Therefore 20 mL of Dioxane and 20 mL of a 25% aqueous solution of NH_3 were added. The reaction solution was heated to 100 °C in a sealed tube overnight. The solvent was then removed under reduced pressure and the resulting residue was adsorbed on silica and purified by flash chromatography (DCM/MeOH 99%/1% to 90%/10%). 170 mg (0.36 mmol; 13% over 2 steps) of the title compound were obtained in the form of a colorless foam. R_f = 0.41 (DCM/MeOH 10:1). ^{1}H-NMR (400 MHz, DMSO-d_6) δ = 9.30 (s, 1H), 8.08 (s, 1H), 7.48 (s, 1H), 7.43 (d, *J* = 3.6 Hz, 1H), 7.33-7.26 (m, 1H), 7.24-7.20 (m, 1H), 7.03-6.98 (m, 1H), 6.60 (d, *J* = 3.5 Hz, 1H), 5.12-5.06 (m, 1H), 4.99-4.96 (m, 1H), 4.72 (t, *J* = 7.2 Hz, 1H), 3.24-3.14 (m, 2H), 2.46-2.41 (m, 1H), 1.52 (s, 3H), 1.47 (s, 9H), 1.22 (s, 3H) ppm. APCI-MS(+) m/z for $C_{25}H_{29}ClN_4O_4$: calc.: 484.98; found: 484.6 and 485.5. APCI-MS(+) m/z for $C_{25}H_{31}N_5O_4$: calc.: 465.55; found: 465.7 and 466.7.

***tert*-butyl(3-((3aR,4R,6R,6aS)-6-(4-((4-methoxybenzyl)amino)-7H-pyrrolo[2,3-d]pyrimidin-7-yl)-2,2-dimethyltetrahydro-4H-cyclopenta[d][1,3]dioxol-4-yl)benzyl)carbamate (52)**

Into a 40 mL vial were added tert-butyl (3-((3aR,4R,6R,6aS)-6-(4-chloro-7H-pyrrolo[2,3-d]pyrimidin-7-yl)-2,2-dimethyltetrahydro-4H-cyclopenta[d][1,3]dioxol-4-yl)benzyl)carbamate (0.21 g, 0.4 mmol, 1.0 eq), 4-methoxy-benzenemethanamine, (0.23 g, 1.7 mmol, 4.0 eq), TEA (0.13 g, 1.3 mmol, 3.0 eq) and EtOH (3.0 mL). The reaction mixture was stirred at 80 °C for 2 h, then allowed to cool to room temperature. The resulting mixture was concentrated under reduced pressure. The residue was dissolved in DCM (5.0 mL) and purified by silica gel column chromatography (1:1 Petroleum ether/THF) to afford tert-butyl (3-((3aR,4R,6R,6aS)-6-(4-((4-methoxybenzyl)amino)-7H-pyrrolo[2,3-d]pyrimidin-7-yl)-2,2-dimethyltetrahydro-4H-cyclopenta[d][1,3]dioxol-4-yl)benzyl)carbamate **52** (150 mg, 59%) as a white solid. LCMS (conditions: L-column3 ODS, 50*3.0 mm, 3.0 μm, Mobile Phase A: Water/5mM NH_4HCO_3+0.05% Ammonia, Mobile Phase B: Acetonitrile, 5%-100%B - 2.5 min, 1.50 L/min): T_R = 1.88 min; ES m/z $[M+H]^+$: 600.

***tert*-butyl N-(2-{3-[(3aR,4R,6R,6aS)-6-(4-{[(4-methoxyphenyl)methyl]amino}pyro-lo[2,3-d]pyrimidin-7-yl)-2,2-dimethyl-tetrahydro-3aH-cyclopenta[d][1,3]dioxol-4-yl]phenyl}ethyl)carbamate (53)**

Into an 8 mL vial were added tert-butyl N-(2-{3-[(3aR,4R,6R,6aS)-6-{4-chloropyrrolo[2,3-d]pyrimidin-7-yl}-2,2-dimethyl-tetrahydro-3aH-cyclopenta[d][1,3]dioxol-4-yl]phenyl}ethyl)carbamate (0.19 g, 0.36 mmol, 1.0 eq), (4-methoxyphenyl)methanamine (0.15 g, 1.1 mmol, 3.0 eq) and TEA (73.1 mg, 0.73 mmol, 2.0 eq) in EtOH (4.0 mL). The reaction was stirred for 2 h at 80 °C, then concentrated. The residue was dissolved in DCM (2 mL) and was purified by silica gel column chromatography (1:1 Petroleum ether:AcOEt) to afford tert-butyl N-(2-{3-[(3aR,4R,6R,6aS)-6-(4-{[(4-methoxyphenyl)methyl]amino}pyro-lo[2,3-d]pyrimidin-7-yl)-2,2-dimethyl-tetrahydro-3aH-cyclopenta[d][1,3]dioxol-4-yl]phen-yl}ethyl)carbamate **53** (99 mg, 44%) as a white solid. LCMS (conditions XBridge C18,

50*3.0 mm, 3.5 μm, Mobile Phase A: Water/5mM NH_4HCO_3, Mobile Phase B: Methanol, 5% - 100% B - 2 min, 1.50 mL/min,): T_R = 1.55 min; ES m/z $[M+H]^+$: 614.

***tert*-butyl N-({3-[(3aR,4R,6R,6aS)-6-{4-amino-2-chloropyrrolo[2,3-d]pyrimidin-7-yl}-2,2-dimethyl-tetrahydro-3aH-cyclopenta[d][1,3]dioxol-4-yl]phenyl}methyl)carbamate (54)**

Into a 40 mL vial was placed a solution of tert-butyl N-({3-[(3aR,4R,6R,6aS)-6-{2,4-dichloropyrrolo[2,3-d]pyrimidin-7-yl}-2,2-dimethyl-tetrahydro-3aH-cyclopenta[d][1,3]dioxol-4-yl]phenyl}methyl)carbamate (0.22 g, 0.4 mmol, 1.0 eq) in 7 M NH_3 in MeOH (2.0 mL). The mixture was stirred for 8h at 80°C. The resulting mixture was concentrated under vacuum to afford tert-butyl N-({3-[(3aR,4R,6R,6aS)-6-{4-amino-2-chloropyrrolo[2,3-d]pyrimidin-7-yl}-2,2-dimethyl-tetrahydro-3aH-cyclopenta[d][1,3]dioxol-4-yl]phenyl}methyl)carbamate **54** (180 mg, crude) as a yellow solid. LCMS (Conditions: L-column3 ODS, 50*3.0 mm, 3.0 μm, Mobile PhaseA: Water/5mM NH_4HCO_3+0.05% Ammonia, Mobile Phase B: Acetonitrile/5%water, 2%-100% B - 3 min, 1.50 L/min): T_R = 2.01 min; ES m/z $[M+H]^+$: 514, 516.

7-((3aS,4R,6R,6aR)-6-(3-(((tert-butyldimethylsilyl)oxy)methyl)phenyl)-2,2-dimethyltetrahydro-4H-cyclopenta[d][1,3]dioxol-4-yl)-2-chloro-N-(4-methoxybenzyl)-7H-pyrrolo[2,3-d]pyrimidin-4-amine (55)

Into a 40 mL vial were added 7-((3aS,4R,6R,6aR)-6-(3-(((tert-butyldimethylsilyl)oxy)methyl)phenyl)-2,2-dimethyltetrahydro-4H-cyclopenta[d][1,3]dioxol-4-yl)-2,4-dichloro-7H-pyrrolo[2,3-d]pyrimidine (0.44 g, 0.8 mmol, 1.0 eq), TEA (162 mg, 1.6 mmol, 2.0 eq) and (4-methoxyphenyl)methanamine (0.32 g, 2.4 mmol, 3.0 eq) in EtOH (4.0 mL) at room temperature. The resulting mixture was stirred for additional 2 h at 80 °C. The resulting mixture was concentrated under reduced pressure. The residue was dissolved in DCM (3.0 mL) and purified by silica gel column chromatography (50:50 Petroleum ether/THF) to afford 7-

((3aS,4R,6R,6aR)-6-(3-(((tert-butyldimethylsilyl)oxy)methyl)phenyl)-2,2-dimethyltetrahydro-4H-cyclopenta[d][1,3]dioxol-4-yl)-2-chloro-N-(4-methoxybenzyl)-7H-pyrrolo[2,3-d]pyrimidin-4-amine **55** (340 mg, 65%) as a white solid. LCMS (conditions Cortecs C18+, 50*3.0 mm, 2.7 µm, Mobile Phase A: Water/0.05%TFA, Mobile Phase B: Acetonitrile, 2%-100%B-3 min, ES, m/z): T_R =2.30 min; $[M+H]^+$: 649, 651.

5-bromo-7-((3aS,4R,6R,6aR)-6-(3-(((tert-butyldimethylsilyl)oxy)methyl)phenyl)-2,2-dimethyltetrahydro-4H-cyclopenta[d][1,3]dioxol-4-yl)-2-chloro-7H-pyrrolo[2,3-d]pyrimidin-4-amine (56)

Into a 100 mL vial were added 7-[(3aS,4R,6R,6aR)-6-(3-{[(tert-butyldimethylsilyl)oxy]methyl}phenyl)-2,2-dimethyl-tetrahydro-3aH-cyclopenta[d][1,3]dioxol-4-yl]-5-bromo-2,4-dichloropyrrolo[2,3-d]pyrimidine (3.0 g, 4.8 mmol, 1.0 equiv) and 7M NH_3 in MeOH (30.0 mL) at room temperature. The resulting mixture was stirred for 2h at 80°C. The resulting mixture was concentrated under reduced pressure. The residue was purified by silica gel column chromatography (1:1 Petroleum ether/THF) to afford 7-[(3aS,4R,6R,6aR)-6-(3-{[(tert-butyldimethylsilyl)oxy]methyl}phenyl)-2,2-dimethyl-tetrahydro-3aH-cyclopenta[d][1,3]dioxol-4-yl]-5-bromo-2-chloropyrrolo[2,3-d]pyrimidin-4-amine **56** (2.7 g, 92%) as a white solid. LCMS (conditions L-column3 ODS, 50*3.0 mm, 3.0 um, Mobile Phase A: Water (0.05% ammonia water), Mobile Phase B: Acetonitrile, 10%-95%B-3min, ES, m/z,):T_R=2.77 min; $[M+H]^+$: 607, 609.

(1-benzyl-4-chloro-1H-pyrazol-3-yl)boronic acid (57)

Into a 40mL vial were added 1-benzylpyrazol-3-ylboronic acid (300.0 mg, 1.5 mmol, 1.0 equiv), NCS (237.9 mg, 1.8 mmol, 1.2 eq), and THF (6.0 mL) at room temperature. The resulting mixture was stirred for 1 h at 70°C and was monitored by LCMS. After completion of reaction, the resulting mixture was concentrated under vacuum. The crude product (1-benzyl-4-chloro-1H-pyrazol-3-yl)boronic acid **57** (510 mg, 68%

purity) was used in the next step directly without further purification. LCMS (conditions L-column3 ODS, 50*3.0 mm, 3.0 um, Mobile Phase A: Water (0.05% ammonia water), Mobile Phase B: Acetonitrile, 5%-100%B-2min, 1.2 mL/min, ES, m/z,): T_R = 0.89 min; $[M+H]^+$: 237, 239.

5-(1-benzyl-4-chloro-1H-pyrazol-3-yl)-7-((3aS,4R,6R,6aR)-6-(3-(((tert-butyldimethylsilyl)oxy)methyl)phenyl)-2,2-dimethyltetrahydro-4H-cyclopenta[d][1,3]dioxol-4-yl)-2-chloro-7H-pyrrolo[2,3-d]pyrimidin-4-amine (58)

Into a 40 mL vial were added mixture of 7-[(3aS,4R,6R,6aR)-6-(3-{[(tert-butyldimethylsilyl)oxy]methyl}phenyl)-2,2-dimethyl-tetrahydro-3aH-cyclopenta[d][1,3]dioxol-4-yl]-5-bromo-2-chloropyrrolo[2,3-d]pyrimidin-4-amine (150.0 mg, 0.25 mmol, 1.0 eq), 1-benzyl-4-chloropyrazol-3-ylboronic acid (175.0 mg, 0.7 mmol, 3.0 eq) in dioxane (1.5 mL), H_2O (0.15 mL), K_3PO_4 (104.73 mg, 0.494 mmol, 2.0 eq) and Pd(dtbpf)Cl_2 (16.0 mg, 0.02 mmol, 0.1 eq) at room temperature. The resulting mixture was stirred for 1 h at 80°C under nitrogen atmosphere. After completion of reaction, the resulting mixture was concentrated under reduced pressure. The residue was purified by silica gel column chromatography (2:1 petroleum ether/tetrahydrofuran) to afford 7-[(3aS,4R,6R,6aR)-6-(3-{[(tert-butyldimethylsilyl)oxy]methyl}phenyl)-2,2-dimethyl-tetrahydro-3aH-cyclopenta[d][1,3]dioxol-4-yl]-5-(1-benzyl-4-chloropyrazol-3-yl)-2-chloropyrrolo[2,3-d]pyrimidin-4-amine **58** (135 mg, 76%) as a yellow solid. LCMS (conditions L-column3 ODS, 50*3.0 mm, 3.0 um, Mobile Phase A: Water (0.05% ammonia water), Mobile Phase B: Acetonitrile, 5%-100%B-3min, ES, m/z): T_R = 2.69 min; $[M+H]^+$:719, 721.

5-(1-benzyl-4-chloro-1*H*-pyrazol-3-yl)-7-((3a*S*,4*R*,6*R*,6a*R*)-6-(3-(((*tert*-butyldimethylsilyl)oxy)methyl)phenyl)-2,2-dimethyltetrahydro-4*H*-cyclopenta[*d*][1,3]dioxol-4-yl)-7*H*-pyrrolo[2,3-*d*]pyrimidine (59)

Into a 8 mL vial were added 7-[(3aS,4R,6R,6aR)-6-(3-{[(tert-butyldimethylsilyl)oxy]methyl}phenyl)-2,2-dimethyl-tetrahydro-3aH-cyclopenta[d][1,3]dioxol-4-yl]-5-iodopyrrolo[2,3-d]pyrimidine (0.2 g, 0.33 mmol, 1.0 eq), 1-benzyl-4-chloropyrazol-3-ylboronic acid (0.23 g, 0.99 mmol, 3.0 eq) and K_3PO_4 (0.14 g, 0.66 mmol, 2.0 eq), Pd(dtbpf)Cl_2 (21.52 mg, 0.03 mmol, 0.1 eq) in 1,4-dioxane (2.0 mL) and H_2O (0.2 mL) at room temperature under N_2 atmosphere. The resulting mixture was stirred for 2 h at 60°C and was concentrated under reduced pressure. The residue was dissolved in DCM (2.0 mL) and was purified by silica gel column chromatography (2:1 Petroleum ether: Tetrahydrofuran) to afford 3-{7-[(3aS,4R,6R,6aR)-6-(3-{[(tert-butyldimethylsilyl)oxy]methyl}phenyl)-2,2-dimethyl-tetrahydro-3aH-cyclopenta[d][1,3]dioxol-4-yl]pyrrolo[2,3-d]pyrimidin-5-yl}-1-benzyl-4-chloropyrazole **59** (0.14 g, 63%) as a yellow solid. LCMS (conditions Halo C18, 30*3.0 mm, 2.0 µm, Mobile Phase A: Water/0.05%TFA, Mobile Phase B: Acetonitrile, 5%-95%B-1.2 min, 1.50 L/min, ES, m/z): T_R = 0.94 min; $[M+H]^+$: 670, 672.

(3-((3aR,4R,6R,6aS)-6-(2-chloro-4-((4-methoxybenzyl)amino)-7H-pyrrolo[2,3-d]pyrimidin-7-yl)-2,2-dimethyltetrahydro-4H-cyclopenta[d][1,3]dioxol-4-yl)phenyl)methanol (60)

Into a 8 mL vial were added 7-((3aS,4R,6R,6aR)-6-(3-(((tert-butyldimethylsilyl)oxy)me-thyl)phenyl)-2,2-dimethyltetrahydro-4H-cyclopenta[d][1,3]dioxol-4-yl)-2-chloro-N-(4-methoxybenzyl)-7H-pyrrolo[2,3-d]pyrimidin-4-amine (0.32 g, 0.4 mmol, 1.0 eq) and 1 M TBAF in THF (1.4 mL, 1.2 mmol, 3.0 eq) in THF (2.0 mL) at room temperature. The resulting mixture was stirred for 1h at room temperature. The resulting mixture was concentrated under reduced pressure. The residue was dissolved in DCM (3.0 mL) and purified by silica gel column chromatography (40:60 Petroleum ether/THF) to afford (3-((3aR,4R,6R,6aS)-6-(2-

chloro-4-((4-methoxybenzyl)amino)-7H-pyrrolo[2,3-d]pyrimidin-7-yl)-2,2-dimethyltetrahydro-4H-cyclopenta[d][1,3]dioxol-4-yl)phenyl)methanol **60** (240 mg, 91%) as a white solid. LCMS (conditions Cortecs C18+, 50*3.0 mm, 2.7 µm, Mobile Phase A: Water/0.05%TFA, Mobile Phase B: Acetonitrile, 2%-100%B-3min, ES, m/z): T_R =1.68 min; $[M+H]^+$: 535, 537.

(3-((3aR,4R,6R,6aS)-6-(4-amino-5-(1-benzyl-4-chloro-1H-pyrazol-3-yl)-2-chloro-7H-pyrrolo[2,3-d]pyrimidin-7-yl)-2,2-dimethyltetrahydro-4H-cyclopenta[d][1,3]dioxol-4-yl)phenyl)methanol (61)

Into a 40mL vial were added 7-[(3aS,4R,6R,6aR)-6-(3-{[(tert-butyldimethylsilyl)oxy]methyl}phenyl)-2,2-dimethyl-tetrahydro-3aH-cyclopenta[d][1,3]dioxol-4-yl]-5-(1-benzyl-4-chloropyrazol-3-yl)-2-chloropyrrolo[2,3-d]pyrimidin-4-amine (130.0 mg, 0.2mmol, 1.0 eq) and THF (3.0 mL) at room temperature, to the above mixture was added TBAF (0.6 mL, 0.6 mmol, 3.0 eq, 1 mol/L in THF) dropwise at room temperature and was stirred for 0.5 h at room temperature. After completion of reaction, the resulting mixture was concentrated under reduced pressure. The residue was purified by silica gel column chromatography (1:1 Petroleum ether/tetrahydrofuran) to afford 7-[(3aS,4R,6R,6aR)-6-(3-{[(tert-butyldimethylsilyl)oxy]methyl}phenyl)-2,2-dimethyl-tetrahydro-3aH-cyclopenta[d][1,3]dioxol-4-yl]-5-(1-benzyl-4-chloropyrazol-3-yl)-2-chloropyrrolo[2,3-d]pyrimidin-4-amine {3-[(3aR,4R,6R,6aS)-6-[4-amino-5-(1-benzyl-4-chloropyrazol-3-yl)-2-chloropyrrolo[2,3-d]pyrimidin-7-yl]-2,2-dimethyl-tetrahydro-3aH-cyclopenta[d][1,3]dioxol-4-yl]phenyl}methanol **61** (70.0 mg, 45%) as a yellow solid. LCMS (conditions L-column3 ODS, 50*3.0 mm, 3.0 um, Mobile Phase A: Water (0.05% ammonia water), Mobile Phase B: Acetonitrile, 5%-100%B-3min, ES, m/z): T_R = 2.09 min; $[M+H]^+$:605, 607.

(3-((3a*R*,4*R*,6*R*,6a*S*)-6-(5-(1-benzyl-4-chloro-1*H*-pyrazol-3-yl)-7*H*-pyrrolo[2,3-*d*]pyrimidin-7-yl)-2,2-dimethyltetrahydro-4*H*-cyclopenta[*d*][1,3]dioxol-4-yl)phenyl)methanol (62)

Into a 8 mL vial were added 3-{7-[(3aS,4R,6R,6aR)-6-(3-{[(tert-butyldimethylsilyl)oxy]methyl}phenyl)-2,2-dimethyl-tetrahydro-3aH-cyclopenta[d][1,3]dioxol-4-yl]pyrrolo[2,3-d]pyrimidin-5-yl}-1-benzyl-4-chloropyrazole (0.14 g, 0.2 mmol, 1.0 eq) and 1M TBAF in THF (0.8 mL, 3.0 eq), THF (1.5 mL) at room temperature. The resulting mixture was stirred for 0.5 h at room temperature and was concentrated under reduced pressure. The residue was purified by silica gel column chromatography (1:1 Petroleum ether: Tetrahydrofuran) to afford {3-[(3aR,4R,6R,6aS)-6-[5-(1-benzyl-4-chloropyrazol-3-yl)pyrrolo[2,3-d]pyrimidin-7-yl]-2,2-dimethyl-tetrahydro-3aH-cyclopenta[d][1,3]dioxol-4-yl]phenyl}methanol **62** (70 mg, 60%) as a yellow solid. LCMS (conditions Halo C18, 30*3.0 mm, 2.0 μm, Mobile Phase A: Water/0.05%TFA, Mobile Phase B: Acetonitrile, 5%-95%B-1.2 min, 1.50 L/min, ES, m/z): T_R = 0.69 min; $[M+H]^+$: 556, 558.

3-((3aR,4R,6R,6aS)-6-(2-chloro-4-((4-methoxybenzyl)amino)-7H-pyrrolo[2,3-d]pyrimidin-7-yl)-2,2-dimethyltetrahydro-4H-cyclopenta[d][1,3]dioxol-4-yl)benzaldehyde (64)

Into a 8 mL vial were added (3-((3aR,4R,6R,6aS)-6-(2-chloro-4-((4-methoxybenzyl)amino)-7H-pyrrolo[2,3-d]pyrimidin-7-yl)-2,2-dimethyltetrahydro-4H-cyclopenta[d][1,3]dioxol-4-yl)phenyl)methanol (0.22 g, 0.4 mmol, 1.0 eq) and manganese dioxide (142 mg, 1.6 mmol, 4.0 eq) in DCM (2.0 mL) at room temperature. The resulting mixture was stirred for overnight at room temperature. The resulting mixture was filtered, the filter cake was washed with DCM (3x5.0 mL). The filtrate was concentrated under reduced pressure to afford 3-((3aR,4R,6R,6aS)-6-(2-chloro-4-((4-methoxybenzyl)amino)-7H-pyrrolo[2,3-d]pyrimidin-7-yl)-2,2-dimethyltetrahydro-4H-cyclopenta[d][1,3]dioxol-4-yl)benzaldehyde **64** (200 mg, 91%) as a white solid. LCMS (conditions Cortecs C18+, 50*3.0 mm, 2.7 μm, Mobile Phase A: Water/0.05%TFA,

Mobile Phase B: Acetonitrile, 2%-100%B-2min, ES, m/z): T_R =1.22 min; $[M+H]^+$: 533, 535.

3-((3aR,4R,6R,6aS)-6-(4-amino-5-(1-benzyl-4-chloro-1H-pyrazol-3-yl)-2-chloro-7H-pyrrolo[2,3-d]pyrimidin-7-yl)-2,2-dimethyltetrahydro-4H-cyclopenta[d][1,3]dioxol-4-yl)benzaldehyde (65)

Into a 8mL vial were added {3-[(3aR,4R,6R,6aS)-6-[4-amino-5-(1-benzyl-4-chloropyrazol-3-yl)-2-chloropyrrolo[2,3-d]pyrimidin-7-yl]-2,2-dimethyl-tetrahydro-3aH-cyclopenta[d][1,3]dioxol-4-yl]phenyl}methanol (70.0 mg, 0.16 mmol, 1.0 eq), MnO_2 (70 mg, 0.8 mmol, 5.0 eq) and DCM (1 mL) at room temperature. The resulting mixture was stirred for 24 h at 40°C. After completion of reaction, the resulting mixture was filtered, the filter cake was washed with DCM (2x3 mL). The filtrate was concentrated under reduced pressure to afford 3-[(3aR,4R,6R,6aS)-6-[4-amino-5-(1-benzyl-4-chloropyrazol-3-yl)-2-chloropyrrolo-[2,3-d]pyrimidin-7-yl]-2,2-dimethyl-tetrahydro-3aH-cyclopenta[d][1,3]dioxol-4-yl]benzal-dehyde **65** (65.0 mg, 93%) as a yellow solid. LCMS (conditions L-column3 ODS, 50*3.0 mm, 3.0 um, Mobile Phase A: Water (0.05% ammonia water), Mobile Phase B: Acetonitrile, 5%-100%B-3min, ES, m/z): T_R = 2.22 min; $[M+H]^+$:603, 605.

3-((3a*R*,4*R*,6*R*,6a*S*)-6-(5-(1-benzyl-4-chloro-1*H*-pyrazol-3-yl)-7*H*-pyrrolo[2,3-*d*]pyrimidin-7-yl)-2,2-dimethyltetrahydro-4*H*-cyclopenta[*d*][1,3]dioxol-4-yl)benzaldehyde (66)

Into a 8 mL vial were added {3-[(3aR,4R,6R,6aS)-6-[5-(1-benzyl-4-chloropyrazol-3-yl)pyrrolo[2,3-d]pyrimidin-7-yl]-2,2-dimethyl-tetrahydro-3aH-cyclopenta[d][1,3]dioxol-4-yl]phenyl}methanol (70 mg, 0.1 mmol, 1.0 eq) and MnO_2 (54.7 mg, 0.5 mmol, 5.0 eq) in DCM (1.5 mL) at room temperature. The resulting mixture was stirred for 4 h at 40°C and was filtered. The filter cake was washed with DCM (3 x 10.0 mL). The filtrate was concentrated under reduced pressure to afford 3-[(3aR,4R,6R,6aS)-6-[5-(1-benzyl-4-chloropyrazol-3-yl)pyrrolo[2,3-d]pyrimidin-7-yl]-

2,2-dimethyl-tetrahydro-3aH-cyclopenta[d][1,3]dioxol-4-yl]benzaldehyde **66** (60 mg, 86%) as a yellow solid. LCMS (conditions Halo C18, 30*3.0 mm, 2.0 μm, Mobile Phase A: Water/0.05%TFA, Mobile Phase B: Acetonitrile, 5%-95%B-1.2 min, 1.50 L/min, ES, m/z): T_R = 0.67 min; $[M+H]^+$: 554, 556.

7-((3aS,4R,6R,6aR)-6-(3-(azetidin-1-ylmethyl)phenyl)-2,2-dimethyltetrahydro-4H-cyclopenta[d][1,3]dioxol-4-yl)-2-chloro-N-(4-methoxybenzyl)-7H-pyrrolo[2,3-d]pyrimidin-4-amine (67)

Into a 8 mL vial were added 3-[(3aR,4R,6R,6aS)-6-(2-chloro-4-{[(4-methoxyphenyl)me-thyl]amino}pyrrolo[2,3-d]pyrimidin-7-yl)-2,2-dimethyl-tetrahydro-3aH-cyclopenta[d][1,3]di-oxol-4-yl]benzaldehyde (120.0 mg, 0.2 mmol, 1.0 equiv), azetidine (38.5 mg, 0.6 mmol, 3.0 equiv) and $NaBH(OAc)_3$ (143.1 mg, 0.6 mmol, 3.0 equiv), AcOH (27.0 mg, 0.4 mmol, 2.0 equiv) in DCM (2.0 mL) at room temperature. The resulting mixture was stirred for 0.5h at room temperature. The resulting mixture was concentrated under reduced pressure. The resulting mixture was diluted with DCM (3.0 mL). The residue was purified by silica gel column chromatography (1:1 Petroleum ether/THF) to afford 7-[(3aS,4R,6R,6aR)-6-[3-(azetidin-1-ylmethyl)phenyl]-2,2-dimethyl-tetrahydro-3aH-cyclopenta[d][1,3]dioxol-4-yl]-2-chloro-N-[(4-methoxyphenyl)methyl]pyrrolo[2,3-d]pyrimidin-4-amine **67** (120.0 mg, 92%) as a white solid. LCMS (conditions L-column3 ODS, 50*3.0 mm, 3.0 um, Mobile Phase A: Water (0.05% ammonia water), Mobile Phase B: Acetonitrile, 5%-100%B-2 min, 1.20 L/min, ES, m/z):T_R =1.48 min; $[M+H]^+$:574, 576.

7-((3aS,4R,6R,6aR)-6-(3-(azetidin-1-ylmethyl)phenyl)-2,2-dimethyltetrahydro-4H-cyclopenta[d][1,3]dioxol-4-yl)-5-(1-benzyl-4-chloro-1H-pyrazol-3-yl)-2-chloro-7H-pyrrolo[2,3-d]pyrimidin-4-amine (68)

Into a 8mL vial were added 3-[(3aR,4R,6R,6aS)-6-[4-amino-5-(1-benzyl-4-chloropyrazol-3-yl)-2-chloropyrrolo[2,3-d]pyrimidin-7-yl]-2,2-dimethyl-tetrahydro-3aH-cyclopenta[d][1,3]dioxol-4-yl]benzaldehyde (65 mg, 0.149 mmol, 1.0 equiv),

AcOH (12.0 mg, 0.2 mmol, 2.0 eq), azetidine (16.8 mg, 0.4 mmol, 3.0 eq) and DCM (2.5 mL) at room temperature. To the above mixture was added $NaBH_3(CN)$ (65.5 mg, 0.447 mmol, 3.0 eq) in portions at room temperature. The final reaction mixture was stirred for 0.5 h at room temperature. After completion of reaction, the resulting mixture was concentrated under reduced pressure and was dissolved in DCM (2.0 mL). The residue was purified by Prep-TLC (10:1 DCM: MeOH) to afford 7-((3aS,4R,6R,6aR)-6-(3-(azetidin-1-ylmethyl)phenyl)-2,2-dimethyltetrahydro-4H-cyclopenta[d][1,3]dioxol-4-yl)-5-(1-benzyl-4-chloro-1H-pyrazol-3-yl)-2-chloro-7H-pyrrolo[2,3-d]pyrimidin-4-amine **68** (42 mg, 60%) as a yellow solid. LCMS (conditions L-column3 ODS, 50*3.0 mm, 3.0 um, Mobile Phase A: Water (0.05% ammonia water), Mobile Phase B: Acetonitrile, 5%-100%B-3min, ES, m/z): T_R = 2.32 min; $[M+H]^+$:644, 646.

5-(1-benzyl-4-chloro-1*H*-pyrazol-3-yl)-7-((3a*S*,4*R*,6*R*,6a*R*)-6-(3-((3,3-difluoroazetidin-1-yl)methyl)phenyl)-2,2-dimethyltetrahydro-4*H*-cyclopenta[*d*][1,3]dioxol-4-yl)-7*H*-pyrrolo[2,3-*d*]pyrimidine (69)

Into a 8 mL vial were added 3-[(3aR,4R,6R,6aS)-6-[5-(1-benzyl-4-chloropyrazol-3-yl)pyrrolo[2,3-d]pyrimidin-7-yl]-2,2-dimethyl-tetrahydro-3aH-cyclopenta[d][1,3]dioxol-4-yl]benzaldehyde (60 mg, 0.1 mmol, 1.0 eq), 3,3-difluoroazetidine (20.1 mg, 0.2 mmol, 2.0 eq) and DIEA (27.9 mg, 0.2 mmol, 2.0 eq), $NaBH_3CN$ (13.6 mg, 0.2 mmol, 2.0 eq) in DCM (0.6 mL) at room temperature. The resulting mixture was stirred for 0.5 h at room temperature and was concentrated under reduced pressure. The residue was dissolved in DCM (2.0 mL) and was purified by silica gel column chromatography (12:1 DCM: MeOH) to afford 3-{7-[(3aS,4R,6R,6aR)-6-{3-[(3,3-difluoroazetidin-1-yl)methyl]phenyl}-2,2-dimethyl-tetrahydro-3aH-cyclopenta[d][1,3]dioxol-4-yl]pyrrolo[2,3-d]pyrimidin-5-yl}-1-benzyl-4-chloropyrazole **69** (50 mg, 73%) as a white solid. LCMS (conditions Halo C18, 30*3.0 mm, 2.0 µm, Mobile Phase A: Water/0.05%TFA, Mobile Phase B: Acetonitrile, 5%-95%B-1.2 min, 1.50 L/min, ES, m/z): T_R = 0.54 min; $[M+H]^+$: 631, 633.

(1*R*,2*S*,3*R*,5*R*)-3-(4-amino-7*H*-pyrrolo[2,3-*d*]pyrimidin-7-yl)-5-phenylcyclopentane-1,2-diol (70a, 2a)

Following the general procedure **G** for deprotection, **70a** was obtained starting from compound **46** (0.02 g, 0.003 mmol) in a mixture of water and TFA (0.03 M, 1.00 mL, 1:4) after preparative HPLC using method **D** (water/MeCN; 0.05% TFA) as white foam (15 mg, 83%). ^{1}H NMR (400 MHz, DMSO-d_6) δ 8.06 (s, 1H, H2), 7.41 – 7.30 (m, 5H, H5, *o*, *m*-Ar), 7.25 – 7.19 (m, 1H, *p*-Ar), 6.94 (s, 2H, -CN*H*$_2$), 6.56 (d, *J* = 3.5 Hz, 1H, H5), 4.97 (d, *J* = 5.9 Hz, 1H, 3′OH), 4.92 – 4.88 (m, 1H, H1′), 4.87 (d, *J* = 6.3 Hz, 1H, 2′OH), 4.28 (d, *J* = 6.3 Hz, 1H, H2′), 4.13 – 4.05 (m, 1H, H3′), 3.08 (dt, *J* = 11.8, 7.3 Hz, 1H, H4′), 2.34 (dt, *J* = 12.4, 7.6 Hz, 1H, H5′), 2.01 (q, *J* = 11.9 Hz, 1H, H5′). APCI: calc. for $C_{17}H_{19}N_4O_2$ [M+H]$^+$: 311.14, found: 311.3 [M+H]$^+$. HPLC: t_R = 12.769 min (Method B). UV-purity at 254 nm = 100.0%.

(1*R*,2*S*,3*R*,5*R*)-3-(4-amino-7*H*-pyrrolo[2,3-*d*]pyrimidin-7-yl)-5-(pyridin-4-yl)cyclopentane-1,2-diol (70b, 2b)

Following the general procedure **L** for deprotection, **70b** was obtained starting from compound **47** (0.02 g, 0.06 mmol) in a mixture of water and TFA (0.05 M, 10.90 mL, 1:4) after preparative HPLC using method F (water/MeCN; 0.05% TFA) as white foam (16.20 mg, 89%). ^{1}H NMR (400 MHz, DMSO-d_6) δ 8.83 – 8.79 (m, 2H, H2, H6 pyridine), 8.41 (s, 1H, H2 adenine), 7.96 – 7.92 (m, 2H, H3, H5 pyridine), 7.80 (d, *J* = 3.6 Hz, 1H, H6 adenine), 6.99 (d, *J* = 3.6 Hz, 1H, H5 adenine), 5.05 (dt, *J* = 11.0, 7.3 Hz, 1H, H1′), 4.26 (t, *J* = 6.3 Hz, 1H, H2′), 4.17 (t, *J* = 7.1 Hz, 1H, H3′), 3.38 (dt, *J* = 11.8, 7.2 Hz, 1H, H4′), 2.54 – 2.44 (m, 1H, H5′ under DMSO peak), 2.18 – 2.07 (m, 1H, H5′). HRMS: calc. for $C_{16}H_{18}N_5O_2$ [M+H]$^+$: 312.14, found: 312.1458 [M+H]$^+$. HPLC: t_R = 5.617 min (Method D). UV-purity at 254 nm = 100%.

(1*R*,2*S*,3*R*,5*S*)-3-(4-amino-7*H*-pyrrolo[2,3-*d*]pyrimidin-7-yl)-5-(thiophen-2-yl)cyclopentane-1,2-diol (70c, 2c)

Following the general procedure **G** for deprotection, **70c** was obtained starting from compound **48** (0.02 g, 0.05 mmol) in a mixture of water and TFA (0.03 M, 1.60 mL, 1:4) after preparative HPLC using method **F** (water/MeCN; 0.05% TFA) as white foam (10 mg, 56%). ^{1}H NMR (400 MHz, DMSO-d_6) δ 8.39 (s, 1H, H2), 7.71 (d, J = 3.7 Hz, 1H, H6), 7.38 (dd, J = 4.9, 1.4 Hz, 1H, H5 thiophene), 7.03 – 6.98 (m, 2H, H3, H4 thiophene), 6.96 (d, J = 3.6 Hz, 1H, H5), 4.99 (dt, J = 10.9, 7.3 Hz, 1H, H1′), 4.26 (t, J = 6.7 Hz, 1H, H2′), 4.03 (t, J = 6.5 Hz, 1H, H3′), 3.35 (dt, J = 11.0, 7.0 Hz, 1H, H4′), 2.59 – 2.51 (m, 1H, H5′), 2.05 (dt, J = 12.4, 11.0 Hz, 1H, H5′). APCI: calc. for $C_{15}H_{17}N_4O_2S$ $[M+H]^+$: 317.10, found: 317.2 $[M+H]^+$. HPLC: t_R = 12.673 min (Method C). UV-purity at 254 nm = 100.0%.

(1*R*,2*S*,3*R*,5*R*)-3-(4-amino-7*H*-pyrrolo[2,3-*d*]pyrimidin-7-yl)-5-(1*H*-pyrazol-5-yl)cyclopentane-1,2-diol (70d, 2d)

Following the general procedure **G** for deprotection, **70d** was obtained starting from compound **49** (0.03 g, 0.07 mmol) in a mixture of water and TFA (0.05 M, 1.50 mL, 1:4) after preparative HPLC using method **F** (water/MeCN; 0.05% TFA) as white foam (17.30 mg, 79%). ^{1}H NMR (400 MHz, DMSO-d_6) δ 8.39 (s, 1H, H2), 7.87 (dd, J = 2.3, 0.7 Hz, 1H, H3 pyrazol), 7.83 (d, J = 3.6 Hz, 1H, H6), 7.56 (d, J = 1.7 Hz, 1H, -N*H* pyrazol), 7.00 (d, J = 3.6 Hz, 1H, H5), 6.29 (dd, J = 1.9 Hz, 1H, H4 pyrazol), 5.12 – 5.04 (m, 1H, H1′), 4.68 (td, J = 8.2, 4.5 Hz, 1H, H4′), 4.42 (dd, J = 7.2, 5.8 Hz, 1H, H2′), 4.13 – 4.09 (m, 1H, H3′), 2.75 (dt, J = 13.6, 8.7 Hz, 1H, H5′), 2.39 – 2.31 (m, 1H, H5′). APCI: calc. for $C_{14}H_{17}N_6O_2$ $[M+H]^+$: 300.13; found: 300.9 $[M+H]^+$. HPLC: t_R = 8.818 min (Method B). UV-purity at 254 nm = 100%.

(1R,2S,3R,5R)-3-{4-Aminopyrrolo[2,3-d]pyrimidin-7-yl}-5-(1H-pyrazol-4-yl)cyclopentane-1,2-diol (70e, 2e)

Into an 8 mL vial were added 7-[(3aS,4R,6R,6aR)-2,2-dimethyl-6-[1-(oxan-2-yl)pyrazol-4-yl]-tetrahydro-3aH-cyclopenta[d][1,3]dioxol-4-yl]-N-[(4-methoxyphenyl)methyl]pyrrolo[2,3-d]pyrimidin-4-amine (80 mg, 0.15 mmol, 1.0 eq) and TFA (2 mL) at room temperature. The resulting mixture was stirred for 0.5 h at 80°C, then concentrated. The residue was dissolved in MeOH (3 mL) and basified to pH>8 with NH_3•H_2O. The mixture was purified by Prep-HPLC [column, Xbridge Prep C18 OBD column, 5um, 19*150mm; mobile phase, Water (0.03% NH_4OH) and CH_3CN (20-55% CH_3CN over 10 min); Detector, UV 220&254 nm] to afford (1R,2S,3R,5R)-3-{4-aminopyrrolo[2,3-d]pyrimidin-7-yl}-5-(1H-pyrazol-4-yl)cyclopentane-1,2-diol **70e** (13.9 mg, 32%) as a white solid. LCMS (conditions: ZORBAX SB-Aq, 50*4.6 mm, 1.8 µm, Mobile Phase A: Water/0.02% TFA, Mobile Phase B: Acetonitrile, 5% - 95% B - 3 min, 1.50 mL/min): T_R = 0.94 min; ES m/z $[M+H]^+$: 301. ^{1}H NMR (400 MHz, DMSO-*d*$_6$) δ 8.05 (s, 1H), 7.55 (s, 2H), 7.27 (d, *J* = 3.5 Hz, 1H), 6.92 (s, 2H), 6.56 (d, *J* = 3.5 Hz, 1H), 4.94 - 4.80 (m, 3H), 4.25-4.20 (m, 1H), 3.97-3.92 (m, 1H), 3.05-3.01 (m, 1H), 2.46-2.35 (m, 1H), 1.89 (q, *J* = 10.9 Hz, 1H). UV-purity at 254 nm = 99.3%.

(1*R*,2*S*,3*R*,5*R*)-3-(4-amino-7*H*-pyrrolo[2,3-*d*]pyrimidin-7-yl)-5-(3-aminophenyl)cyclopentane-1,2-diol (70f, 2f)

Following the general procedure **G** for deprotection, **70f** was obtained starting from compound **51** (0.27 g, 0.36 mmol) in a mixture of water and TFA (0.10 M, 3.60 mL, 1:4) after preparative HPLC using method **E** (water/MeCN; 0.05% TFA) as white foam (118 mg, quant.). ^{1}H-NMR (400 MHz, DMSO-d$_6$) δ = 9.16 (bs, 1H), 8.73 (bs, 1H), 8.41 (s, 1H), 7.78 (d, *J* = 3.6 Hz, 1H), 7.30-7.26 (m, 1H), 7.13-7.03 (m, 2H), 6.98 (d, *J* = 3.6 Hz, 1H), 6.94-6.88 (m, 1H), 5.03-4.97 (m, 1H), 4.30 (t, *J* = 6.9 Hz, 1H), 4.03 (t, *J* = 6.6 Hz, 1H), 3.17-3.05 (m, 1H), 2.43-2.36 (m, 1H), 2.06-1.97 (m, 1H) ppm. APCI-

MS(+) m/z for $C_{17}H_{19}N_5O_2$: calc.: 325.37; found: 325.8 and 326.8.HPLC: t_R = 7.869 min (method C). UV-purity at 254 nm = 100%.

(1R,2S,3R,5R)-3-(4-Amino-7H-pyrrolo[2,3-d]pyrimidin-7-yl)-5-(3-(aminomethyl)phenyl) cyclopentane-1,2-diol (70g, 2g)

Into 40 mL vial were added tert-butyl (3-((3aR,4R,6R,6aS)-6-(4-((4-methoxybenzyl)amino)-7H-pyrrolo[2,3-d]pyrimidin-7-yl)-2,2-dimethyltetrahydro-4H-cyclopenta[d][1,3]dioxol-4-yl)benzyl)carbamate (60 mg, 0.1 mmol, 1.0 eq), TFA (2.0 mL) and DCM (2.0 mL) at room temperature. The reaction mixture was stirred at 80°C for 1 h, then allowed to cool to room temperature and concentrated under reduced pressure. The residue was dissolved in MeOH (3.0 mL) and basified to pH>8 with $NH_3 \cdot H_2O$. The mixture was purified by reverse phase flash chromatography with the following conditions (Column: Welch Utimate AQ-C18, 50*250mm*10μm; Mobile Phase A: Water(10 mmol/L NH_4HCO_3+0.1%$NH_3.H_2O$), Mobile Phase B: ACN; Flow rate: 80 mL/min; Gradient: 5% B to 60% B in 15 min, 60% B; Wavelength: 220 nm) to afford (1R,2S,3R,5R)-3-(4-amino-7H-pyrrolo[2,3-d]pyrimidin-7-yl)-5-(3-(aminomethyl)phenyl)cyclopentane-1,2-diol **70g** (10.8 mg, 31%) as a white solid. LCMS (ZORBAX SB-Aq, 50*4.6 mm, 1.8 μm, Mobile Phase A: Water/0.02% TFA, Mobile Phase B: Acetonitrile, 5%-95%B - 3 min, 1.50 L/min): T_R = 0.93 min; ES m/z $[M+H]^+$: 340. ^{1}H NMR (300 MHz, DMSO-*d*6+D_2O) δ 8.06 (s, 1H), 7.40-7.10 (m, 5H), 6.58 (d, *J* = 3.6 Hz, 1H), 5.00-4.77 (m, 1H), 4.29 (t, *J* = 6.7 Hz, 1H), 4.14 (s, 1H), 4.06 (t, *J* = 6.7 Hz, 1H), 3.71 (d, *J* = 2.6 Hz, 1H), 3.15-2.99 (m, 1H), 2.39-2.28 (m, 1H), 2.09-1.88 (m, 1H). MS: calc. for $C_{18}H_{21}N_5O_2$ $[M]^+$: 339.17, found: 339.2 $[M]^+$. UV-purity at 254 nm = 99.8%.

(1S,2R,3R,5R)-3-[3-(2-Aminoethyl)phenyl]-5-{4-aminopyrrolo[2,3-d]pyrimidin-7-yl}cyclopentane-1,2-diol (70h, 2h)

Into an 8 mL vial were added tert-butyl N-(2-{3-[(3aR,4R,6R,6aS)-6-(4-{[(4-methoxyphenyl)methyl]amino}pyrrolo[2,3-d]pyrimidin-7-yl)-2,2-dimethyl-

tetrahydro-3aH-cyclopenta[d][1,3]dioxol-4-yl]phenyl}ethyl)carbamate (99 mg, 0.16 mmol, 1.0 eq) and TFA (0.5 mL) in DCM (0.5 mL). The resulting mixture was stirred for 0.5 h at 80 °C, then concentrated. The residue was dissolved in MeOH (3 mL). The mixture was basified to pH > 8 with $NH_3{\cdot}H_2O$ and was purified by Prep-HPLC [column, Xbridge Prep C18 OBD column, 5um, 19*150mm; mobile phase, Water (0.03% NH_4OH) and CH_3CN (20 - 40% over 10 min); Detector, UV 220&254 nm] to afford (1S,2R,3R,5R)-3-[3-(2-aminoethyl)phenyl]-5-{4-aminopyrrolo[2,3-d]pyrimidin-7-yl}cyclopentane-1,2-diol **70h** (10.4 mg, 18%) as an off-white solid. LCMS (conditions: ZORBAX SB-Aq, 50*4.6 mm, 1.8 μm, 5% - 95% Acetonitrile / 0.02% aqueous TFA - 3 min, 1.50 mL/min): T_R = 0.98 min; ES m/z $[M+H]^+$: 354. ^1H NMR (400 MHz, DMSO-d_6+D_2O) δ 8.07 (s, 1H), 7.36 (d, *J* = 3.6 Hz, 1H), 7.25-7.21 (m, 3H), 7.06 (d, *J* = 6.9 Hz, 1H), 6.57 (d, *J* = 3.5 Hz, 1H), 4.94-4.83 (m, 1H), 4.30 (t, *J* = 6.7 Hz, 1H), 4.06 (t, *J* = 6.7 Hz, 1H), 3.21-3.12 (m, 2H), 3.12-3.01 (m, 1H), 2.78-2.60 (m, 2H), 2.40-2.28 (m, 1H), 2.02 (q, *J* = 11.7 Hz, 1H). MS: calc. for $C_{19}H_{23}N_5O_2$ $[M]^+$: 353.19, found: 353.2 $[M]^+$. UV-purity at 254 nm = 98.0%.

(1R,2S,3R,5R)-3-{4-Amino-2-chloropyrrolo[2,3-d]pyrimidin-7-yl}-5-[3-(aminomethyl)phenyl]cyclopentane-1,2-diol (70i, 3a)

Into a 40 mL vial was placed a solution of tert-butyl N-({3-[(3aR,4R,6R,6aS)-6-{4-amino-2-chloropyrrolo[2,3-d]pyrimidin-7-yl}-2,2-dimethyl-tetrahydro-3aH-cyclopenta[d][1,3]dioxol-4-yl]phenyl}methyl)carbamate (0.15 g, 0.3 mmol, 1.0 eq) in 4M HCl in MeOH (2.0 mL). The mixture was stirred for 20 min at 50°C. The resulting mixture was concentrated under vacuum. The residue was dissolved in MeOH (2.0 mL) and basified to pH>8 with Na_2CO_3. The resulting mixture was filtered and the filter cake washed with MeOH (3 x 1 mL). The combined filtrate was purified by Prep-HPLC (Column: Spherical C18, 20-40μm,120g; Mobile Phase A: Water/5mM NH_4HCO_3+0.05% Ammonia, Mobile Phase B: ACN; Flow rate: 80 mL/min; Gradient: 10% B to 35% B over 10 min; Wavelength: 254 nm; Number Of Runs:2) to afford (1R,2S,3R,5R)-3-{4-amino-2-chloropyrrolo[2,3-d]pyrimidin-7-yl}-5-[3-

(aminomethyl)phenyl]cyclopentane-1,2-diol **70i** (80 mg, 73%) as an off-white solid. LCMS (Cortecs C18+, 50*3.0 mm, 2.7 μm, Mobile Phase A: Water/0.02%TFA, Mobile Phase B: Acetonitrile, 5%-95%B - 3 min, 1.50 L/min): T_R = 0.68 min; ES m/z $[M+H]^+$: 374, 376. ^{1}H NMR (400 MHz, DMSO-d_6) δ 7.41-7.36 (m, 1H), 7.35-7.22 (m, 3H), 7.21-7.15 (m, 1H), 6.60 (d, J = 3.5 Hz, 1H), 4.86-4.76 (m, 1H), 4.26 (t, J = 6.8 Hz, 1H), 4.03 (t, J = 6.3 Hz, 1H), 3.70 (s, 2H), 3.13-3.02 (m, 1H), 2.40-2.28 (m, 1H), 1.97 (q, J = 11.5 Hz, 1H). MS: calc. for $C_{18}H_{20}ClN_5O_2$ $[M]^+$: 373.13, found: 373.1 $[M]^+$. UV-purity at 254 nm = 99.2%.

(1R,2S,3R,5R)-3-(4-amino-2-chloro-7H-pyrrolo[2,3-d]pyrimidin-7-yl)-5-(3-(azetidin-1-ylmethyl)phenyl)cyclopentane-1,2-diol (70j, 3b)

Into a 8 mL vial were added 7-[(3aS,4R,6R,6aR)-6-[3-(azetidin-1-ylmethyl)phenyl]-2,2-dimethyl-tetrahydro-3aH-cyclopenta[d][1,3]dioxol-4-yl]-2-chloro-N-[(4-methoxyphenyl)methyl]pyrrolo[2,3-d]pyrimidin-4-amine (60.0 mg, 0.1 mmol, 1.0 equiv) and TFA (2.0 mL) at room temperature. The resulting mixture was stirred for 0.5h at 60°C. After completion of reaction, the resulting mixture was concentrated under reduced pressure and was basified to pH>8 with NH_3•H_2O and was purified by Prep-HPLC with the following conditions (Column: Column: YMC-Actus Triart C18, 30*150 mm, 5μm; Mobile Phase A: Water(10 mmol/L NH_4HCO_3+0.1%NH_3•H_2O), Mobile Phase B: ACN; Flow rate: 30 mL/min; Gradient: 20% B to 55% B in 7 min) to afford to afford (1R,2S,3R,5R)-3-{4-amino-2-chloropyrrolo[2,3-d]pyrimidin-7-yl}-5-[3-(azetidin-1-ylmethyl)phenyl]cyclopentane-1,2-diol **70j** (12.7 mg, 29%) as an off-white solid. LCMS (conditions Xselect CSH Fluoro-Phenyl, 100*3.0 mm, 2.5 μm Mobile Phase A: Water/0.05%TFA, Mobile Phase B: Acetonitrile/0.05%TFA, 5%-40%-95%B-7min, 1.00 mL/min, ES, m/z): T_R =2.228 min; $[M+H]^+$: 414, 416. ^{1}H NMR (400 MHz, DMSO-d_6) δ 7.45 (s, 2H), 7.40 (d, J = 3.5 Hz, 1H), 7.31-7.21 (m, 3H), 7.15-7.08 (m, 1H), 6.60 (d, J = 3.6 Hz, 1H), 4.93 (d, J = 6.1 Hz, 1H), 4.88 (d, J = 6.1 Hz, 1H), 4.87-4.76 (m, 1H), 4.26 (q, J = 6.5 Hz, 1H), 4.03 (q, J = 6.4 Hz, 1H), 3.52 (s, 2H), 3.13 (t, J = 6.9 Hz, 4H), 3.09-3.01 (m, 1H), 2.40-2.29 (m, 1H), 2.04-1.89 (m, 3H). MS:

calc. for $C_{21}H_{24}ClN_5O_2$ $[M]^+$: 413.16, found: 413.2 $[M]^+$. UV-purity at 254 nm = 98.2%.

pyrrolo[2,3-d]pyrimidin-7-yl)-5-(3-(azetidin-1-ylmethyl)phenyl)cyclopentane-1,2-diol (70k, KMI169)

Into a 40mL vial were added 7-[(3aS,4R,6R,6aR)-6-[3-(azetidin-1-ylmethyl)phenyl]-2,2-dimethyl-tetrahydro-3aH-cyclopenta[d][1,3]dioxol-4-yl]-5-(1-benzyl-4-chloropyrazol-3-yl)-2-chloropyrrolo[2,3-d]pyrimidin-4-amine (42.0 mg, 0.11 mmol, 1.0 eq), MeOH (1mL) and TFA (2.0 mL) at room temperature. The resulting mixture was stirred for 0.5 h at 50°C and was monitored by LCMS. After completion of reaction, the resulting mixture was concentrated under reduced pressure. The crude product was purified by Prep-HPLC with the following conditions (Column: XBridge C18, 19*150 mm, 5 μm; Mobile Phase A: 20mM NH_4HCO_3+0.05%NH_3H_2O, Mobile Phase B: ACN; Flow rate: 20 mL/min; Gradient: 10% to 65% in 8min.) to afford (1R,2S,3R,5R)-3-[4-amino-5-(1-benzyl-4-chloropyrazol-3-yl)-2-chloropyrrolo[2,3-d]pyrimidin-7-yl]-5-[3-(azetidin-1-ylmethyl)phenyl]cyclopentane-1,2-diol **70k** (13.5 mg, 34%) as a white solid. LCMS (conditions L-Column ZORBAX SB-Aq, 50*4.6 mm, 1.8 um, Mobile Phase A: Water/0.05%TFA, Mobile Phase B: Acetonitrile/0.05%TFA, 5%-95%B-3min, ES, m/z): T_R = 2.32 min; $[M+H]^+$:604, 606. ^{1}H NMR (400 MHz, DMSO-d_6) δ 8.85 (br, s, 1H), 8.29 (s, 1H), 7.96 (s, 1H), 7.70 (br, s, 1H), 7.40-7.24 (m, 8H), 7.14-7.08 (m, 1H), 5.37 (s, 2H), 5.01-4.87 (m, 3H), 4.38-4.29 (m, 1H), 4.09-4.01 (m, 1H), 3.51 (s, 2H), 3.15-3.07 (m, 5H), 2.45-2.37 (m, 1H), 2.05-1.93 (m, 3H). MS: calc. for $C_{31}H_{31}Cl_2N_7O_2$ $[M]^+$: 603.19, found: 603.2 $[M]^+$. UV-purity at 254 nm = 99.3%.

(1*R*,2*S*,3*R*,5*R*)-3-(5-(1-benzyl-4-chloro-1*H*-pyrazol-3-yl)-7*H*-pyrrolo[2,3-*d*]pyrimidin-7-yl)-5-(3-((3,3-difluoroazetidin-1-yl)methyl)phenyl)cyclopentane-1,2-diol (70l, KMI169Ctrl)

Into a 8 mL vial were added 3-{7-[(3aS,4R,6R,6aR)-6-{3-[(3,3-difluoroazetidin-1-yl)methyl]phenyl}-2,2-dimethyl-tetrahydro-3aH-cyclopenta[d][1,3]dioxol-4-yl]pyrrolo[2,3-d]pyrimidin-5-yl}-1-benzyl-4-chloropyrazole (50 mg, 0.08 mmol, 1.0 eq) and TFA (0.5 mL) at room temperature. The resulting mixture was stirred for 0.5 h at 40°C. The reaction was monitored by LCMS. After completion of reaction, the resulting mixture was concentrated under reduced pressure and was basified to pH>8 with NH_3•H_2O. The residue was purified by Prep-HPLC [column, Xbridge Prep C18 OBD column, 5um, 19*150mm; mobile phase, Water (0.03% NH_4OH) and CH_3CN (20% CH_3CN up to 60% in 10 min); Detector, UV 220&254 nm] to afford (1R,2S,3R,5R)-3-[5-(1-benzyl-4-chloropyrazol-3-yl)pyrrolo[2,3-d]pyrimidin-7-yl]-5-{3-[(3,3-difluoroazetidin-1-yl)methyl]phenyl}cyclopentane-1,2-diol **70l** or **KMI169Ctrl** (10.1 mg, 22%) as a white solid. LCMS (conditions Cortecs C18+, 30*3.0 mm, 2.7 μm, Mobile Phase A: Water/0.05%TFA, Mobile Phase B: Acetonitrile/0.05%TFA, 5%-95%B-2 min, 1.50 L/min, ES, m/z): T_R = 0.72 min; $[M+H]^+$: 591, 593. ^{1}H NMR (400 MHz, DMSO-d_6) δ 9.39 (s, 1H), 8.88 (s, 1H), 8.27 (s, 1H), 8.24 (s, 1H), 7.44-7.26 (m, 8H), 7.16 (d, *J* = 7.0 Hz, 1H), 5.40 (s, 2H), 5.16-5.03 (m, 2H), 4.95 (d, *J* = 5.9 Hz, 1H), 4.54-4.44 (m, 1H), 4.16-4.07 (m, 1H), 3.73 (s, 2H), 3.60 (t, *J* = 12.5 Hz, 4H), 3.19-3.08 (m, 1H), 2.48-2.39 (m, 1H), 2.28-2.15 (m, 1H). UV-purity at 254 nm = 99.3%.

(S)-3-(((benzyloxy)carbonyl)amino)-4-(tert-butoxy)-4-oxobutanoic acid (72)

To a solution of compound **71** (7.0 g, 37 mmol) in 70 mL/70 mL of dioxane/H_2O was added $NaHCO_3$ (9.3 g, 111 mmol) at room temperature. After stirring for 2 hours, CbzCl (68 mL, 48 mmol) was added at ice-water bath. The reaction mixture was stirred at room temperature overnight. The reaction was diluted with H_2O (50 mL x 2) and extracted with DCM (50 mLx 3). The combined organic phase was washed with brine

(50 mL x 2), dried over anhydrous Na_2SO_4, filtered and concentrated to give compound **72** (2.7 g, 22.5% yield) as yellow oil which was used to the next step without further purification. MS Calc.: 323; MS Found: 324 $[M+H]^+$.

(S)-tert-butyl 2-(((benzyloxy)carbonyl)amino)-4-hydroxybutanoate (73)

To a solution of compound **72** (1.7 g, 5.26 mmol) in THF (20 mL) was added $BH_3{\cdot}DMS$ (10 M, 1.2 mL, 2.3 mmol) at 0 ºC. The mixture was stirred at room temperature for 5 hours. The reaction mixture was quenched with MeOH (10 mL) and concentrated to get the crude product. The residue was purified by flash chromatography on silica gel (PE/EA = 2/1) to give compound **73** (780 mg, 48% yield) as yellow oil. MS Calc.: 309; MS Found: 310 $[M+H]^+$.

(S)-tert-butyl 2-(((benzyloxy)carbonyl)amino)-4-oxobutanoate (74)

A solution of compound **73** (780 mg, 2.52 mmol) in DCM (10 mL) was added PCC (1.63 g, 7.56 mmol). The reaction was stirred at room temperature for 4 hours. The reaction mixture was diluted with silica gel (2.0 g), filtered and the residue was concentrated to give compound **74** (580 mg, 75% yield) as yellow oil which was used to the next step without further purification. MS Calc.: 307; MS Found: 308 $[M+H]^+$.

tert-butyl 3-((((3aR,4R,6R,6aR)-6-(6-amino-9H-purin-9-yl)-2,2-dimethyltetrahydrofuro[3,4-d][1,3]dioxol-4-yl)methyl)amino)pyrrolidine-1-carboxylate (76)

To a solution of compound **75** (500 mg, 1.63 mmol) and tert-butyl 3-oxopyrrolidine-1-carboxylate (453 mg, 2.45 mmol) in MeOH (10 mL) and AcOH (0.1 mL) was added $NaBH_3CN$ (154 mg, 2.45 mmol). The reaction mixture was stirred at room temperature for two days. The reaction mixture was concentrated, and the residue was purified by flash chromatography on reverse phase silica gel (ACN/H_2O = 5% - 95%, 254 nm, 30 min) to give compound **76** (200 mg, 26% yield) as yellow oil. MS Calcc.: 475; MS Found: 476 $[M+H]^+$.

tert-butyl 3-((((3aR,4R,6R,6aR)-6-(6-amino-9H-purin-9-yl)-2,2-dimethyltetrahydrofuro[3,4-d][1,3]dioxol-4-yl)methyl)(3-(((benzyloxy)carbonyl)amino)-4-(tert-butoxy)-4-oxobutyl)amino)pyrrolidine-1-carboxylate (77)

To a solution of compound **76** (150 mg, 0.32 mmol) and compound **74** (126 mg, 0.41 mmol) in MeOH (10 mL) and AcOH(0.1 mL)was added $NaBH_3CN$ (154 mg, 2.45 mmol). The reaction mixture was stirred at 35 ºC for two days. The reaction mixture was concentrated and the residue was purified by flash chromatography on silica gel (DCM/MeOH = 10/1) to give compound **77** (140 mg, 58% yield) as yellow oil. MS Calc.: 766; MS Found: 767 $[M+H]^+$

(2S)-2-amino-4-((((2R,3S,4R,5R)-5-(6-amino-9H-purin-9-yl)-3,4-dihydroxytetrahydrofuran-2-yl)methyl)(pyrrolidin-3-yl)amino)butanoic acid (78, 1b)

To a solution of compound **77** (140 mg, 0.18 mmol) in ACOH (3 mL) was added a solution of HBr (30% in AcOH, 1 mL). The reaction mixture was stirred at room temperature for 4 hours. The reaction mixture was concentrated. The residue was dissolved in 2 mL of MeOH and adjusted pH = 7 with Na_2CO_3 solution. The residue was concentrated and purified by prep-HPLC (with additive agent of TFA) to give compound **78**, **1b** (15 mg, 18% yield) as a white solid. ^{1}H NMR (400 MHz, CD_3OD) δ: 8.27 (d, J = 7.2 Hz, 2H), 5.93 (d, J = 2.8 Hz, 1H), 4.60-4.56 (m, 1H), 4.22-4.11 (m, 2H), 3.95 (t, J = 6.0 Hz, 1H), 3.70-3.63 (m, 1H), 3.38-3.26 (m, 2H), 3.16-3.03 (m, 2H), 2.96-2.93 (m, 2H), 2.86-2.80 (m, 2H), 2.16-2.03 (m, 2H), 1.96-1.84 (m, 2H). MS Calc.: 436; MS Found: 437 $[M+H]^+$. UV-purity at 254 nm = >95%.

tert-butyl 2-(2-hydroxyethyl)pyrrolidine-1-carboxylate (80)

To a solution of compound **79** (500 mg, 2.18 mmol) in 20 mL of THF was added $BH_3{\cdot}BS$ (10M in DMSO, 0.33 mL, 3.27 mmol) at 0 ºC. The reaction mixture was stirred at 50 ºC overnight. The reaction mixture was diluted MeOH (10 mL), concentrated and

the residue was purified by flash chromatography on reverse phase silica gel (ACN/H_2O = 5% - 95%, 214 nm, 30 min) to give compound **80** (305 mg, 65% yield) as yellow oil. MS Calc.: 215; MS Found: 216 $[M+H]^+$.

tert-butyl 2-(2-oxoethyl)pyrrolidine-1-carboxylate (81)

To a solution of alcohol **80** (305 mg, 451 mmol) in dry DCM (20 mL) was added PCC (451 mg, 2.09 mmol). The reaction mixture was stirred at room temperature for 4 hours. The reaction mixture was diluted with H_2O (20 mL) and the phases were separated. The organic phase was washed with brine (20 mL x 2), dried over Na_2SO_4, filtered and concentrated to give compound **81** (300 mg, 100% yield) as a brown solid which was used to the next step without further purification. MS Calc.: 213; MS Found: 214 $[M+H]^+$.

(S)-tert-butyl 4-((((3aR,4R,6R,6aR)-6-(6-amino-9H-purin-9-yl)-2,2-dimethyltetrahydrofuro[3,4-d][1,3]dioxol-4-yl)methyl)amino)-2-((tert-butoxycarbonyl)amino)butanoate (82)

A solution of **75** (1.0 g, 3.27 mmol) in 30 mL of MeOH was added compound **6** (0.98 g, 3.59 mmol). After stirring for 5 minutes, $NaBH_3CN$ (411 mg, 6.54 mmol) was added at ice-water bath. The reaction mixture was stirred at 65 °C overnight. The reaction mixture was concentrated and the residue was purified by flash chromatography on reverse phase silica gel (ACN/H_2O = 5% - 95%, 254 nm, 30 min) to give compound **82** (0.66 g, 36% yield) as a white solid. MS Calc.: 563; MS Found: 564 $[M+H]^+$.

***tert*-butyl 2-(2-((((3a*R*,4*R*,6*R*,6a*R*)-6-(6-amino-9*H*-purin-9-yl)-2,2-dimethyltetrahydrofuro[3,4-*d*][1,3]dioxol-4-yl)methyl)((*S*)-4-(*tert*-butoxy)-3-((*tert*-butoxycarbonyl)amino)-4-oxobutyl)amino)ethyl)pyrrolidine-1-carboxylate (83)**

A solution of compound **82** (100 mg, 0.18 mmol) and **81** (49 mg, 0.23 mmol) in MeOH (10 mL) and AcOH (0.1 mL) was stirred for 5 minutes. $NaBH_3CN$ (23 mg, 0.36 mmol) was added and stirred at 65 °C overnight. The reaction mixture was concentrated and

the residue was purified by flash chromatography on reverse phase silica gel (ACN/H_2O = 5% - 95%, 254 nm, 30 min) to give compound **83** (68 mg, 50% yield) as a yellow solid. MS Calc.: 760; MS Found: 761 $[M+H]^+$.

(2S)-2-amino-4-((((2R,3S,4R,5R)-5-(6-amino-9H-purin-9-yl)-3,4-dihydroxytetrahydrofuran-2-yl)methyl)(2-(pyrrolidin-2-yl)ethyl)amino)butanoic acid (84, 1c)

To a solution of compound **83** (68 mg, 0.09 mmol) in DCM (2 mL) was added HBr (30% in AcOH) (0.5 mL). The reaction mixture was stirred at room temperature for 4 hours. The reaction mixture was concentrated. The residue was dissolved in 2 mL of MeoH and adjusted pH = 7 with Na_2CO_3 solution. The residue was concentrated and purified by prep-HPLC to give compound **84**, **1c** (17.3 mg, 28% yield) as a white solid. ^1H NMR (400 MHz, CD_3OD) δ: 8.25 (d, J = 6.0 Hz, 2H), 5.98 (d, J = 3.2 Hz, 1H), 4.63 (br s, 2H), 4.36-4.29 (m, 2H), 3.83-3.80 (m, 1H), 3.48-3.36 (m, 3H), 3.15-3.03 (m, 4H), 2.21 (br s, 1H), 2.11-1.81 (m, 7H), 1.6-1.42 (m, 1H). MS Calc.: 464; MS Found: 465 $[M+H]^+$. UV-purity at 254 nm = >95%.
